# Stafia‐1: a STAT5a‐Selective Inhibitor Developed via Docking‐Based Screening of in Silico O‐Phosphorylated Fragments

**DOI:** 10.1002/chem.201904147

**Published:** 2019-11-27

**Authors:** Kalaiselvi Natarajan, Daniel Müller‐Klieser, Stefan Rubner, Thorsten Berg

**Affiliations:** ^1^ Institute of Organic Chemistry Leipzig University Johannisallee 29 04103 Leipzig Germany

**Keywords:** biological activity, inhibitors, protein–protein interactions, SH2 domains, transcription factors

## Abstract

We present a new approach for the identification of inhibitors of phosphorylation‐dependent protein–protein interaction domains, in which phenolic fragments are adapted by in silico O‐phosphorylation before docking‐based screening. From a database of 10 369 180 compounds, we identified 85 021 natural product‐derived phenolic fragments, which were virtually O‐phosphorylated and screened for in silico binding to the STAT3 SH2 domain. Nine screening hits were then synthesized, eight of which showed a degree of in vitro inhibition of STAT3. After analysis of its selectivity profile, the most potent inhibitor was then developed to Stafia‐1, the first small molecule shown to preferentially inhibit the STAT family member STAT5a over the close homologue STAT5b. A phosphonate prodrug based on Stafia‐1 inhibited STAT5a with selectivity over STAT5b in human leukemia cells, providing the first demonstration of selective in vitro and intracellular inhibition of STAT5a by a small‐molecule inhibitor.

## Graphical abstract

**Creating and searching the virtual haystack**: In silico O‐phosphorylation of preselected natural product‐related fragments from the SCONP (structural classification of natural products) tree, virtual screening and chemical derivatization enabled the development of Stafia‐1, the first molecule shown to inhibit the transcription factor STAT5a with selectivity over the close homologue STAT5b.

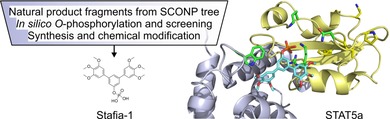

Protein–protein interactions mediate most biological processes, and their functional modulation by small molecules offers vast opportunities for basic research and drug development.^1^ However, protein–protein interactions represent challenging targets for small molecules, and design approaches for inhibitor development are rare.^2^

Phosphorylation‐dependent protein–protein interactions are mediated by the phosphorylated side chains of tyrosine, serine, and threonine residues, and play an important role in signal transduction. We recently proposed O‐phosphorylation of preselected natural products as an approach for the development of non‐peptidic and non‐reactive ligands of phosphorylation‐dependent protein–protein interactions.^3^ We used this approach to develop catechol bisphosphates^4^ as the first chemical entities that inhibit the phosphotyrosine‐dependent Src homology 2 (SH2) domain of the transcription factor STAT5b with high selectivity over the close homologue STAT5a.^5^ Both STAT5 proteins are constitutively activated in numerous human tumors.^6^ Selective inhibition of either STAT5 protein is desirable for the functional analysis of the non‐redundant functions of STAT5a and STAT5b,^7^ and would offer flexibility in tailoring the antitumor treatment strategy to individual human tumors. Small molecule STAT5a inhibitors with selectivity over STAT5b could also serve as therapeutic modalities for age‐related osteoporosis.^8^ However, no STAT5a inhibitors^3^, ^9^ with selectivity over STAT5b have been disclosed to date.

Here, we present virtual (in silico) O‐phosphorylation of preselected phenolic fragments of natural products,^10^ followed by docking‐based virtual screening, as a novel methodology for the identification of inhibitors of phosphotyrosine‐dependent protein–protein interaction domains. The initial virtual compound library was downloaded from the ZINC database^11^ as a collection of 10 369 180 structures. Filtering this database for structural elements described by the structural classification of natural products (SCONP) tree^10^ identified 799 335 compounds (Figure 1 A, step 1, Figure S1, and Supporting Methods in the Supporting Information). Further filtering for fragments with a phenol moiety and a molecular weight below 500 g Mol^−1^, and removal of certain reactive moieties (Figure 1 A, step 2, and Supporting Methods), narrowed down the selection to 85 021 compounds, which were then virtually O‐phosphorylated on their phenolic moiety by altering their SMILES string (Figure 1 A, step 3).^12^ Virtual screening of the O‐phosphorylated compounds against the STAT3 SH2 domain (PBD ID: 1BG1)^13^ with AutoDock Vina^14^ resulted in 1 114 compounds, which fulfilled predefined criteria for the distances between the phosphate groups of the molecules and the crucial STAT3 SH2 domain residues Arg609 and Lys591 (Figure 1 A, step 4, and Figure S2).^13^ After visual inspection of the binding poses, 9 molecules (**1**–**9**) were selected (Figure 1 A, step 5, Table S1), which display a variable degree of resemblance to natural products, depending on the size of the underlying natural product‐derived structural element from the SCONP tree.^10^ Molecules **1**–**9** were synthesized by O‐phosphorylation of commercially available or pre‐synthesized phenolic precursors, by a two‐step phosphorylation/debenzylation process (Figure 1 A, step 6, Table S1, and Supporting Information), and tested in a fluorescence polarization (FP) assay against the STAT3 SH2 domain (Figure 1 A, step 7).^15^ Eight of the O‐phosphorylated molecules **1**–**9** showed a degree of STAT3 inhibition, with **1**–**3** inhibiting STAT3 by more than 40 % at 100 μm (Table S1). **1** docked into the STAT3 SH2 domain with its phosphate group in close proximity to Arg609 and Lys591 (Figure 1 B). Although screening had been performed with the aim of identifying inhibitors of the STAT3 SH2 domain, analysis of specificity profiles revealed that several compounds, including **1**, were more active against STAT5a and STAT5b^16^ than against STAT3 (Table S1). This suggests that the docking approach may not be sensitive enough to clearly discriminate between the STAT family members. Since selective STAT5a inhibitors have not yet been reported, we decided to optimize **1** for binding to STAT5a, rather than STAT3. Compound **1** was chosen as a starting point for inhibitor development due to its lack of reactive functional groups, and its *m*‐terphenyl scaffold should allow for flexible modifications through Suzuki coupling reactions.

**Figure 1:**
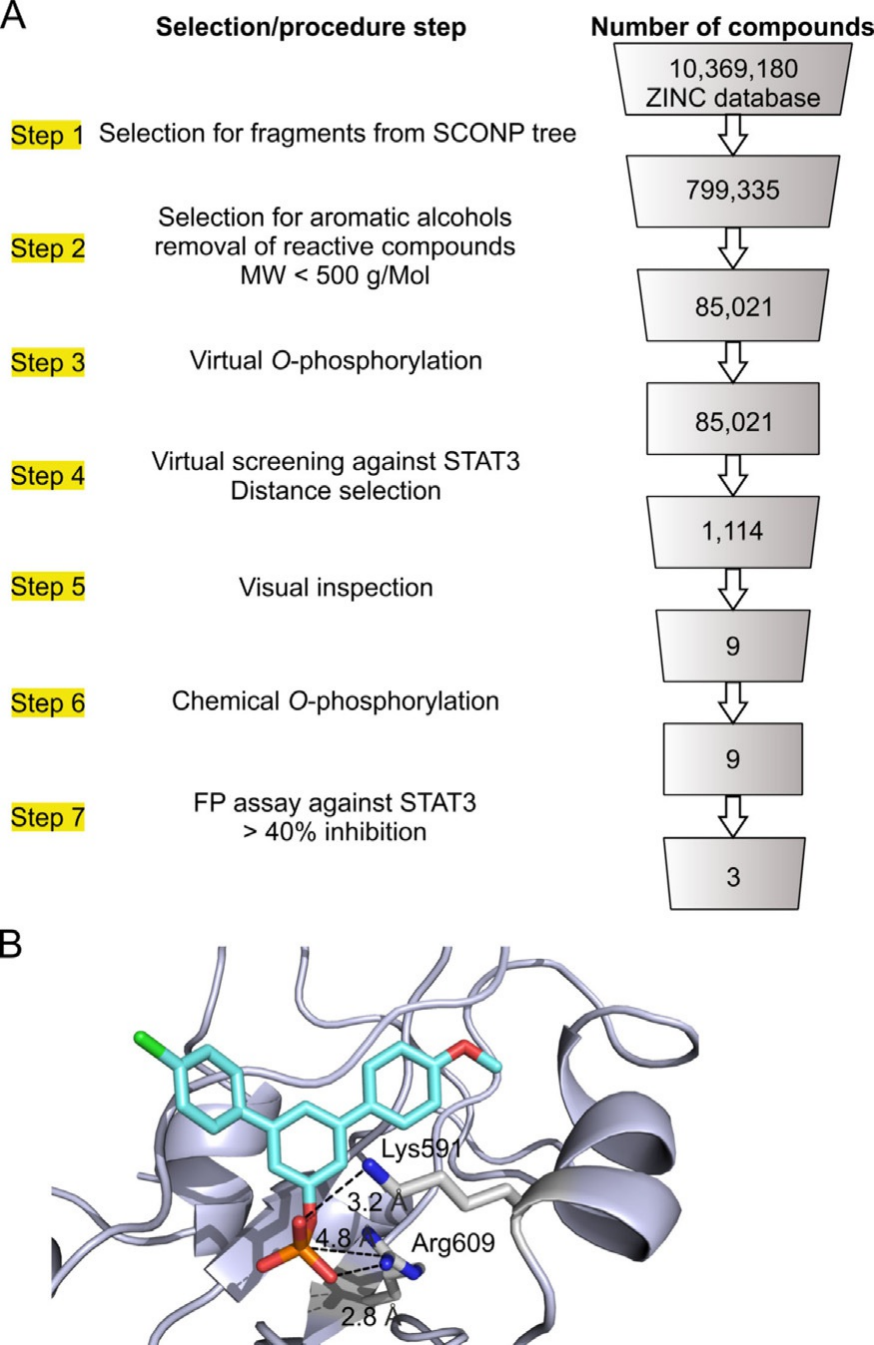
A) Selection criteria and procedures applied for docking‐based screening of virtually O‐phosphorylated natural product‐related libraries. B) Docking pose of **1** in the STAT3 SH2 domain with predicted distances between the phosphate group of **1** and STAT3 Lys591 and Arg609. The Figure was generated using PyMOL.^17^

Dose‐dependent analysis of **1** in FP assays already showed a slight preference of **1** for STAT5a over STAT5b, which was lost by 4,4′′‐dichloro‐substitution (compound **10**, Tables 1 and S2), but improved for the unsubstituted *m*‐terphenyl phosphate **11** and the 4,4′′‐dimethoxy derivative **12**. Activity of **12** was phosphorylation‐dependent, as indicated by the lack of activity of the unphosphorylated precursor **12 a**. Given the beneficial effects of the methoxy groups, the effect of di‐, tetra‐, and hexa‐methoxy substitution on the symmetrical *m*‐terphenyl phosphates was further examined. In the disubstituted series **12**–**14**, 4,4′′‐disubstitution (**12**) was preferable over 3,3′′‐disubstitution (compound **13**) and 2,2′′‐disubstitution (compound **14**). In the tetrasubstituted series **15**–**17**, the 3,3′′,4,4′′‐substituted compound **15** was more selective for STAT5a over STAT5b than the 2,2′′,3,3′′‐substituted isomer **16** and the 3,3′′,5,5′′‐substituted isomer **17**, and also more selective for STAT5a than **12**. Attempts to improve STAT5a selectivity of **15** by rigidifying the alkoxy substituents (compounds **18** and **19**) were not successful. In the hexasubstituted series **20**–**21**, the 3,3′′,4,4′′,5,5′′‐substituted compound **20** was significantly more active against STAT5a than **21**. Compound **20**, which was synthesized in three synthetic steps (Figure 2 A), inhibited STAT5a (IC_50_=22.2±3.6 μm, *K*_i_=10.9±1.8 μm) with at least 9‐fold selectivity over STAT5b (37±5 % inhibition at 200 μm, the highest concentration tested) and higher selectivity against other STAT family members (Figure 2 B). Exchanging the methoxy substituents of **20** for fluorine (compound **22**) maintained activity against STAT5a but reduced selectivity. Deletion of one phenyl ring of **20** caused a 5‐fold drop in activity against STAT5a (compound **23**), demonstrating the necessity of the *m*‐terphenyl phosphate moiety for STAT5a inhibition.

**Table 1:** Structures of synthesized compounds and activities against STAT5a, STAT5b, and STAT3.

Comp.	Structure	STAT5a *K*_i_ [μm] or inhibition [%] at 200 μm	STAT5b *K*_i_ [μm] or inhibition [%] at 200 μm	STAT3 *K*_i_ [μm] or inhibition [%] at 200 μm
**1**	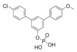	19.7±0.9 μm	24.5±1.6 μm	59.2±5.0 μm
**10**	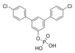	20.9±1.6 μm	18.7±0.02 μm	18.9±0.9 μm
**11**	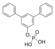	23.7±2.1 μm	48.4±2.0 μm	48±1 % inhibition
**12**	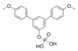	13.8±0.8 μm	38.5±0.2 μm	77.1±5.8 μm
**12 a**	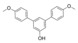	no inhibition	no inhibition	no inhibition
**13**	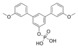	16.0±2.8 μm	43.1±1.5 μm	51±2 % inhibition
**14**	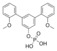	43.9±3.0 μm	78.6±0.4 μm	28±1 % inhibition
**15**	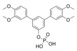	16.5±2.5 μm	44±3 % inhibition	43±2 % inhibition
**16**	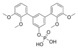	22.3±4.8 μm	40±2 % inhibition	15±2 % inhibition
**17**	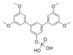	17.1±2.2 μm	36.5±2.5 μm	50±3 % inhibition
**18**	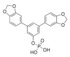	14.0±2.1 μm	28.0±0.5 μm	63.8±2.3 μm
**19**	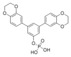	11.1±1.9 μm	35.4±2.8 μm	51.2±2.0 μm
**20**	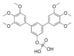	10.9±1.8 μm	37±5 % inhibition	27±2 % inhibition
**21**	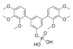	55.2±4.9 μm	28±2 % inhibition	15±3 % inhibition
**22**	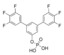	10.8±1.1 μm	23.3±0.4 μm	50.4±4.2 μm
**23**	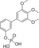	53.0±2.1 μm	39±1 % inhibition	40±1 % inhibition

**Figure 2:**
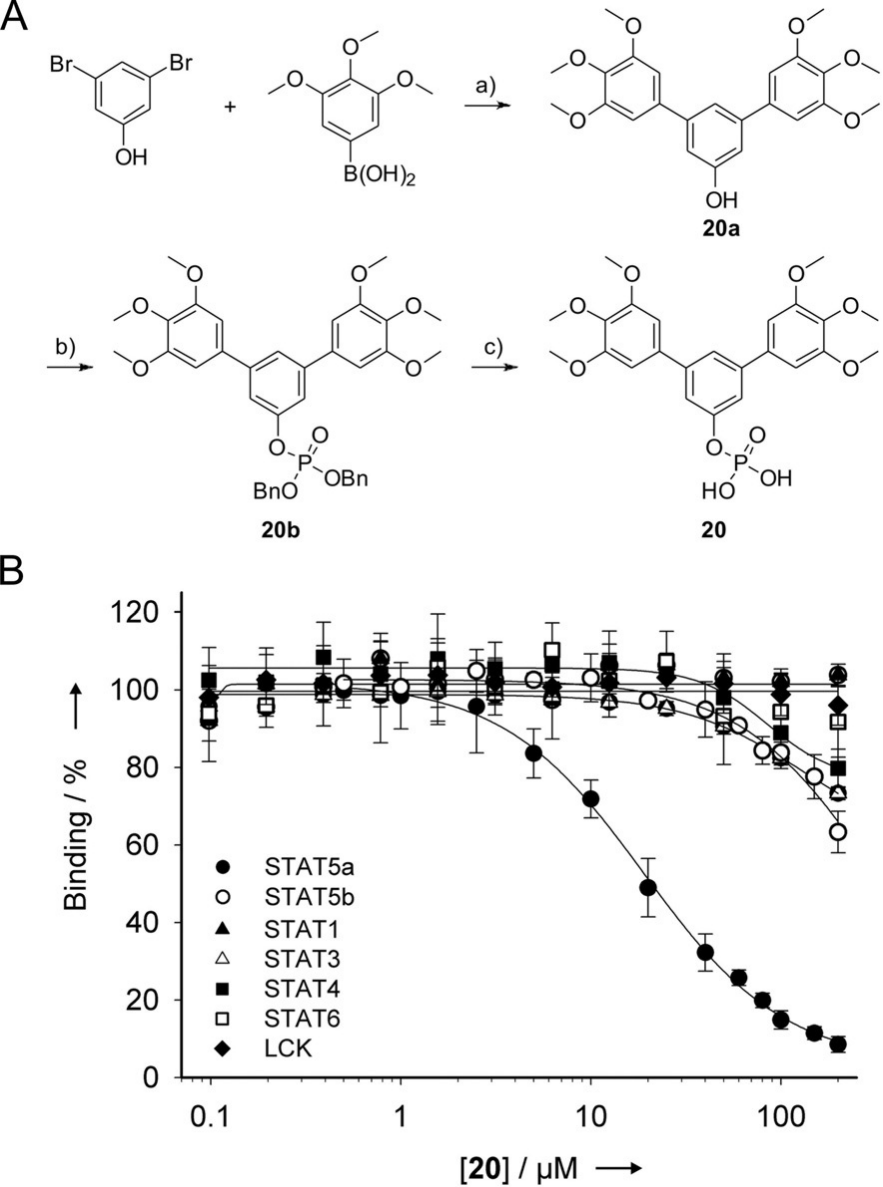
A) Synthesis of **20**. a) Na_2_CO_3_, Pd(PPh_3_)_4_, H_2_O/MeOH, 64 %; b) (BnO)_2_P(O)H, CCl_4_, DIEA, DMAP, CH_3_CN, 58 %; c) Pd/C, H_2_, EtOH; 98 %. DIEA=*N,N*‐diisopropylethylamine; DMAP=4‐dimethylaminopyridine. B) Activities of **20** against the SH2 domains of STAT proteins and Lck.

The SH2 domains of STAT5a and STAT5b are 93 % identical on the amino acid level, with only 6 of 91 amino acids differing (Figure S3). To investigate the molecular origin of the specificity of **20** for STAT5a over STAT5b, we used wild‐type and point mutant STAT5 proteins in FP assays. The activity of **20** against the point mutant STAT5b Gln636Pro/Met639Asn/Phe640Leu/Met644Lys/Asn664Ser/Tyr679Phe (dubbed STAT5b‐6M),^18^ in which the six amino acids of the STAT5b SH2 domain which differ from those in the STAT5a SH2 domain have been replaced by their STAT5a counterparts (Figure S3), was only marginally increased (46±8 % inhibition at 200 μm) compared to wild‐type STAT5b (37±5 % inhibition at 200 μm), and thus approximately 10‐fold lower than the activity against wild‐type STAT5a (*K*_i_=10.9±1.8 μm, IC_50_=22.2±3.6 μm, Figure 3 A and Table S3). This indicated that factors outside the SH2 domain must play a significant role for binding of **20** to STAT5a.

**Figure 3:**
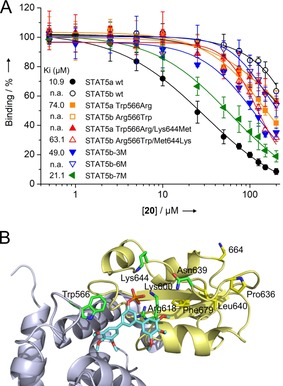
A) Activity of **20** against wild‐type and mutant STAT5 proteins. n.a.: not applicable. B) Docking pose of **20** into the X‐ray structure of murine STAT5a (PBD 1Y1U).^23^ SH2 domain shown in yellow, linker domain in blue; the side chains of amino acids defined as flexible in the docking are shown with carbon atoms in green. Divergent amino acids in the SH2 domain defined as rigid are shown with carbon atoms in yellow. Asn664 is replaced by Ser in human STAT5a. The Figure was generated using PyMOL.^17^

We recently described the divergent amino acids in position 566 of the STAT5 linker domain (Trp in STAT5a, Arg in STAT5b, Figure S3), adjacent to the SH2 domain, as the crucial determinant for selective STAT5b inhibition by small molecules.^18^, ^19^ To investigate the role of STAT5a Trp566, we tested **20** against the crossover point mutant STAT5a Trp566Arg, and found its activity to be reduced by 7‐fold (*K*_i_=74.0±7.0 μm, Figure 3 A and Table S3) as compared to wild‐type STAT5a. Although this suggests a role for Trp566 in binding to **20**, it is not the sole determinant of specificity, since the reverse mutant STAT5b Arg566Trp (43±7 % inhibition at 200 μm) was only marginally more inhibited than wild‐type STAT5b (37±5 % inhibition at 200 μm) by **20** (Figure 3 A and Table S3). However, the combined presentation of the STAT5a SH2 domain and Trp566 in the context of STAT5b, as represented by the mutant STAT5b Arg566Trp/Gln636Pro/Met639Asn/Phe640Leu/Met644Lys/Asn664Ser/Tyr679Phe (dubbed STAT5b‐7M),^18^ almost restored binding (*K*_i_=21.1±4.4 μm, Figure 3 A and Table S3). These data indicate that recognition of **20** by STAT5a depends on both the SH2 domain and Trp566. The remaining twofold activity difference of **20** between STAT5a (*K*_i_=10.9±1.8 μm) and STAT5b‐7M (*K*_i_=21.1±4.4 μm, Figure 3 A and Table S3) may be mediated by allosteric cross‐communication with divergent amino acid positions in the linker domain,^20^ the DNA binding domain,^21^ or the coiled‐coil domain.^22^

Docking of **20** into the crystal structure of STAT5a^23^ using AutoDock FR,^24^ with the side chains of amino acids in the phosphotyrosine binding pocket or its immediate vicinity (Trp566, Lys600, Arg618, Asn639, and Lys644) defined as flexible, placed one of the terminal phenyl rings of **20** near STAT5a Trp566 (Figure 3 B), consistent with π‐stacking interactions and with hydrogen bonding between the proton attached to the indole nitrogen of Trp566 and at least one of the methoxy groups of **20**. The phosphate group of **20** is predicted to form electrostatic interactions with Lys600 and Arg618, which are identical in STAT5a and STAT5b, in a manner similar to that of phosphotyrosine‐containing ligands.^13^ Selectivity‐conferring electrostatic interactions may arise from interaction with the side chain of STAT5a Lys644, which is replaced by methionine in STAT5b. The relevance of STAT5a Lys644 for binding of **20** was demonstrated by reduced inhibition of the crossover double mutant STAT5a Trp566Arg/Lys644Met (44±4 % inhibition at 200 μm) as compared to STAT5a Trp566Arg (*K*_i_=74.0±7.0 μm, Figure 3 A and Table S3). Conversely, the activity of **20** against the crossover mutant STAT5b Arg566Trp/Met644Lys was enhanced (*K*_i_=63.1±4.2 μm) as compared to STAT5b Arg566Trp (43±7 % inhibition at 200 μm, Figure 3 A and Table S3). The triple crossover mutant STAT5b Arg566Trp/Met639Asn/Met644Lys (dubbed STAT5b‐3M, *K*_i_=49.0±5.4 μm, Figure 3 A and Table S3) was somewhat more susceptible to inhibition by **20** than the double mutant STAT5b Arg566Trp/Met644Lys, suggesting a small contribution of Asn639 (present in STAT5a) to binding. The remaining 2.5‐fold activity gap between the triple mutant STAT5b‐3M and the 7‐fold mutant STAT5b‐7M (*K*_i_=21.1±4.4 μm) points towards an allosteric contribution by one or more of the remaining four STAT5a/b SH2 domain divergent amino acids, which are further removed from the putative binding site of **20**.

We note that in the available crystal structure of STAT5a,^23^ the position of the side chain of Lys600 is not suitable for phosphate group binding (Figure S4). Lys600 is required for binding of phosphotyrosine in peptidic SH2 domain ligands, and the unsuitable orientation is presumably a consequence of the fact that this STAT5a structure derives from the protein without a bound ligand. In contrast, in the crystal structure of tyrosine‐phosphorylated STAT3 homodimers,^13^ the important amino acid side chains adopt a suitable conformation for phosphate binding (Figure S4). The virtual screening of STAT3 described in this study was achieved using AutoDock Vina,^14^ which in our experience works best with rigid proteins. In contrast, STAT5a docking requires an approach with flexible amino acid side chains, such as AutoDock FR,^24^ which was used for rationalizing the binding mode of **20** (Figure 3 B). However, AutoDock FR is less adaptable to virtual screening of large chemical libraries than AutoDock Vina.

We synthesized the phosphonates **24**–**26**, which are not susceptible to phosphatase‐catalyzed cleavage, as derivatives of the phosphate **20** (Figure 4 A, Figures S5‐S7).^25^ The methylene phosphonate **24** (*K*_i_=75±13 μm) was slightly less active against STAT5a than the difluoromethylene phosphonate **25** (*K*_i_=52±3 μm, Figure 4 B). The most potent derivative was the monofluoromethylene phosphonate **26** (*K*_i_=28±3 μm), which retained selectivity over STAT5b and other STAT proteins (Figure 4 B, C). This represents a rare case in which a monofluoromethylene phosphonate displays higher activity against an SH2 domain than the corresponding difluoromethylene phosphonate,^26^ despite the introduction of an uninduced chiral center by fluorine monosubstitution.

**Figure 4:**
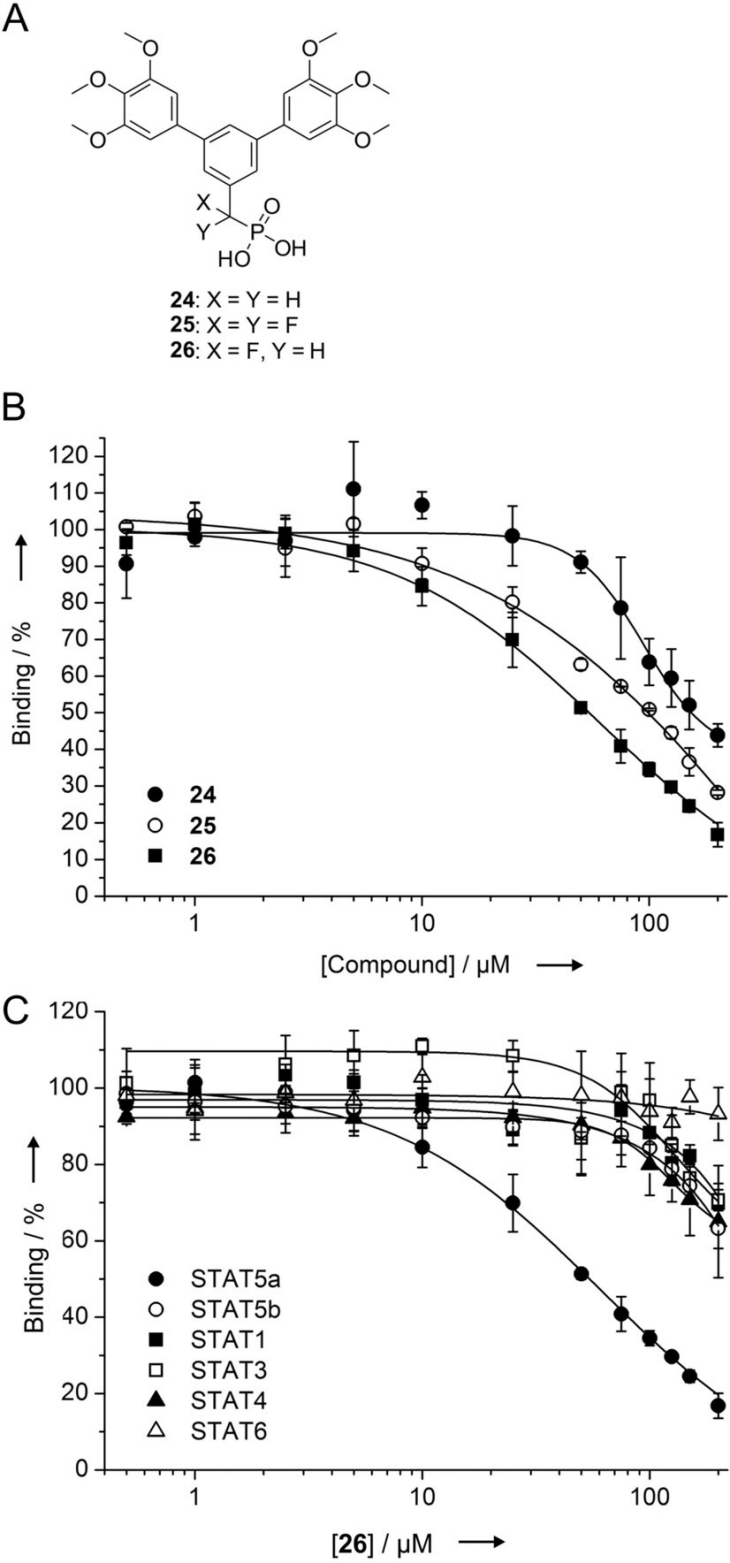
A) Structure of phosphonates **24**–**26**. B) Activities of **24**–**26** against STAT5a in FP assays. C) Selectivity profile of **26** against STAT proteins.

Phosphonates are typically not cell‐permeable, being negatively charged at physiological pH. To mask its negative charges, **26** was converted to the pivaloyloxymethyl prodrug **27** (Figure 5 A). Pivaloyloxymethyl prodrugs of organic phosphates4a–4c and phosphonates^27^ are cleaved by intracellular esterases, releasing the bioactive parent compounds. **27** was tested for its ability to prevent phosphorylation of STAT5a in K562 cells, a human chronic myelogenous leukemia cell line. In these cells, both STAT5a and STAT5b are constitutively phosphorylated on Tyr694 (STAT5a) and Tyr699 (STAT5b), respectively, by Bcr‐Abl. Since phosphorylation is dependent on the function of the SH2 domain, a STAT5 SH2 domain inhibitor will reduce the ability of STAT5 to be tyrosine phosphorylated by Bcr‐Abl (Figure 5 B). Commercially available antibodies that recognize STAT5a/b only in their tyrosine phosphorylated state do not differentiate between the two STAT5 proteins, so we ectopically expressed fusion proteins of either STAT5a or STAT5b and GFP to distinguish unambiguously between the two STAT5 proteins.4a–4c In the presence of the prodrug **27**, phosphorylation of STAT5a Tyr694 in the STAT5a‐GFP fusion protein was inhibited in a dose‐dependent manner (Figure 5 C, E). In contrast, phosphorylation of Tyr699 in the STAT5b‐GFP construct was only minimally affected (Figure 5 D, F), showing that the selectivity of **26** for STAT5a over STAT5b is maintained in the cellular environment. In contrast, the STAT5 inhibitor AC‐4–130,^28^ for which no preference for either STAT5 protein has been reported, and the Bcr‐Abl inhibitor imatinib^29^ do not discriminate between STAT5a and STAT5b in this assay (Figure S8). Phosphorylation of endogenous STAT5a/b was reduced to a substantially lower degree than that of STAT5a‐GFP (Figure S9) by **27**. This is consistent with our previous observations, which indicate that the majority of endogenous phospho‐STAT5 in K562 cells is STAT5b.4a–4c

**Figure 5:**
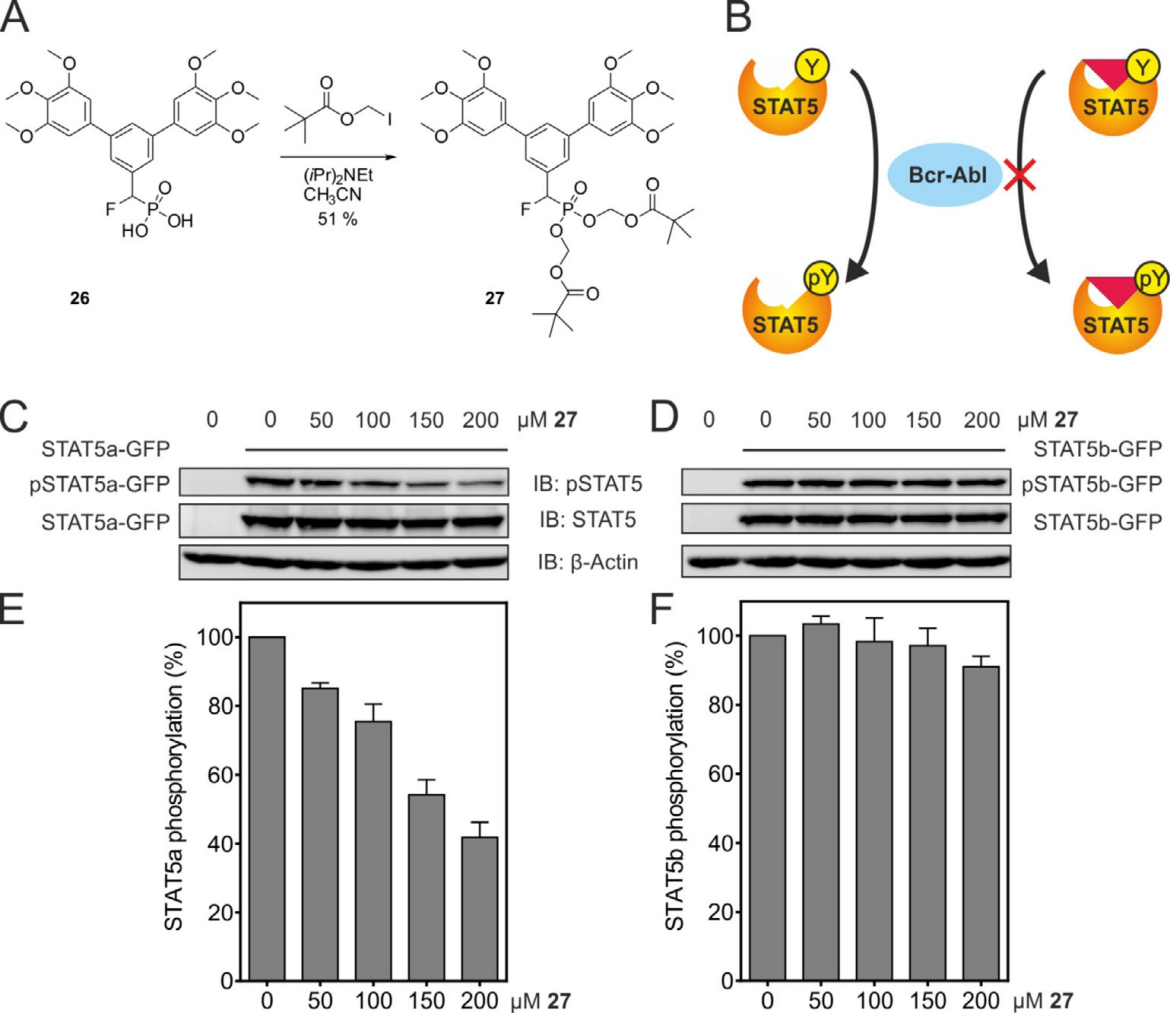
A) Synthesis of prodrug **27**. B) Tyrosine phosphorylation of STAT5a/b by Bcr‐Abl is inhibited by a ligand of the SH2 domain. C) Effect of **27** on phosphorylation of STAT5a in STAT5a‐GFP‐transfected K562 cells, and D) on phosphorylation of STAT5b in STAT5b‐GFP‐transfected K562 cells. E, F) Quantitation of the data shown in C) (*n*=4), and D) (*n*=3), respectively. Phospho‐STAT5a/b‐GFP levels are normalized against total STAT5a/b‐GFP. All error bars represent standard deviations (s.d.).

In conclusion, we present docking‐based screening of in silico O‐phosphorylated natural product‐derived fragments as a novel method for identifying lead structures for the development of inhibitors of phosphorylation‐dependent protein–protein interaction domains, which are of crucial importance to cellular signaling. Whereas virtual screening of phosphonates and phosphates against a phosphorylation‐dependent protein–protein interaction domain has been reported,^30^ our work represents the first case in which the virtual screening library itself is generated by in silico O‐phosphorylation. Application of this concept to the STAT3 SH2 domain resulted in the moderate STAT3 inhibitor **1**, which was then discovered to preferentially target STAT5. Analysis of structure–activity relationships led to the development of **20**, the first inhibitor of STAT5a which displays high selectivity over STAT5b and other STAT family members. We dubbed **20** “Stafia‐1” (STAT five a inhibitor 1). The use of wild‐type and point mutant STAT5 proteins demonstrated that both the SH2 domain and Trp566 in the adjacent linker domain contribute to selective recognition of Stafia‐1 by STAT5a. The cell‐permeable prodrug **27**, based on the Stafia‐1‐derived monofluoromethylene phosphonate **26**, inhibited tyrosine phosphorylation of STAT5a with selectivity over STAT5b in cultured human leukemia cells, and represents a valuable tool to define the non‐redundant molecular functions of the two highly homologous transcription factors in tumor cells.^7^ Selective inhibition of STAT5a by **27**, especially in direct comparison with selective inhibition of STAT5b by catechol bisphosphate‐based prodrugs such as Pomstafib‐2,4c would allow for dissection of the target genes of STAT5a and STAT5b with high temporal control.^7^ Our data provide the first demonstration that selective targeting of STAT5a over STAT5b is feasible both in vitro and in cells.

## Conflict of interest

The authors declare no conflict of interest.

## Supporting information

As a service to our authors and readers, this journal provides supporting information supplied by the authors. Such materials are peer reviewed and may be re‐organized for online delivery, but are not copy‐edited or typeset. Technical support issues arising from supporting information (other than missing files) should be addressed to the authors.

Supplementary
