# Supplementary material for: Stafia‐1: a STAT5a‐Selective Inhibitor Developed via Docking‐Based Screening of in Silico O‐Phosphorylated Fragments
Source: Chemistry. 2019 Nov 27;26(1):148–54. doi: 10.1002/chem.201904147 (PMC6973011; doi:10.1002/chem.201904147)
Supplement: Supplementary file 1 — Supplementary [file CHEM-26-148-s001.pdf]

CHEMISTRY
A European Journal

Supporting Information

Stafia-1: a STAT5a-Selective Inhibitor Developed via Docking-Based Screening of in Silico O-Phosphorylated Fragments

Kalaiselvi Natarajan, Daniel Müller-Klieser+, Stefan Rubner+, and Thorsten Berg*[a]

chem_201904147_sm_miscellaneous_information.pdf

Table of Contents

Figure S1 2
Figure S2 2
Figure S3 3
Figure S4 3
Figure S5 4
Figure S6 4
Figure S7 5
Figure S8 5
Figure S9 6
Table S1 7
Table S2 8
Table S3 9
Library generation and virtual screening 9
Recombinant proteins 10
Fluorescence Polarization assays 10
Cell culture 11
Transfection and Western Blot 11
General synthetic methods 12
Synthesis and spectroscopic characterization of compounds 13
NMR spectra 50
Supporting references 54

Figure S1

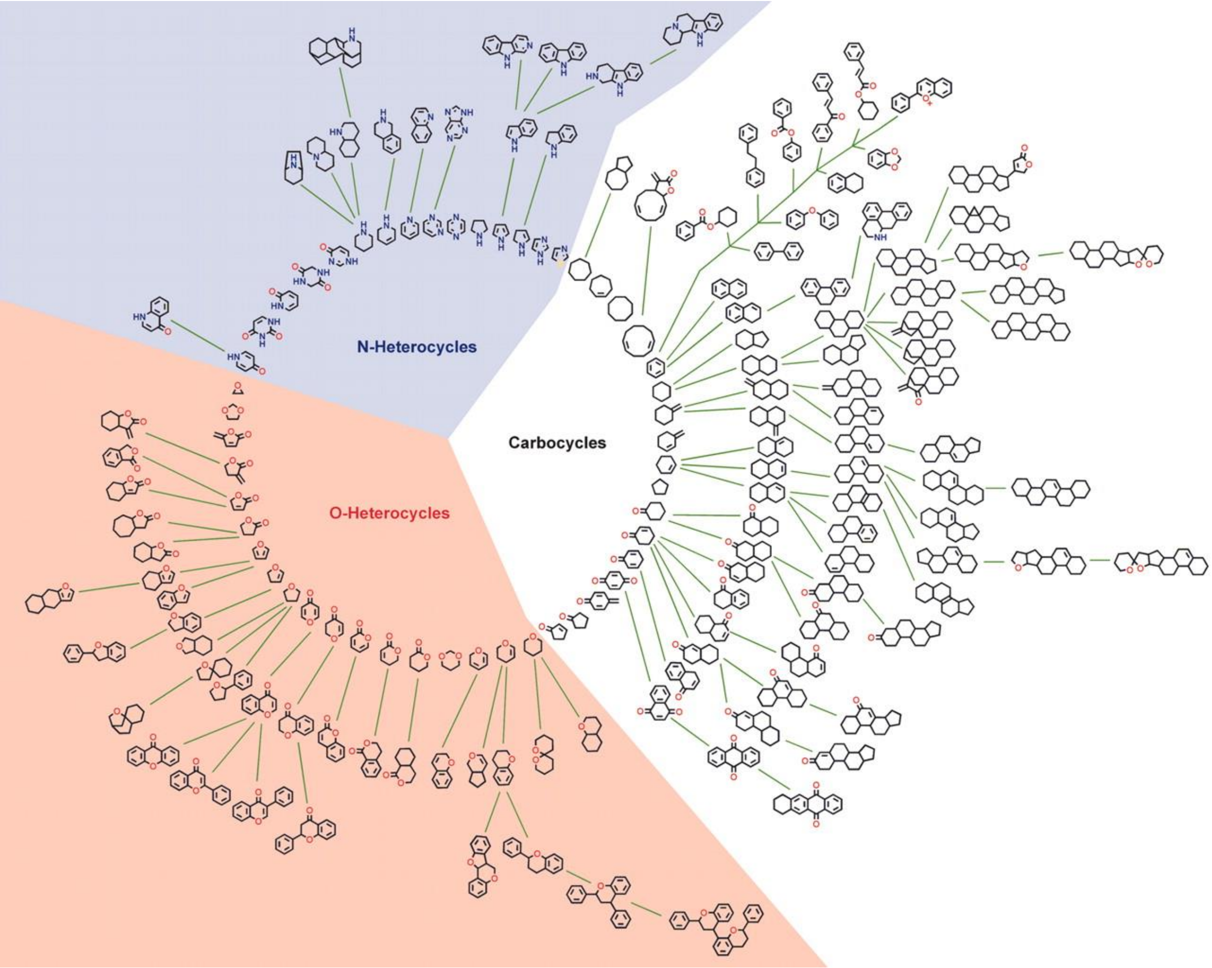

Figure S1. The Structural Classification of Natural Products (SCONP) tree. Reproduced from ref.[1] Copyright 2005 National Academy of Sciences.

Figure S2

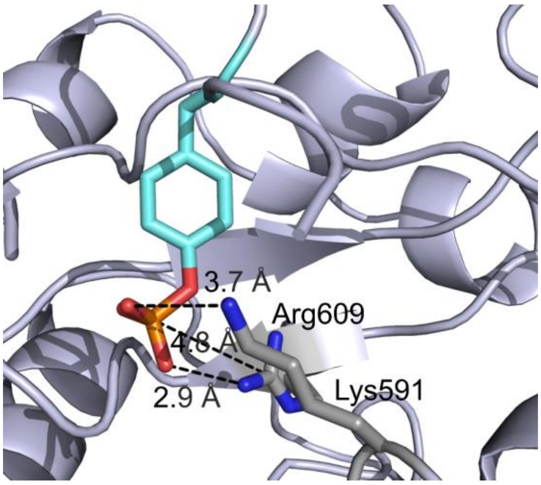

Figure S2. X-ray structure of the STAT3 dimer (PDB: 1BG1).[2] Close-up of binding of the phosphotyrosine residue of one STAT3 unit to the SH2 domain of the other STAT3 unit. Distances between the phosphate group of phosphotyrosine and the side chains of STAT3 amino acids Lys591 and Arg609 are shown. The figure was generated using PyMOL.[3]

Figure S3

A) SH2 domains

STAT5a 593 AILGFVNKQQAHDLLINKPDGTFLLRFSDSEIGGITIAWKFDS**P**ER**NL**WNL**K**PFTTRDFSIRSLADRL

STAT5b 593 AILGFVNKQQAHDLLINKPDGTFLLRFSDSEIGGITIAWKFDS**Q**ER**MF**WNL**M**PFTTRDFSIRSLADRL

STAT5a GDL**S**YLIYVFPDRPKDEV**F**SKYY 683

STAT5b GDL**N**YLIYVFPDRPKDEV**Y**SKYY 683

B) Linker domains

STAT5a 471 HGSQD**H**NATATVLWDNAFAEPGRVPFAVPDKVLWPQLCEALNMKFKAEVQSNRGLTKENLVFLAQKLFNN

STAT5b 471 HGSQD**N**NATATVLWDNAFAEPGRVPFAVPDKVLWPQLCEALNMKFKAEVQSNRGLTKENLVFLAQKLFNN

STAT5a SSSHLEDYSGLSVSWSQFNRENLPG**W**NYTFWQWFDGVMEVLKKH**H**KPHWNDG 592

STAT5b SSSHLEDYSGLSVSWSQFNRENLPG**R**NYTFWQWFDGVMEVLKKH**L**KPHWNDG 592

Figure S3. Amino acid sequence of the A) SH2 domains (amino acids 593 – 683) and B) linker domains (amino acids 471-592) of STAT5a and STAT5b.[4]

Figure S4

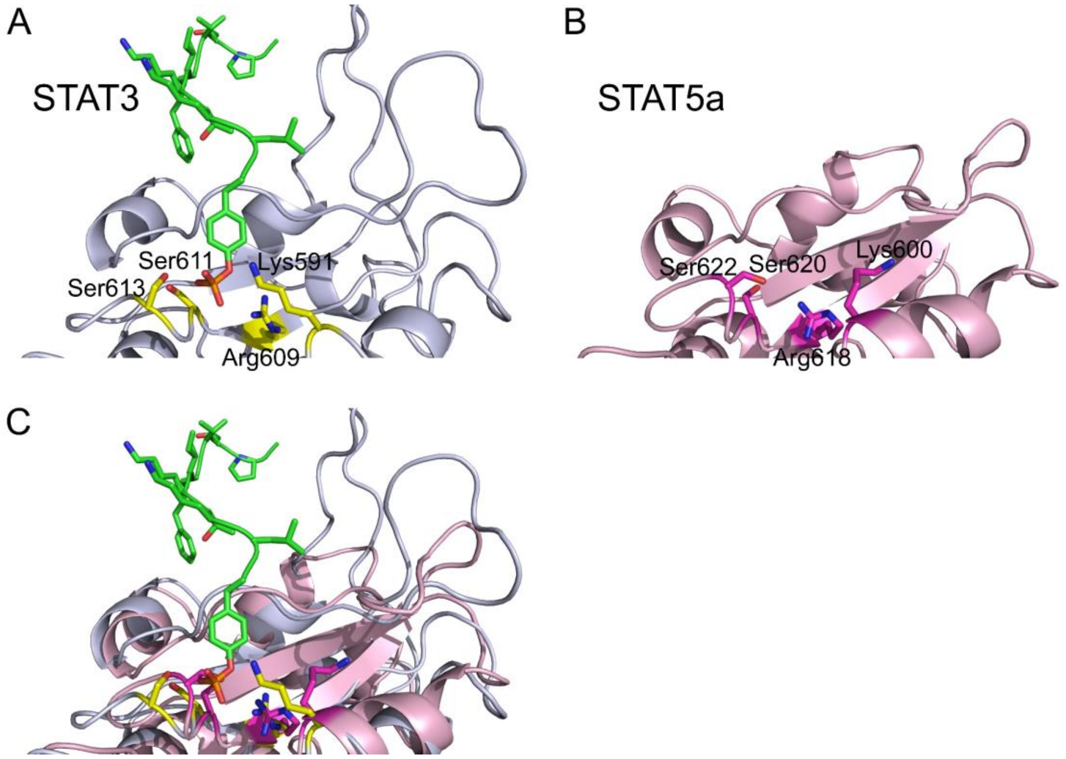

Figure S4. A) Close-up of the X-ray structure of tyrosine-phosphorylated STAT3 homodimers (PDB: 1BG1)[2] used for virtual screening. The phosphotyrosine residue of one STAT3 unit binds to the SH2 domain of the other STAT3 subunit. Binding of the phosphate group is mediated by Lys591, Arg609, Ser611, and Ser613. B) In the X-ray structure of STAT5a (PDB ID: 1Y1U),[5] the corresponding lysine residue (STAT5a Lys600) is not in a suitable position for binding of phosphotyrosine or mimetics thereof, requiring side chain flexibility to be used in molecular docking approaches. C) Overlay of A) and B).

Figure S5

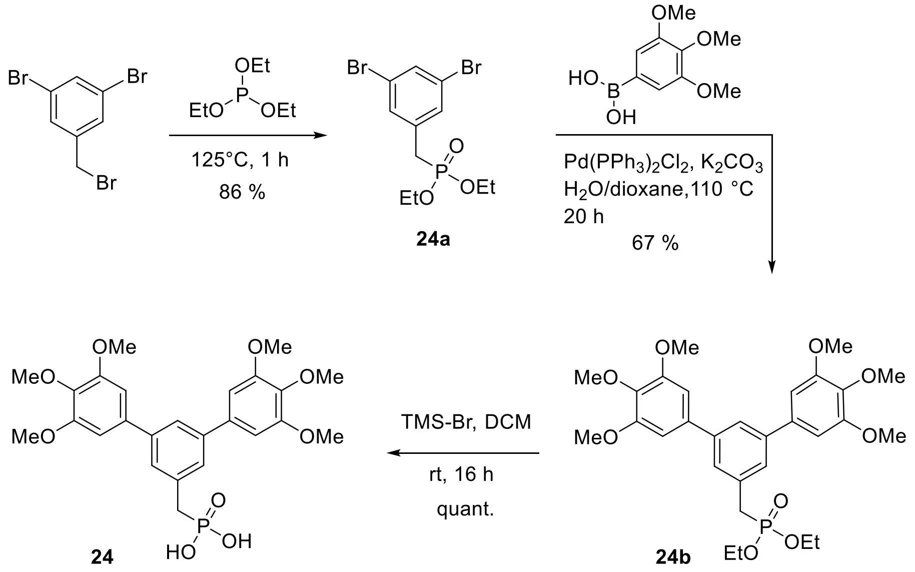

Figure S5. Synthesis of methylene phosphonate **24**.

Figure S6

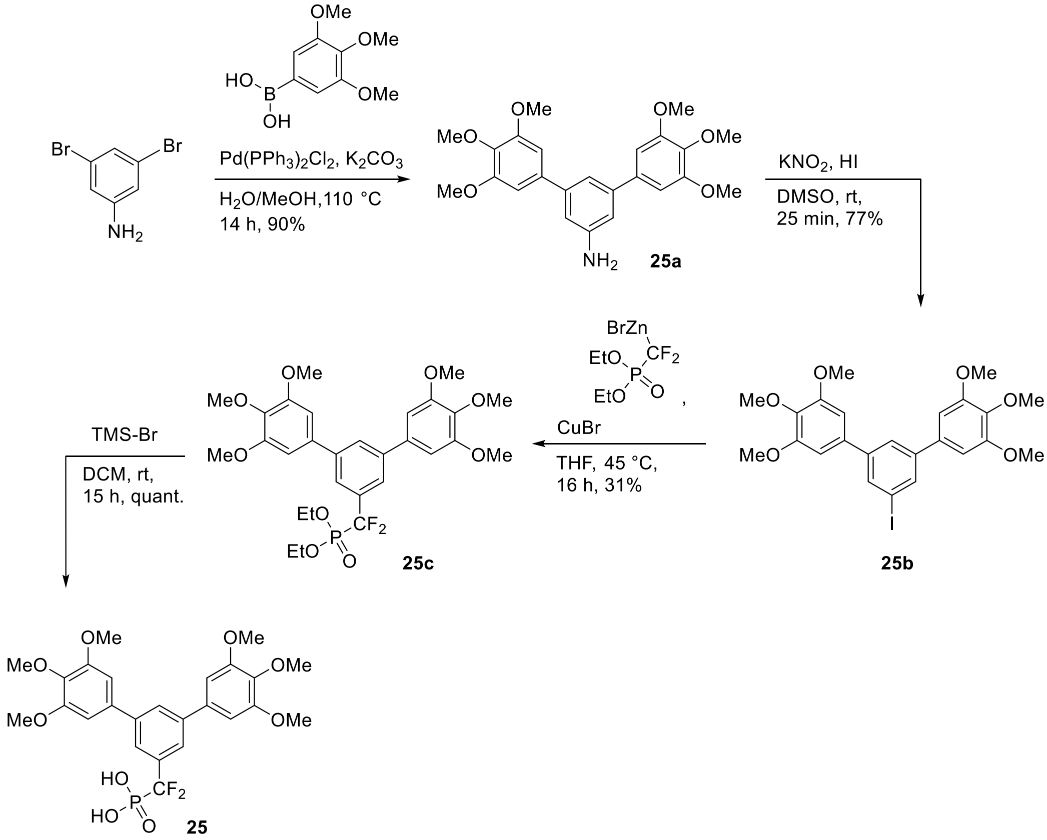

Figure S6. Synthesis of difluoromethylene phosphonate **25**.

Figure S7

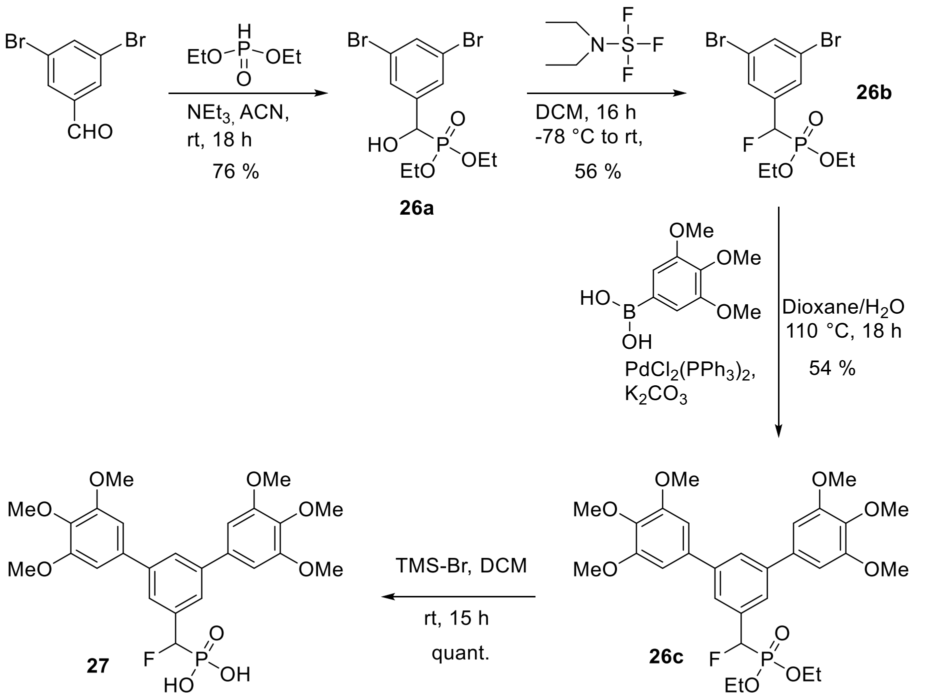

Figure S7. Synthesis of monofluoromethylene phosphonate **26**.

Figure S8

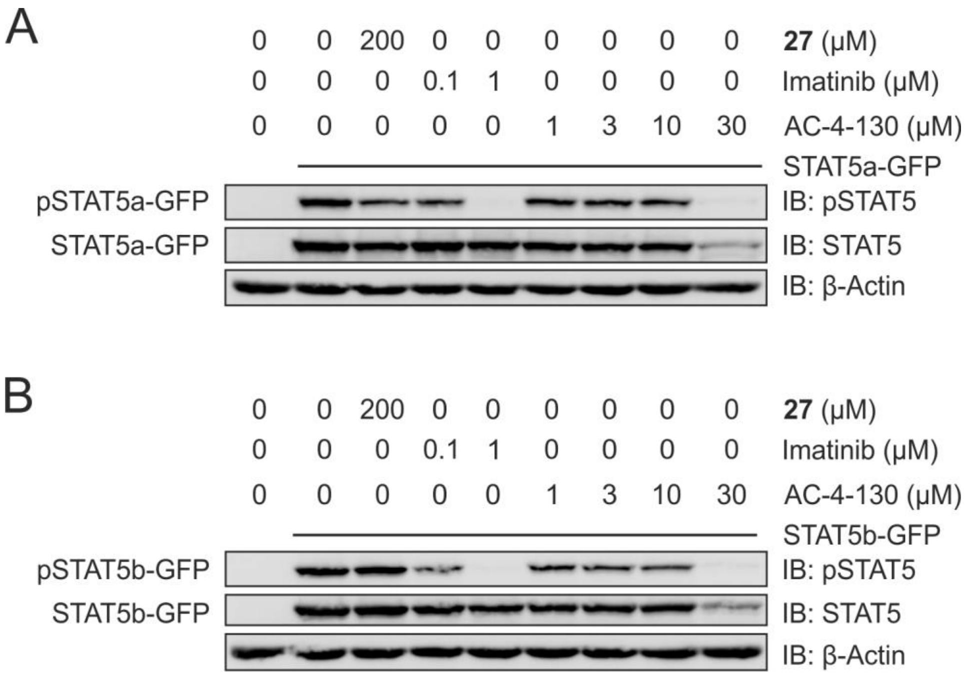

Figure S8. Comparative analysis of the effect of **27**, the Bcr-Abl inhibitor imatinib (Cell Signaling, #9084), and the STAT5 inhibitor AC-4-130 (Biotrend, Germany, #AOB36422) on STAT5 phosphorylation. Effect of the test compounds after 4 h at the indicated concentrations on phosphorylation of A) STAT5a in STAT5a-GFP-transfected K562 cells (n = 3), and B) STAT5b in STAT5b-GFP-transfected K562 cells (n = 2).

Figure S9

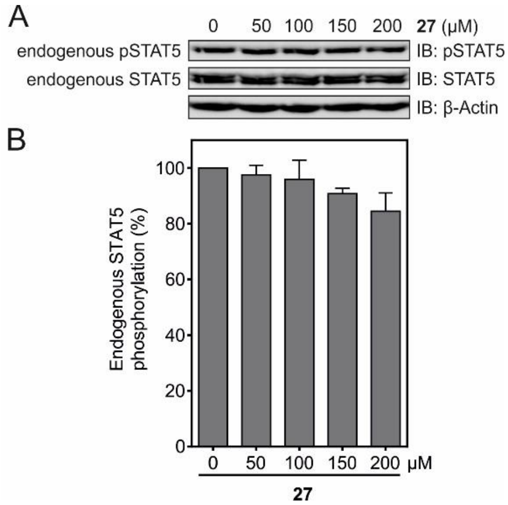

Figure S9. A) Effect of **27** on tyrosine phosphorylation of endogenous total STAT5 in STAT5a/b-GFP-transfected K562 cells. B) Quantitation of data as shown in A), with phosphorylated STAT5 levels normalized against total STAT5. Error bars represent the standard deviations from 6 independent experiments.

Table S1

No.	Structure	ZINC ID of phenolic precursor	Structural element from SCONP tree	Inihibition of STAT3 at 100 µM (%)	Inihibition of STAT5a at 100 µM (%)	Inihibition of STAT5b at 100 µM (%)
**1**	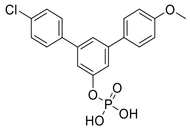	ZINC04088965	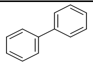	43±5	73±5	73±3
**2**	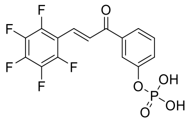	ZINC08322400	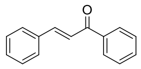	43±2	51±4	37±2
**3**	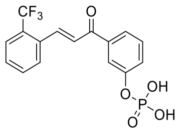	ZINC13135442	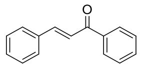	53±3	37±9	31±3
**4**	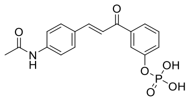	ZINC12974312	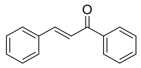	29±7	57±2	52±4
**5**	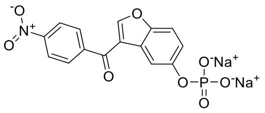	ZINC00446124	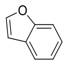	26±8	87±2	88±1
**6**	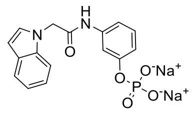	ZINC40266599	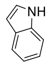	11±6	46±6	24±14
**7**	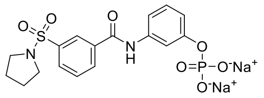	ZINC00837458	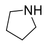	3±2	26±17	4±9
**8**	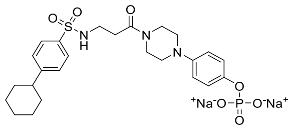	ZINC09549077	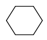	33±2	65±5	63±1
**9**	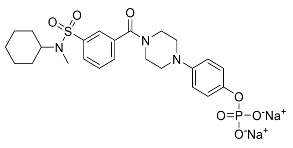	ZINC09223221	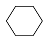	33±5	41±8	23±8

Table S1: Structures of synthesized *O*-phosphorylated screening hits and activities against STAT3, STAT5a, and STAT5b.

Table S2

No.	Structure	STAT5a<br>IC_{50} (µM)	STAT5b<br>IC_{50} (µM)	STAT3<br>IC_{50} (µM)
**1**	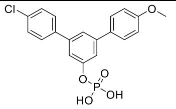	39.9 ± 1.9	49.4 ± 3.3	120 ± 10
**10**	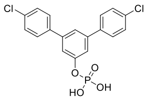	42.2 ± 3.3	37.7 ± 0.05	38.4 ± 1.7
**11**	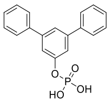	47.9 ± 4.3	95 ± 3	-
**12**	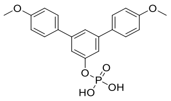	27.9 ± 1.7	77.5 ± 0.4	156 ± 12
**13**	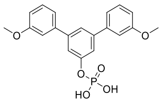	32.3 ± 5.7	86.7 ± 3.1	-
**14**	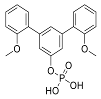	88.7 ± 6.1	158 ± 1	-
**15**	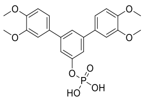	33.4 ± 5.2	-	-
**16**	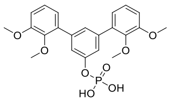	45.1 ± 9.8	-	-
**17**	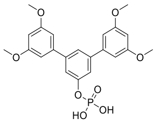	34.7 ± 4.5	73.4 ± 5.0	-
**18**	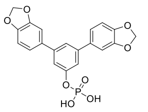	28.4 ± 4.4	56.4 ± 1.0	129 ± 5
**19**	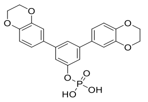	22.5 ± 3.8	71.2 ± 5.7	104 ± 4
**20**	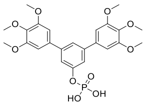	22.2 ± 3.6	-	-
**21**	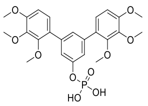	111 ± 10	-	-
**22**	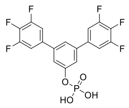	21.9 ± 2.1	46.9 ± 0.9	102 ± 9
**23**	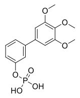	106 ± 4	-	-

Table S2: IC_{50}-values of test compounds against STAT5a, STAT5b, and STAT3.

Table S3

Protein	Compound **20**<br>IC_{50} (µM) or inhibition (%) at 200 µM	Compound **20**<br>K_i (µM)
STAT5a wild-type	22.2 ± 3.6 µM	10.9 ± 1.8 µM
STAT5b wild-type	37 ± 5 % inhibition	-
STAT5a Trp566Arg	155 ± 15 µM	74.0 ± 7.0 µM
STAT5b Arg566Trp	43 ± 7 % inhibition	-
STAT5a Trp566Arg/Lys644Met	44 ± 4 %	-
STAT5b Arg566Trp/Met644Lys	128 ± 8 µM	63.1 ± 4.2 µM
STAT5b-3M	101 ± 11 µM	49.0 ± 5.4 µM
STAT5b-6M	46 ± 8 % inhibition	-
STAT5b-7M	42.6 ± 8.8 µM	21.1 ± 4.4 µM

Table S3: Activities of compound **20** against STAT5 proteins in fluorescence polarization assays.

Library generation and virtual screening

A collection of 10,369,180 molecules classified as "commercially available" was downloaded from the ZINC database.[6] The library was filtered for fragments defined in the Structural Classification of Natural Products (SCONP) tree.[1] All cycles (including monocycles) were admitted as search terms, with the exception of the phenyl ring. The resulting 799,335 compounds were filtered for those that contain a monohydroxyphenyl moiety (to be used for subsequent virtual *O*-phosphorylation), whilst excluding compounds with more than one hydroxyl groups on the same aromatic ring as well as aliphatic alcohols to facilitate chemoselective *O*-phosphorylation at a later stage of the project. In addition, one of the *ortho* positions to the hydroxyl group was required to be unsubstituted to facilitate binding to SH2 domains. Molecules containing the following reactive functionalities are excluded: aldehydes, carbohydrates, strained lactams, acid chlorides, anhydrides, epoxides, and peroxides. After defining the maximum molecular weight of the selected compounds as 500 g/Mol, 85,021 compounds were chosen for virtual *O*-phosphorylation via conversion of compound structure to the SMILES (Simplified Molecular-Input Line-Entry System) format.[7] Using the "find and replace" function in Microsoft Excel, the hydroxy phenyl group (SMILES format: OC1=CC=CC=C1) was converted to the phenyl phosphate group (SMILES format: OC1=CC=CC=C1(P([O-])([O-])=O)). The protonation state of the molecules was adjusted according to their pK_a/pK_b values. The virtually phosphorylated molecules in the .sdf file format were separated into individual mol2 files using OpenBabel.[8] Mol2 files were converted to

pdbqt files using Raccoon.[9] The crystal structure of the phosphorylated, DNA-bound STAT3 dimer was downloaded from the protein data bank (PDB: 1bg1).[2] Water molecules, the DNA, and one of the protein units were deleted from the pdb file. Using AutoDock Tools,[10] polar hydrogens were added, partial charges were computed, and AutoDock atom types were assigned. Screening of 85,021 virtually *O*-phosphorylated compounds was carried out using AutoDock Vina.[11] Post-screening selection was first carried out by applying the following distance criteria: the maximum distance between the phosphorous atom and STAT3 Arg609 Cζ was defined as 4.8 Å; the maximum distance between one of its phosphate oxygens and STAT3 Arg609 Nη1 / Nη2 was defined as 2.9 Å, and the maximum distance between one of its phosphate oxygens and STAT3 Lys591 Nζ was defined as 4 Å. These criteria were to be fulfilled by at least 3 out of 5 docking poses. These distance criteria are also fulfilled by the SH2 domain-bound phosphotyrosine residue of the phosphorylated STAT3 dimer (Figure S3).[2] Visual inspection selected for molecules that were predicted to bind to the same region as the phosphotyrosine-containing peptide from the other STAT3 subunit, and had reasonable putative interactions with the protein.

Recombinant proteins

Expression and purification of STAT1, STAT3, STAT4, STAT5a, STAT5b, STAT5a Trp566Arg, STAT5b Arg566Trp, STAT5b 6M, STAT5b 7M, STAT6, and Lck have previously been described.[4, 12] STAT proteins were purified over a single His-Bind resin (Millipore) column. Wild-type and mutant STAT5a/b proteins used for activity analysis of **20** were purified over two successive His-Bind resin columns.[4, 13]

Fluorescence Polarization assays

Competitive binding assays based on fluorescence polarization were essentially carried out as described.[14] Unless stated otherwise, the following final concentration of proteins were used: 130 nM for STAT5a; 100 nM for STAT5b; 130 nM for STAT1; 125 nM for STAT3; 45 nM for STAT4; 105 nM for STAT6; 30 nM for Lck. Final concentration of all peptides: 10 nM. Peptides used: 5-carboxyfluorescein-GpYLVLDKW for STAT5a/b and point mutants thereof; 5-carboxyfluorescein-GpYLPQTV-NH_2 for STAT3; 5-carboxyfluorescein-GpYDKPHVL for STAT1; 5-carboxyfluorescein-GpYLPQNID-OH for STAT4; 5-carboxyfluorescein-GpYVPWQDLI-OH for STAT6; 5-carboxyfluorescein-GpYEEIP for Lck SH2. For the activity analysis of **20** against wild-type (wt) STAT5a/b and STAT5a/b point mutants, the following concentrations of double-purified protein were used, which reflect the approximate K_d values of the interactions with 5-carboxyfluorescein-GpYLVLDKW: STAT5a wt: 147 nM; STAT5b wt: 82 nM; STAT5b Arg566Trp: 239 nM; STAT5a Trp566Arg: 35nM; STAT5a Trp566Arg/Lys644Met: 30 nM; STAT5b Arg566Trp/Met644Lys: 80 nM; STAT5b 3M: 53 nM;

STAT5b 6M: 80 nM; STAT5b 7M: 282 nM. Assay buffer: 10 mM Tris (pH 8.0), 50 mM NaCl, 1 mM EDTA, 1 mM DTT, 0.1 % Nonidet P-40 substitute, and 2 % DMSO in water. A major part of the pipetting was done using a Biomek FXp sceening robot (Beckman-Coulter). Protein and test compounds were incubated for 1 h, followed by addition of the fluorescein-labeled peptide. Fluorescence polarization was measured after another hour using an Infinite F500 plate reader (Tecan). IC_{50} values were converted to K_i-values using the published equation.[15]

Cell culture

Chronic myeloid leukemia K562 cells were obtained from DSMZ (Braunschweig, Germany). K562 cells were cultured in RPMI 1640 medium (Gibco Life Technologies), supplemented by 10 % (v/v) FBS (Gibco Life Technologies), 2 mM L-glutamine (Gibco Life Technologies) and 1 % (v/v) penicillin/streptomycin (Gibco Life Technologies), at 37 °C, 5 % CO_2 and 95 % humidity.

Transfection and Western Blot

Transfection of cultured K562 cells and Western blotting was performed as previously described.[14, 16] In brief, cells were transfected with plasmid encoding either human wild-type STAT5a-GFP or STAT5b-GFP. K562 cells (6 x 10^5 cells per well) were seeded into 24-well plate (Corning) and were transfected using Fugene HD Transfection Reagent (Promega) with a 4:1 ratio of Fugene:DNA. 24 h later, the cells were treated with compound **27** at the indicated concentrations or DMSO for 4 h (final DMSO concentration: 0.2%). After harvesting, cells were centrifuged at 3.000 rpm at 4 °C for 5 min. Subsequently, cells were washed twice with cold phosphate buffered saline (PBS) and centrifuged again. Afterwards, cells were lysed with lysis buffer (lysis buffer composition: 50 mM Tris-HCl pH 7.5, 150 mM NaCl, 10 mM $Na_4P_2O_7$, 10 % glycerol, 1 % Triton X-100, 1 mM EDTA; protease inhibitors 1 mM PMSF and 100 ng/ml aprotinin; phosphatase inhibitors 10 mM NaF and 1 mM Na_3VO_4 were added freshly prior to use), and centrifuged at 14,000 rpm at 4 °C for 20 min. The cell lysates (total protein amount of 30 - 40 µg) were separated on a 10 % polyacrylamide gel under denaturing conditions and then transferred to a nitrocellulose membrane. Primary antibodies (phospho-STAT5, STAT5, β-Actin; 1:1000, Cell Signaling) were detected using α-rabbit-HRP secondary antibody (1:3000, Dako) and ECL (Western Lightning Plus chemiluminescence reagent, Perkin-Elmer). Visualization was carried out using an ImageQuant digital imaging system (GE Healthcare) and ImageJ software (NIH) was used for quantitative analysis.[17]

General synthetic methods

Method 1: Suzuki coupling reaction for *meta*-terphenyl compounds

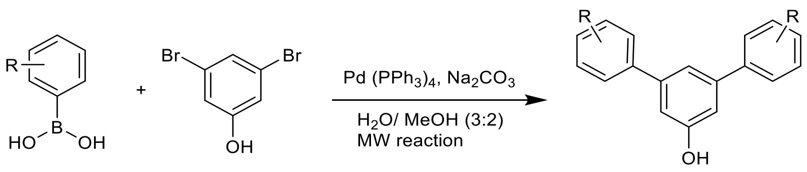

Dibromophenol (0.8 mmol), boronic acid (2.4 mmol), Na_2CO_3 (2.4 mmol), and $Pd(PPh_3)_4$ (48 µmol) were transferred to a 10 mL microwave reactor tube. Then the tube was flushed with argon. A degassed mixture of H_2O/MeOH (3:2 mL) was added under argon. The mixture was stirred at 110°C for 30 min at 300 Watt under microwave irradiation. After cooling to room temperature, 10 mL of water were added, and the reaction mixture was repeatedly extracted with ethyl acetate. The combined organic layers were dried over Na_2SO_4. Volatiles were removed under reduced pressure and purified by flash column chromatography.

Method 2: Aldol condensation

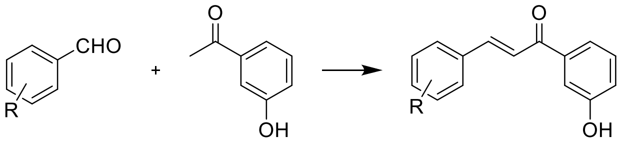

To a solution of 3-hydroxyacetophenone (1.5 mmol) and aldehyde (1.5 mmol) in 8 mL of acetic acid at 0° C, 0.8 mL of concentrated sulfuric acid was added slowly. Subsequently, the reaction mixture was stirred at room temperature for 2 days. After the completion of reaction (TLC control), the reaction mixture was quenched by addition of water, and mixture was repeatedly extracted with ethyl acetate. The combined organic layers were washed with water, dried over sodium sulfate and concentrated under reduced pressure. The crude material was purified by flash column chromatography.

Method 3: Synthesis of dibenzylphosphate esters

The aromatic alcohol (0.5 mmol) was dissolved in dry acetonitrile (5 mL). At to 0° C, CCl_4 (5 mmol), diisopropyl ethylamine (DIPEA) (1 mmol), and catalytic amounts of 4-(dimethylamino)pyridine (DMAP) were added subsequently. Dibenzyl phosphite (DBP) (0.75 mmol) was added dropwise, and the mixture was stirred at the same temperature for 0.5-1 h. Upon completion of the reaction, 1.5 mL of 0.5M KH_2PO_4 were added. The reaction mixture was repeatedly extracted with ethyl acetate, and the combined organic phases were dried over Na_2SO_4. The volatiles were removed under reduced pressure and purified by flash column chromatography.

Method 4: Debenzylation by hydrogenolysis

To a solution of dibenzylphosphate ester (0.5 mmol) in absolute ethanol (10 mL) under argon, 10% Pd/C (10-25 mg) was added. The argon atmosphere was exchanged for a hydrogen atmosphere. After completion of the reaction (usually 0.5-1.5 h, reversed-phase TLC control), the mixture was filtered through celite and washed with ethanol. After removal of the solvent under reduced pressure, the product was dissolved in water and washed with dichloromethane (DCM, 2 x 5 mL). The product was isolated by lyophilization of the aqueous phase. The phosphate monoesters can optionally be converted to their disodium salts by slowly adding 2 equivalents of $NaHCO_3$ (dissolved in 1-2 mL water) to the aqueous solution of debenzylated product, followed by lyophilization.

Method 5: Debenzylation by TMSBr

To a solution of dibenzylphosphate ester (0.5 mmol) in acetonitrile (4-8 mL) stirred under argon at 0° C, trimethylsilylbromide (TMSBr, 2.5 mmol) was added. After stirring for 2 h at 0° C, the reaction mixture was allowed to warm to room temperature and was stirred overnight. The reaction mixture was quenched with methanol and concentrated under reduced pressure. This process was repeated twice. The reaction product was dissolved in 2 mL of water, and the product was isolated by lyophilization.

Synthesis and spectroscopic characterization of compounds

Dibenzyl (4-chloro-4''-methoxy-[1,1':3',1''-terphenyl]-5'-yl) phosphate (**1a**)

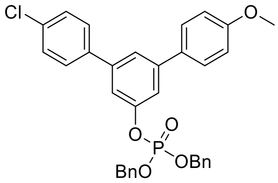

1a was obtained from 4-chloro-4''-methoxy-[1,1':3',1''-terphenyl]-5'-ol (50 mg, 0.16 mmol, purchased from Key Organics, ID: 1W-0328) according to Method 3. The crude product was purified by column chromatography (hexane/ethyl acetate 9:1 → 4:1), yielding **1a** as colorless oil (71 mg, 77 %). R_f = 0.25 (hexane/ethyl acetate 4:1). ^{1}H NMR (400 MHz, $CDCl_3$) δ = 3.87 (s, 3H), 5.14 – 5.23 (m, 4H), 6.96 - 6.99 (m, 2H), 7.22 (dt, *J*=2.4 Hz, 1.4 Hz, 1H), 7.28 – 7.30 (m, 1H), 7.32 - 7.39 (m, 11H), 7.40 - 7.44 (m, 3H), 7.45 -7.48 (m, 2H), 7.50 – 7.52 (m, 1H) ppm; ^{13}C NMR (101 MHz, $CDCl_3$) δ = 55.48, 70.20 (d, *J*=5.8 Hz), 114.38, 116.96 (d, *J*=4.6 Hz), 117.63 (d, *J*=4.8 Hz), 122.20, 128.22, 128.37, 128.56, 128.73, 128.81, 129.04, 132.42, 133.99, 135.52 (d, *J*=6.7 Hz), 138.67, 142.08, 143.24, 151.43 (d, *J*=7.0 Hz), 159.74 ppm; ^{31}P NMR (162 MHz, $CDCl_3$) δ = -5.0 (s, 1P) ppm; UV/Vis: λ (nm) = 262, 206; IR (KBr): $\tilde{\nu}$ = 3902, 3853, 3839, 3750, 3734, 3675, 3649, 3628, 3567, 2955, 2919, 2849, 2359, 2322, 1733, 1716, 1684, 1653, 1647, 1607, 1589, 1576, 1558, 1541, 1516, 1496, 1472, 1456, 1438, 1386, 1337, 1288,

1252, 1215, 1175, 1092, 1034, 1011, 1000, 964, 880, 855, 826, 787, 738, 696, 599, 499, 487, 472, 458 cm^{-1}; HRMS (ESI) found: [M+Na^+] m/z = 593.1253, calcd. for $C_{33}H_{28}ClNaO_5P^+$: 593.1255.

Sodium 4-chloro-4''-methoxy-[1,1':3',1''-terphenyl]-5'-yl phosphate (**1**)

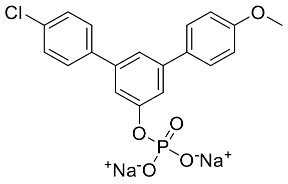

1 was obtained from **1a** (65 mg, 0.11 mmol) according to Method 4. After lyophilization, the product was obtained as a fluffy white solid (50 mg, quant. yield). Melting point: > 400 °C; ^{1}H NMR (300 MHz, D_2O) δ = 3.85 (s, 3H), 7.03 (d, *J*=8.9 Hz, 2H), 7.37 (s, 1H), 7.44 (d, *J*=8.6 Hz, 2H), 7.51 (s, 2H), 7.68 (t, *J*=9.0 Hz, 4H) ppm; ^{13}C NMR (75 MHz, CD_3OD) δ = 56.14, 115.24, 129.16, 129.51, 129.73, 134.05, 134.65, 140.74, 142.01, 143.01, 156.80 ppm; ^{31}P NMR (162 MHz, CD_3OD) δ = 1.4 (s, 1P) ppm; UV/Vis: λ (nm) = 259; IR (KBr): $\tilde{\nu}$ = 3433, 2962, 2934, 2838, 1733, 1697, 1645, 1635, 1608, 1595, 1515, 1496, 1442, 1404, 1388, 1343, 1290, 1250, 1180, 1095, 1068, 1032, 1011, 1000, 988, 948, 879, 823, 792, 765, 699, 657, 630, 565, 541, 509, 499, 481, 458 cm^{-1}; HRMS (ESI) found: [M-2Na^++H^+] m/z = 389.0349, calcd. for $C_{19}H_{15}ClO_5P^-$: 389.0351.

1-(3-hydroxyphenyl)-3-(perfluorophenyl)prop-2-en-1-one (**2a**)

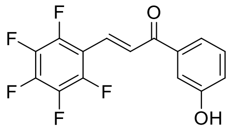

2a was obtained from 3-hydroxyacetophenone (200 mg, 1.47 mmol) and pentafluorobenzaldehyde (288 mg, 1.47 mmol) according to Method 2. The crude product was purified by column chromatography (hexane/ethyl acetate 4:1 → 13:7) to yield **2a** as pale yellow powder (210 mg, 49%). R_f = 0.65 (hexane/ethyl acetate 3:2). ^{1}H NMR (300 MHz, DMSO-d_6) δ = 7.09 (ddd, *J*=8.0 Hz, 2.6 Hz, 1.0 Hz, 1H), 7.37 - 7.42 (m, 2H), 7.52 (d, *J* = 16 Hz, 1 H), 7.53 – 7.55 (m, 1H), 7.83 (d, *J*=16.1 Hz, 1H), 9.90 (s, 1H) ppm; ^{19}F NMR (282 MHz, DMSO-d_6) δ = -162.90 – -162.69 (m, 2F), -152.41 (tt, *J*=22.2 Hz, 2.8 Hz, 1F), -140.48 – -140.35 (m, 2F) ppm.

Dibenzyl (3-(3-(perfluorophenyl)acryloyl)phenyl) phosphate (**2b**)

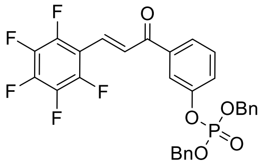

2b was obtained from **2a** (100 mg, 0.32 mmol) according to Method 3. The crude product was purified by column chromatography (hexane/ethyl acetate 4:1 → 3:2), providing **2b** as an oil (160 mg, 88%). R_f = 0.55 (hexane/ethyl acetate 4:1). ^{1}H NMR (300 MHz, $CDCl_3$) δ = 5.15 (d, *J*=8.8 Hz, 4H), 7.31 – 7.44 (m, 12H), 7.71 – 7.72 (m, 1H), 7.73 (d, *J*=2.3, 2H), 7.77 -7.83 (m, 1H) ppm; ^{13}C NMR (101 MHz, $CDCl_3$) δ = 70.25 (d, *J*=5.8 Hz), 110.09 – 110.48 (m), 120.21 (d, *J*=5.3 Hz), 125.11 (d, *J*=4.5 Hz), 125.18, 127.93, 128.11, 128.23, 128.49 – 128.63 (m), 128.62, 128.75, 130.20, 135.21 (d, *J*=6.4), 136.44 – 139.21 (m), 138.86, 140.39 – 143.44 (m), 144.44 – 147.31 (m) 150.94 (d, *J*=6.8 Hz), 188.14 ppm; ^{31}P NMR (162 MHz, $CDCl_3$) δ = -5.1 (s, 1P) ppm; HRMS (ESI) found: [M+Na$^+$] m/z = 597.0864, calcd. for $C_{29}H_{20}F_5NaO_5P^+$: 597.0861.

3-(3-(perfluorophenyl)acryloyl)phenyl dihydrogen phosphate (**2**)

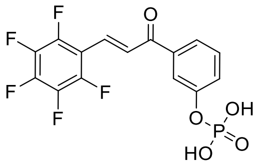

2 was obtained from **2b** (90 mg, 0.16 mmol) according to Method 5 as a yellow solid (60 mg, 97%). Melting point = 190 °C; ^{1}H NMR (400 MHz, CD_3OD) δ = 7.47 - 7.48 (m, 1H), 7.55 (t, *J*=8.1 Hz, 1H), 7.68 (d, *J*=16.1 Hz, 1H), 7.84 (m, 3H) ppm; ^{13}C NMR (101 MHz, CD_3OD) δ = 111.65, 121.22 (d, *J*=4.7 Hz), 125.85, 126.66 (d, *J*=4.7), 128.86, 129.82 – 130.11 (m), 131.33, 139.19 (d, *J*=245.4 Hz), 140.01, 143.17 (d, *J*=256.1 Hz), 147.27 (d, *J*=253.4 Hz), 153.35 (d, *J*=6.5 Hz), 189.82 ppm; ^{31}P NMR (162 MHz, CD_3OD) δ = -4.3 (s, 1P) ppm; UV/Vis: λ (nm) = 289, 251, 208; IR (KBr): $\tilde{\nu}$ = 3442, 2925, 2852, 2763, 2659, 2385, 2349, 2305, 1671, 1654, 1631, 1607, 1583, 1524, 1498, 1442, 1420, 1382, 1334, 1300, 1266, 1247, 1178, 1171, 1146, 1129, 1011, 976, 952, 909, 891, 864, 800, 762, 742, 706, 682, 613, 569, 547, 534, 504, 494, 482, 472, 443 cm^{-1}; HRMS (ESI) found: [M+Na$^+$] m/z = 416.9918, calcd. for $C_{15}H_8F_5NaO_5P^+$: 416.9922.

1-(3-hydroxyphenyl)-3-(2-(trifluoromethyl)phenyl)prop-2-en-1-one (**3a**)[18]

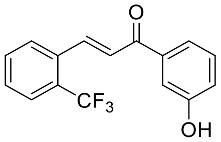

3a was obtained from 3-hydroxyacetophenone (272 mg, 2.00 mmol) and 2-(trifluoromethyl)benzaldehyde (348 mg, 2.00 mmol) according to Method 2. The crude product was purified by column chromatography (hexane/ethyl acetate 3:1 → 13:7), providing **3a** as a pale yellow powder (468 mg, 80 %). R_f = 0.23 (hexane/ethyl acetate 4:1). ^{1}H NMR (400 MHz, DMSO-d_6) δ = 7.07 (ddd, *J*=8.1 Hz, 2.5 Hz, 0.9 Hz, 1H), 7.37 (t, *J*=7.9 Hz, 1H), 7.46 (dd, *J*=2.5,

Hz, 1.6 Hz, 1H), 7.65 (ddt, J=7.7 Hz, 6.4 Hz, 1.1 Hz, 2H), 7.79 (m, 2H), 7.92 (q, J=1.5 Hz, 2H), 8.30 (d, J=7.9 Hz, 1H), 9.83 (s, 1H) ppm; ^{13}C NMR (101 MHz, DMSO) δ 114.71, 119.86, 120.77, 124.21 (q, J = 274.7 Hz), 126.20 (q, J = 5.6 Hz), 126.39, 127.51 (q, J = 29.6 Hz), 128.80, 129.97, 130.53, 132.84, 133.02, 137.66, 138.48, 157.82, 188.83; ^{19}F NMR (376 MHz, DMSO-d_6) δ = -57.72 (s, 3F) ppm; HRMS (ESI) found: [M+Na$^+$] m/z = 315.0604, calcd. for $C_{16}H_{11}F_3NaO_2^+$: 315.0603.

Dibenzyl (3-(3-(2-(trifluoromethyl)phenyl)acryloyl)phenyl) phosphate (**3b**)

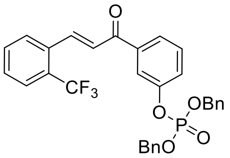

3b was obtained from **3a** (287 mg, 0.98 mmol) according to Method 3. The crude product was purified by column chromatography (hexane/ethyl acetate 4:1 → 3:2), yielding **3b** as a yellow oil (521 mg, 96 %). R_f = 0.13 (hexane/ethyl acetate 4:1); ^{1}H NMR (400 MHz, $CDCl_3$) δ 5.15 (dd, J = 8.7, 1.1 Hz, 4H) , 7.30 – 7.34 (m, 11H), 7.36 – 7.46 (m, 2H), 7.49 – 7.56 (m, 1H), 7.58 – 7.65 (m, 1H), 7.68 – 7.72 (m, 1H), 7.72 – 7.77 (m, 1H), 7.77 – 7.83 (m, 2H), 8.09 – 8.18 (m, 1H); ^{13}C NMR (101 MHz, $CDCl_3$) δ 70.37 (d, J = 5.9 Hz), 120.43 (d, J = 5.3 Hz), 122.72, 124.87 (d, J = 4.4 Hz), 125.43, 126.11, 126.46 (q, J = 5.5 Hz), 128.19, 128.26, 128.78, 128.91, 129.99, 130.20, 132.28, 133.98, 135.40 (d, J = 6.8 Hz), 139.43, 140.83, 150.98 (d, J = 6.8 Hz), 188.94; ^{19}F NMR (377 MHz, $CDCl_3$) δ -58.84; ^{31}P NMR (162 MHz, $CDCl_3$) δ -6.29; UV/Vis: λ (nm) = 285, 257, 204; IR (KBr): $\tilde{\nu}$ = 3442, 3088, 3068, 3037, 2957, 2900, 1670, 1614, 1599, 1581, 1487, 1457, 1433, 1386, 1333, 1316, 1276, 1240, 1219, 1180, 1160, 1128, 1082, 1061, 1040, 1027, 1004, 976, 964, 925, 907, 893, 861, 852, 811, 767, 749, 735, 699, 684, 669, 648, 607, 599, 583, 575, 541, 526, 498, 477, 463, 451 cm^{-1}; HRMS (ESI) found: [M+H$^+$] m/z = 553.1393, calcd. for $C_{30}H_{25}F_3O_5P^+$: 553.1386.

3-(3-(2-(trifluoromethyl)phenyl)acryloyl)phenyl dihydrogen phosphate (**3**)

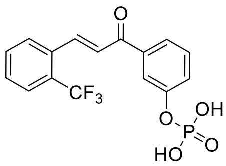

3 was obtained from **3b** (56 mg, 0.10 mmol) according to Method 5 as pale brown oily material (37 mg, 98 %, purity ~85 %). Melting point = 181 °C; ^{1}H NMR (400 MHz, CD_3OD) δ = 7.46 – 7.52 (m, 1H), 7.53 – 7.65 (m, 2H), 7.69 – 7.82 (m, 3H), 7.90 – 7.98 (m, 2H), 8.09 – 8.19 (m, 2H) ppm; ^{13}C NMR (101 MHz, MeOD) δ 119.84 – 119.99 (m), 124.63, 124.80, 124.91 – 125.17 (m), 125.59, 125.79 – 125.92 (m), 128.14, 129.86, 130.00, 132.41, 139.11, 139.61, 133.41, 151.89, 189.04; ^{19}F NMR (377 MHz, MeOD) δ -60.06; ^{31}P NMR (162 MHz, CD_3OD) δ = -5.4 (s,

1P) ppm; UV/Vis: λ (nm) = 293, 242, 207; IR (KBr): $\tilde{\nu}$ = 3624, 3605, 3594, 3585, 3565, 3427, 3418, 3078, 2746, 2727, 2384, 2352, 2317, 2306, 1731, 1724, 1713, 1681, 1667, 1608, 1583, 1485, 1440, 1362, 1332, 1315, 1288, 1250, 1111, 1045, 1007, 989, 946, 895, 860, 798, 766, 759, 742, 685, 664, 647, 595, 582, 523, 514, 504, 494, 480, 454, 442, 405 cm^{-1}; HRMS (ESI) found: [$M+Na^+$] m/z = 395.0265, calcd. for $C_{16}H_{12}F_3NaO_5P^+$: 395.0267.

N-(4-(3-(3-hydroxyphenyl)-3-oxoprop-1-en-1-yl)phenyl)acetamide (**4a**)

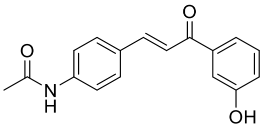

4a was obtained from 3-hydroxyacetophenone (200 mg, 1.47 mmol) and 4-acetamidobenzaldehyde (240 mg, 1.47 mmol) according to Method 2. The crude product was purified by column chromatography (hexane/ethyl acetate 4:1 → 3:2), yielding **4a** as yellow powder (355 mg, 86 %). R_f = 0.25 (hexane/ethyl acetate 3:2). ^{1}H NMR (300 MHz, DMSO-d_6) δ = 2.08 (s, 3H), 6.99 – 7.10 (m, 1H), 7.36 (t, *J*=7.9 Hz, 1H), 7.44 (t, *J*=2.0 Hz, 1H), 7.54 – 7.65 (m, 1H), 7.59 – 7.74 (m, 4H), 7.81 (d, *J*=8.6 Hz, 2H), 9.78 (s, 1H), 10.17 (s, 1H) ppm.

3-(3-(4-acetamidophenyl)acryloyl)phenyl dibenzyl phosphate (**4b**)

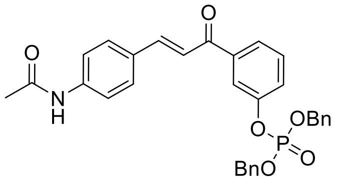

4b was obtained from **4a** (150 mg, 0.53 mmol) according to Method 3. The crude product was purified by column chromatography (DCM/MeOH 49:1), yielding **4b** as an oil (252 mg, 84 %). R_f = 0.40 (DCM/MeOH 19:1%); ^{1}H NMR (400 MHz, $CDCl_3$) δ = 2.09 (s, 3H), 5.09 (d, *J*=8.7 Hz, 4H), 7.19 (s, 1H), 7.22 – 7.28 (m, 11H), 7.34 (t, *J*=7.9 Hz, 1H), 7.44 (d, *J*=8.7 Hz, 2H), 7.51 (d, *J*=8.4 Hz, 2H), 7.65 (d, *J*=15.6 Hz, 2H), 7.71 (ddt, *J*=7.7 Hz, 1.9 Hz, 1.0 Hz, 1H), 7.94 (s, 1H) ppm; ^{31}P NMR (162 MHz, $CDCl_3$) δ = -5.3 (s, 1P) ppm; HRMS (ESI) found: [$M+Na^+$] m/z = 564.1548, calcd. for $C_{31}H_{28}NNaO_6P^+$: 564.1546.

3-(3-(4-acetamidophenyl)acryloyl)phenyl dihydrogen phosphate (**4**)

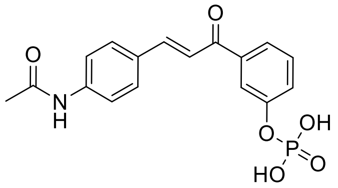

4 was obtained from **4b** (100 mg, 0.18 mmol) and TMSBr (0.12 mL, 0.89 mmol) according to Method 5 as pale brown solid (62 mg, 97 %). Melting point = 207-210 °C; ^{1}H NMR (400 MHz,

CD$_3$OD) δ = 2.14 (s, 3H), 7.43 – 7.49 (m, 1H), 7.50 - 7.56 (m, 1H), 7.60 – 7.80 (m, 6H), 7.87 - 7.91 (m, 2H) ppm; ^{13}C NMR (101 MHz, CD$_3$OD) δ = 23.97, 120.96, 121.20 (d, J = 4.7 Hz), 125.81, 126.00 (d, J = 4.8 Hz), 130.73, 131.14, 131.50, 131.66, 141.11, 142.58, 146.34, 153.22 (d, J = 6.4 Hz), 171.81, 191.29 ppm; ^{31}P NMR (162 MHz, CD$_3$OD) δ = -4.2 (s, 1P) ppm; UV/Vis: λ (nm) = 344, 247, 205; IR (KBr): $\tilde{\nu}$ = 3607, 3594, 3579, 3566, 3551, 3542, 3531, 3519, 3418, 3409, 3367, 3384, 3359, 3334, 3312, 3218, 3208, 3076, 2925, 2376, 2350, 2306, 1667, 1650, 1574, 1540, 1512, 1439, 1415, 1376, 1331, 1302, 1242, 1183, 1162, 1049, 1031, 1005, 964, 891, 833, 812, 796, 743, 645, 528, 520, 488 cm^{-1}; HRMS (ESI) found: [M-H$^+$] m/z = 360.0641, calcd. for formula $C_{17}H_{15}NO_6P^-$: 360.0642.

Dibenzyl (3-(4-nitrobenzoyl)benzofuran-5-yl) phosphate (**5a**)

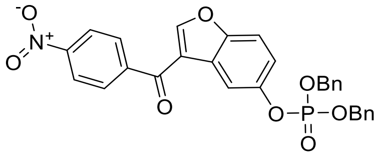

5a was obtained from (5-hydroxybenzofuran-3-yl)(4-nitrophenyl)methanone (purchased from Vitas M, ID: STL325066, 35 mg, 0.12 mmol) according to Method 3. The crude product was purified by column chromatography (hexane/ethyl acetate 4:1 → 13:7), yielding **5a** as colorless oil (40 mg, 60 %). R_f = 0.25 (hexane/ethyl acetate 7:3); ^{1}H NMR (400 MHz, CDCl$_3$) δ = 5.16 (d, *J*=8.5 Hz, 4H), 7.23 – 7.28 (m, 1H), 7.30 – 7.41 (m, 10H), 7.48 (d, *J*=9.0 Hz, 1H), 8.00 (d, *J*=8.8 Hz, 3H), 8.09 (s, 1H), 8.37 (d, *J*=8.8 Hz, 2H) ppm; ^{13}C NMR (CDCl$_3$) δ = 70.20 (d, *J*=5.8), 112.49, 114.20 (d, *J*=5.1 Hz), 119.29 (d, *J*=4.2 Hz), 121.22, 124.08, 125.59, 128.17, 128.69, 128.74, 129.63, 135.47 (d, *J*=6.7 Hz), 143.99, 148.01 (d, *J*=7.0 Hz), 150.08, 152.81, 153.81, 187.72 ppm; ^{31}P NMR (CDCl$_3$) δ = -4.7 (s, 1P) ppm; UV/Vis: λ (nm) = 257, 204; IR (KBr): $\tilde{\nu}$ = 3649, 3628, 3586, 3566, 3445, 3108, 3066, 1684, 1644, 1624, 1602, 1546, 1522, 1497, 1466, 1452, 1384, 1347, 1319, 1281, 1256, 1216, 1165, 1140, 1114, 1081, 1036, 1015, 1001, 964, 903, 868, 848, 810, 780, 746, 721, 711, 697, 618, 601, 518, 508, 499 cm^{-1}.

Sodium 3-(4-nitrobenzoyl)benzofuran-5-yl phosphate (**5**)

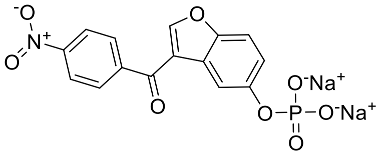

5 was obtained from **5a** (35 mg, 0.06 mmol) according to Method 4. After lyophilization, the product was obtained as an off-white solid (16 mg, 63%). Decomposition point = 305 °C. ^{1}H NMR (400 MHz, D$_2$O) δ = 6.89 (d, *J*=8.1 Hz, 2H), 7.33 (d, *J*=9.6 Hz, 1H), 7.59 (d, *J*=9.0 Hz, 1H), 7.76 (s, 1H), 7.84 (d, *J*=8.1 Hz, 2H), 8.28 (s, 1H) ppm; ^{31}P NMR (162 MHz, D$_2$O) δ = 1.6 (s, 1P) ppm; UV/Vis: λ (nm) = 333, 287, 241; IR (KBr): $\tilde{\nu}$ = 3425, 1696, 1662, 1634, 1599,

1542, 1516, 1464, 1447, 1385, 1268, 1240, 1169, 1126, 1107, 984, 948, 886, 836, 772, 705, 681, 657, 634, 615, 528, 503, 492, 479 cm^{-1}.

3-(2-(1H-indol-1-yl)acetamido)phenyl dibenzyl phosphate (**6a**)

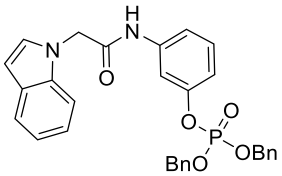

6a was obtained from *N*-(3-hydroxyphenyl)-2-(1*H*-indol-1-yl)acetamide (50 mg, 0.19 mmol) according to Method 3. After purification by column chromatography (hexane/ethyl acetate 4:1 → 3:2), **6a** was obtained as colorless oil (78 mg, 79 %). R_f = 0.25 (hexane/ethyl acetate 3:2); ^{1}H NMR (300 MHz, $CDCl_3$) δ = 4.77 (s, 2H), 5.08 (d, *J*=8.6 Hz, 4H), 6.61 (dd, *J*=3.2 Hz, 0.8 Hz, 1H), 6.82 - 6.86 (m, 1H), 6.98 - 7.10 (m, 3H), 7.12 - 7.34 (m, 13H), 7.66 (ddd, *J*=7.6 Hz, 1.4 Hz, 0.7 Hz, 1H), 7.72 (s, 1H) ppm; ^{13}C NMR (75 MHz, $CDCl_3$) δ = 50.30, 70.27 (d, *J*=5.9 Hz), 103.61, 109.31, 112.08 (d, *J*=5.3 Hz), 116.04 (d, *J*=4.6 Hz), 116.79, 120.56, 121.46, 122.78, 128.14, 128.54, 128.68, 128.78, 128.93, 129.91, 135.27 (d, *J*=6.7), 136.44, 138.49, 150.64 (d, *J*=6.7 Hz), 166.62 ppm; ^{31}P NMR (162 MHz, $CDCl_3$) δ = -5.5 (s, 1P) ppm; MS (ESI) found: [$M+Na^+$] m/z = 549.1, calcd. for $C_{30}H_{26}N_2Na_2O_5P^+$: 549.2.

Sodium 3-(2-(1H-indol-1-yl)acetamido)phenyl phosphate (**6**)

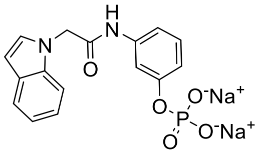

6 was obtained from **6a** (60 mg, 0.11 mmol) according to Method 4. The product was obtained as white solid (45 mg, quant. yield). Melting point = 162 °C; ^{1}H NMR (400 MHz, DMSO-d_6) δ = 5.03 (s, 2H), 6.45 (d, *J*=3.0 Hz, 1H), 6.85 (d, *J*=8.0 Hz, 1H), 7.02 (ddd, *J*=7.9 Hz, 7.0 Hz, 1.0 Hz, 1H), 7.12 (t, *J*=7.5 Hz, 1H), 7.21 (t, *J*=8.0 Hz, 1H), 7.37 (d, *J*=3.1 Hz, 1H), 7.41 (d, *J*=8.1 Hz, 2H), 7.46 (s, 1H), 7.55 (d, *J*=7.3 Hz, 1H), 10.54 (s, 1H) ppm; ^{13}C NMR (101 MHz, DMSO-d_6) δ = 49.13, 100.78, 109.74, 114.06, 115.18, 119.10, 120.31, 121.14, 128.12, 129.37, 129.96, 136.41, 139.62, 152.44, 166.38 ppm; ^{31}P NMR (162 MHz, DMSO-d_6) δ = 0.03 (s, 1P) ppm; UV/Vis: λ (nm) = 292, 281, 244, 209; IR (KBr): $\tilde{\nu}$ = 3444, 3268, 2926, 1670, 1604, 1543, 1516, 1486, 1465, 1444, 1423, 1356, 1322, 1281, 1259, 1226, 1186, 1150, 1091, 1010, 996, 959, 877, 866, 787, 744, 737, 717, 687, 579, 547, 524, 505, 454, 428 cm^{-1}; HRMS (ESI) found: [$M+2H^+-Na^+$] m/z = 369.0607, calcd. for $C_{16}H_{15}N_2NaO_5P^+$: 369.0611.

Dibenzyl (3-(3-(pyrrolidin-1-ylsulfonyl)benzamido)phenyl) phosphate (**7a**)

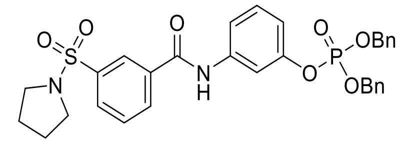

7a was obtained from *N*-(3-hydroxyphenyl)-3-(pyrrolidin-1-ylsulfonyl)benzamide (purchased from Asinex, ID: BAS 00822902, 30 mg, 0.09 mmol) according to Method 3. The crude product was purified by column chromatography (DCM/acetone 49:1 → 19:1) yielding as colorless oil (35 mg, 67 %). R_f = 0.33 (DCM/acetone 9:1); ^{1}H NMR (400 MHz, $CDCl_3$) δ = 1.73 – 1.78 (m, 4H), 3.22 – 3.37 (m, 4H), 5.11 (d, *J*=8.4 Hz, 4H), 6.90 (d, *J*=8.2 Hz, 1H), 7.23 (t, *J*=8.1 Hz, 1H), 7.28 – 7.39 (m, 10H), 7.55 (d, *J*=8.5 Hz, 2H), 7.60 (t, *J*=7.8 Hz, 1H), 7.95 (d, *J*=7.9 Hz, 1H), 8.14 (d, *J*=7.8 Hz, 1H), 8.31 (s, 1H), 8.66 (s, 1H) ppm; ^{13}C NMR (101 MHz, $CDCl_3$) δ = 25.40, 48.18, 70.25 (d, *J*=6.0 Hz), 112.56 (d, *J*=5.2 Hz), 116.17 (d, *J*=4.7 Hz), 117.37, 126.06, 128.19, 128.76 (d, *J*=7.2 Hz), 129.72, 130.08, 130.38, 131.89, 135.42 (d, *J*=6.7 Hz), 136.12, 137.84, 139.27, 150.79 (d, *J*=6.8 Hz), 164.51 ppm; ^{31}P NMR (162 MHz, $CDCl_3$) δ = -5.5 (s, 1P) ppm; HRMS (ESI) found: [M+Na$^+$] m/z = 629.1480, calcd. for $C_{31}H_{31}N_2NaO_7PS^+$: 629.1482.

Sodium 3-(3-(pyrrolidin-1-ylsulfonyl)benzamido)phenyl phosphate (**7**)

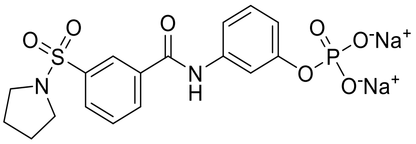

7 was obtained from **7a** (30 mg, 0.04 mmol) according to Method 4. The product was obtained as an off-white solid (19 mg, 97 %). Decomposition point > 300 °C; ^{1}H NMR (400 MHz, D_2O) δ = 1.72 – 1.81 (m, 4H), 3.26 – 3.36 (m, 4H), 7.15 (d, *J*=7.9 Hz, 1H), 7.30 (s, 1H), 7.33 – 7.47 (m, 2H), 7.83 (t, *J*=7.9 Hz, 1H), 8.11 (d, *J*=7.9 Hz, 1H), 8.22 (d, *J*=7.8 Hz, 1H), 8.34 (s, 1H) ppm; ^{13}C NMR (101 MHz, D_2O) δ = 25.03, 48.57, 114.92, 117.00, 118.33, 126.58, 129.89, 130.35, 130.80, 132.53, 135.73 (d, *J*=15.4 Hz), 137.51, 154.58 (d, *J*=6.2 Hz), 162.60, 167.95 pmm; ^{31}P NMR (162 MHz, D_2O) δ = 1.2 (s, 1P) ppm; UV/Vis: λ (nm) $\tilde{\nu}$ = 328, 263; IR (KBr): = 3423, 3069, 2972, 2954, 2874, 2763, 2538, 1926, 1696, 1667, 1608, 1574, 1542, 1486, 1442, 1415, 1333, 1272, 1183, 1159, 1122, 1003, 991, 975, 909, 879, 833, 779, 742, 689, 666, 608, 591, 567, 499, 469, 459, 410 cm^{-1}; HRMS (ESI) found: [M+H$^+$] m/z = 471.0364, calcd. for $C_{17}H_{18}N_2Na_2O_7PS^+$: 471.0362.

Dibenzyl (4-(4-(3-((4-cyclohexylphenyl)sulfonamido)propanoyl)piperazin-1-yl)phenyl) phosphate (**8a**)

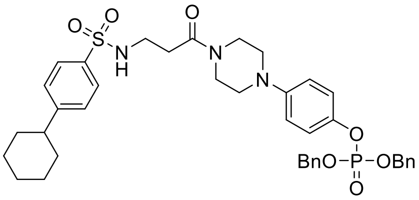

8a was obtained from 4-cyclohexyl-N-(3-(4-(4-hydroxyphenyl)piperazin-1-yl)-3-oxopropyl)benzenesulfonamide (purchased from Enamine, ID: Z31728852, 50 mg, 0.11 mmol) according to Method 3. The crude product was purified by column chromatography (DCM/MeOH 99:1 → 49:1), providing **8a** as a colorless oil (61 mg, 79 %). R_f = 0.75 (DCM/MeOH 19:1); ^{1}H NMR (400 MHz, $CDCl_3$) δ = 1.16 – 1.32 (m, 1H), 1.31 – 1.48 (m, 4H), 1.65 – 1.80 (m, 2H), 1.85 (d, *J*=7.0 Hz, 4H), 2.58 (t, *J*=5.6 Hz, 2H), 3.05 (dd, *J*=6.8 Hz, 3.3 Hz, 4H), 3.24 (q, *J*=6.3 Hz, 2H), 3.47 – 3.55 (m, 2H), 3.65 – 3.75 (m, 2H), 5.11 (d, *J*=8.2 Hz, 4H), 5.56 (t, *J*=6.6 Hz, 1H), 6.81 (d, *J*=8.9 Hz, 2H), 7.06 (dd, *J*=9.1 Hz, 1.2, 2H), 7.27 – 7.40 (m, 11H), 7.77 (d, *J*=8.5 Hz, 2H) ppm; ^{13}C NMR (101 MHz, $CDCl_3$) δ = 26.07, 26.78, 33.11, 34.25, 39.30, 41.58, 44.65, 45.25, 49.98, 50.22, 70.00 (d, *J*=5.9 Hz), 118.13, 120.89 (d, *J*=4.8 Hz), 127.14, 127.72, 128.09, 128.69, 128.72, 135.63 (d, *J*=7.0 Hz), 137.55, 144.61 (d, *J*=7.0 Hz), 148.38 (d, *J*=1.3 Hz), 153.27, 169.61 ppm; ^{31}P NMR (162 MHz, $CDCl_3$) δ = -4.6 (s, 1P) ppm; UV/Vis: λ (nm) = 287, 248, 230, 202; IR (KBr): $\tilde{\nu}$ = 3854, 3751, 3745, 3735, 3676, 3649, 3567, 2364, 2345, 1734, 1716, 1699, 1684, 1670, 1653, 1647, 1636, 1558, 1541, 1521, 1508, 1473, 1521, 1508, 1473, 1457, 599, 499 cm^{-1}; HRMS (ESI) found: [$M+Na^+$] m/z = 754.2687, calcd. for $C_{39}H_{46}N_3NaO_7PS^+$: 754.2686.

Sodium 4-(4-(3-((4-cyclohexylphenyl)sulfonamido)propanoyl)piperazin-1-yl)phenyl phosphate (**8**)

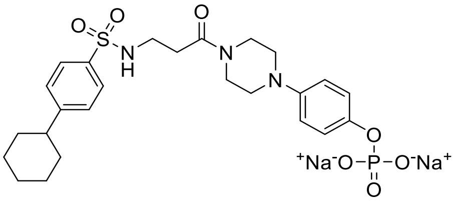

8 was obtained from **8a** (55 mg, 0.08 mmol) according to Method 4. After lyophilization, the product was obtained as a fluffy white sodium salt (45 mg, quant. yield, purity ~86%). Melting point: > 300 °C; ^{1}H NMR (300 MHz, D_2O) δ = 1.2 – 1.9 (m, 10H), 2.52 – 2.67 (m, 3H), 2.95 – 3.07 (m, 4H), 3.12 – 3.22 (m, 2H), 3.53 – 3.74 (m, 4H), 6.94 (d, *J*=9.0 Hz, 2H), 7.22 (d, *J*=9.1, 2H), 7.44 (d, *J*=8.4 Hz, 2H), 7.78 (d, *J*=8.4 Hz, 2H) ppm; ^{13}C NMR (75 MHz, CD_3OD) δ = 26.91, 27.63, 33.98, 35.18, 40.30, 42.74, 45.66, 46.61, 52.28 (d, *J*=30.7), 119.76, 121.88 (d, *J*=4.7), 128.04, 128.79, 138.67, 146.64, 151.12 (d, *J*=6.1), 154.73, 171.65 ppm; ^{31}P NMR (162 MHz,

CD_3OD) δ = 1.9 (s, 1P) ppm; UV/Vis: λ (nm) = 283, 232; IR (KBr): $\tilde{\nu}$ = 3434, 2925, 2851, 2548, 1923, 1732, 1697, 1667, 1625, 1558, 1509, 1472, 1450, 1401, 1362, 1237, 1157, 1097, 1032, 984, 892, 835, 765, 707, 594, 573, 533 cm^{-1}; HRMS (ESI) found: [M - $2Na^+$ + H^+] m/z = 550.1774, calcd. for formula $C_{25}H_{33}N_3O_7PS^-$ = 550.1782.

Dibenzyl (4-(4-(3-(N-cyclohexyl-N-methylsulfamoyl)benzoyl)piperazin-1-yl)phenyl) phosphate (**9a**)

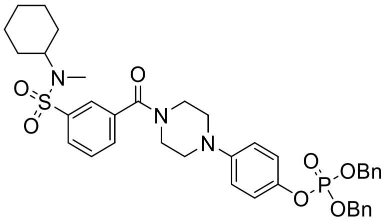

9a was obtained from *N*-cyclohexyl-3-(4-(4-hydroxyphenyl)piperazine-1-carbonyl)-*N*-methylbenzenesulfonamide (purchased from UORSY, ID: PB28346215, 50 mg, 0.11 mmol) according to Method 3. The crude product was purified by column chromatography (DCM/acetone 49:1 → 19:1), providing **9a** as a colorless oil (55 mg, 70 %). R_f = 0.33 (DCM/acetone 9:1). ^{1}H NMR (400 MHz, $CDCl_3$) δ = 0.95 – 1.82 (m, 11H), 2.76 (s, 3H), 2.98 – 3.27 (m, 4H), 3.33 – 4.06 (m, 4H), 5.11 (d, *J*=8.2 Hz, 4H), 6.83 (d, *J*=9.1 Hz, 2H), 7.07 (d, *J*=7.9 Hz, 2H), 7.28 – 7.39 (m, 10H), 7.55 – 7.69 (m, 2H), 7.85 – 7.93 (m, 2H) ppm; ^{13}C NMR (75 MHz, $CDCl_3$) δ = 25.36, 25.84, 28.83, 30.51, 42.37, 47.75, 50.36, 57.16, 69.98 (d, *J*=5.8 Hz), 118.22, 118.23, 120.90 (d, *J*=4.7 Hz), 125.58, 128.08, 128.14, 128.67, 128.70, 129.71, 131.00, 135.62 (d, *J*=6.9 Hz), 136.70, 141.22, 144.66 (d, *J*=7.3 Hz), 148.34 (d, *J*=1.3 Hz), 168.69 ppm; ^{31}P NMR (162 MHz, $CDCl_3$) δ = -4.6 (s, 1P) ppm; UV/Vis: λ (nm) = 248, 205; IR (KBr): $\tilde{\nu}$ = 3901, 3870, 3853, 3838, 3819, 3801, 3749, 3734, 3724, 3710, 3701, 3688, 3669, 3648, 3627, 3566, 3446, 3065, 3034, 2931, 2856, 1716, 1698, 1683, 1636, 1508, 1456, 1438, 1386, 1338, 1284, 1233, 1214, 1175, 1147, 1020, 1009, 957, 891, 807, 740, 697, 590, 507 cm^{-1}; HRMS (ESI) found: [M+Na^+] m/z = 740.2523, calcd. for $C_{38}H_{44}N_3NaO_7PS^+$: 740.2530.

Sodium 4-(4-(3-(N-cyclohexyl-N-methylsulfamoyl)benzoyl)piperazin-1-yl)phenyl phosphate (**9**)

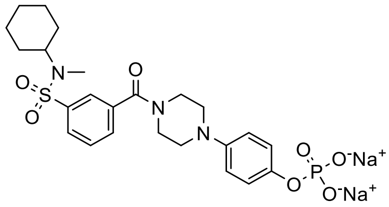

9 was obtained from **9a** (30 mg, 0.04 mmol) according to Method 4. After lyophilization, the product was obtained as off-white solid (24 mg, quant. yield). Decomposition point: 220 °C. ^{1}H NMR (400 MHz, D_2O) δ = 1.15 – 1.80 (m, 10H), 2.82 (s, 3H), 3.03 – 3.19 (m, 2H), 3.21 – 3.35 (m, 2H), 3.53 - 3.65 (m, 2H), 3.65 – 3.77 (m, 1H), 3.89 – 4.03 (m, 2H), 7.09 (d, *J*=8.7 Hz, 2H),

7.20 (d, J=8.5 Hz, 2H), 7.73 – 7.84 (m, 2H), 7.95 (s, 1H), 7.99 - 8.09 (m, 1H) ppm; ^{13}C NMR (101 MHz, D_2O) δ = 24.85, 25.47, 28.72, 29.80, 42.29, 47.61, 50.70, 51.27, 57.80, 119.53, 121.32 (d, J=4.3 Hz), 124.84, 128.63, 130.65, 131.56, 135.96, 139.16, 145.37, 149.42 (d, J=6.4 Hz), 160.53, 170.50 ppm; ^{31}P NMR (162 MHz, D_2O) δ = 1.4 (s, 1P) ppm; UV/Vis: λ (nm) = 235; IR (KBr): $\tilde{\nu}$ = 3427, 2931, 2857, 2820, 2658, 2552, 1924, 1697, 1635, 1575, 1509, 1480, 1447, 1412, 1337, 1288, 1243, 1227, 1147, 1108, 1047, 1021, 988, 958, 937, 890, 848, 834, 808, 758, 702, 630, 588, 562, 531, 502, 492, 480, 472, 452, 441 cm^{-1}; HRMS (ESI) found: [M+H$^+$] m/z = 582.1405, calcd. for formula $C_{24}H_{31}N_3Na_2O_7PS^+$ = 582.1410.

4,4''-dichloro-[1,1':3',1''-terphenyl]-5'-ol (**10a**)

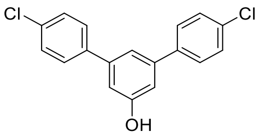

10a was obtained from 3,5-dibromophenol (150 mg, 0.60 mmol) and 4-chlorophenylboronic acid (289 mg, 1.79 mmol) according to Method 1. The crude product was purified by column chromatography in hexane/ethyl acetate (19:1→ 9:1) to afford pure white liquid (169 mg, 90 %). R_f = 0.45 (hexane/ethyl acetate 4:1); Melting point = 161 °C (Lit:[19] 160-161 °); ^{1}H NMR (400 MHz, $CDCl_3$) δ = 7.01 (d, J=1.5 Hz, 2H), 7.29 (t, J=1.5 Hz, 1H), 7.40 – 7.43 (m, 4H), 7.51 – 7.55 (m, 4H) ppm; ^{13}C NMR (75 MHz, $CDCl_3$) δ = 113.34, 118.69, 128.54, 129.12, 133.98, 139.12, 142.48, 156.45 ppm; UV/Vis: λ (nm) = 300, 253, 206; IR (KBr) $\tilde{\nu}$ = 3648, 3627, 3420, 2952, 2925, 2853, 1901, 1698, 1684, 1635, 1613, 1597, 1568, 1495, 1474, 1439, 1401, 1384, 1336, 1262, 1189, 1092, 1060, 1013, 998, 917, 871, 861, 821, 720, 697, 687, 639, 607, 569, 543, 504, 478; HRMS (ESI) found: [M-H$^+$] m/z = 313.0190, calcd. for $C_{18}H_{11}NaCl_2O^-$: 313.0192.

Dibenzyl (4,4''-dichloro-[1,1':3',1''-terphenyl]-5'-yl) phosphate (**10b**)

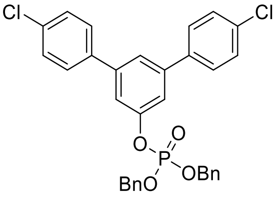

10b was obtained from **10a** (150 mg, 0.48 mmol) according to Method 3. The crude product was purified by column chromatography (hexane/ethyl acetate 19:1 → 17:3), providing **10b** as colorless oil (173 mg, 63 %). R_f = 0.35 (hexane/ethyl acetate 4:1); ^{1}H NMR (400 MHz, $CDCl_3$) δ = 5.18 - 5.28 (m, 4H), 7.31 (s, 4H), 7.36 - 7.48 (m, 16H), 7.53 (s, 1H) ppm; ^{13}C NMR (101 MHz, $CDCl_3$) δ = 70.20 (d, J=5.8 Hz), 117.88 (d, J=4.7 Hz), 122.35, 128.19, 128.48, 128.69, 128.80, 129.05, 134.12, 135.41 (d, J=6.5 Hz), 138.28, 142.27, 151.43 (d, J=6.9 Hz) ppm; ^{31}P NMR (162 MHz, $CDCl_3$) δ = -6.1 (s, 1P) ppm; UV/Vis: λ (nm) = 257, 207; IR (KBr): $\tilde{\nu}$ = 3445, 3089, 3065, 3033, 2957, 2895, 1901, 1606, 1589, 1567, 1496, 1455, 1438, 1384, 1335, 1283,

1215, 1174, 1092, 1036, 1013, 1002, 966, 920, 882, 854, 823, 786, 739, 719, 697, 638, 620, 599, 554, 505, 476, 459; HRMS (ESI) found: $[M+Na^+]$ m/z = 597.0761, calcd. for $C_{32}H_{25}Cl_2NaO_4P^+$: 597.0760.

4,4''-dichloro-[1,1':3',1''-terphenyl]-5'-yl dihydrogen phosphate (**10**)

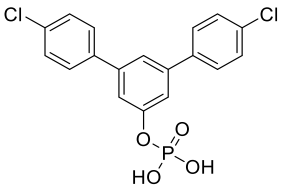

10 was obtained from **10b** (160 mg, 0.28 mmol) according to Method 4. After lyophilization, the product was obtained as a fluffy white solid (107 mg, 97 %). ^1H NMR (400 MHz, CD_3OD) δ = 7.42 – 7.50 (m, 6H), 7.61 (s, 1H), 7.64 – 7.71 (m, 4H) ppm; ^{13}C NMR (101 MHz, CD_3OD) δ = 119.07, 122.37, 129.67, 130.05, 134.98, 140.11, 143.31 ppm; ^{31}P NMR (162 MHz, CD_3OD) δ = -5.0 (s, 1P) ppm; UV/Vis: λ (nm) = 255, 213; IR (KBr): $\tilde{\nu}$ = 3434, 3055, 3033, 2925, 2853, 2321, 1900, 1771, 1749, 1683, 1635, 1607, 1590, 1567, 1496, 1453, 1438, 1422, 1384, 1335, 1175, 1093, 1064, 1012, 1006, 983, 878, 823, 764, 718, 697, 637, 626, 607, 553, 505, 477, 436; HRMS (ESI) found: $[M-H^+]$ m/z = 392.9848, calcd. for $C_{18}H_{12}Cl_2O_4P^-$: 392.9856.

[1,1':3',1''-terphenyl]-5'-ol (**11a**)

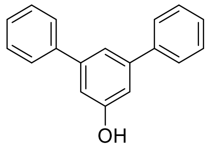

11a was obtained from 3,5-dibromophenol (50 mg, 0.20 mmol) and phenylboronic acid (73 mg, 0.60 mmol) according to Method 1. The crude product was purified by column chromatography (hexane/ethyl acetate 19:1 to 17:3) to afford **11a** (45 mg, 92 %). R_f = 0.31 (hexane/ethyl acetate 17:3). Melting point = 93 °C (Lit:[20] 92-93 °C). ^1H NMR (400 MHz, $CDCl_3$) δ = 7.07 (d, *J*=1.5 Hz, 2H), 7.36 – 7.40 (m, 3H), 7.42 – 7.48 (m, 4H), 7.62 -7.64 (m, 4H) ppm; ^{13}C NMR (75 MHz, $CDCl_3$) δ = 113.25, 118.99, 127.33, 127.71, 128.90, 140.92, 143.49, 156.43 ppm; UV/Vis: λ (nm) = 303, 244, 214; IR (KBr): $\tilde{\nu}$ = 3632, 3622, 3590, 3435, 3409, 3061, 3031, 2920, 2849, 2352, 1642, 1594, 2577, 1497, 1459, 1339, 1181, 1075, 1063, 1030, 924, 910, 861, 786, 759, 698, 661, 639, 612, 534, 487, 470, 457, 448, 433, 427, 416, 406; HRMS (ESI) found: $[M+H^+]$ m/z = 247.1125, calcd. for $C_{18}H_{15}O^+$ = 247.1117.

[1,1':3',1''-terphenyl]-5'-yl dibenzyl phosphate (**11b**)

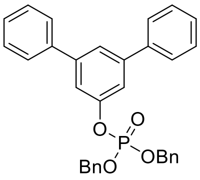

11b was obtained from **11a** (40 mg, 0.16 mmol) according to Method 3. The crude product was purified by column chromatography (hexane/ethyl acetate 9:1 → 4:1), yielding **11b** as colorless oil (55 mg, 67 %). R_f = 0.13 (hexane/ethyl acetate 17:3); ^{1}H NMR (400 MHz, $CDCl_3$) δ = 5.12 - 5.24 (m, 4H), 7.29 – 7.41 (m, 14H), 7.41 – 7.47 (m, 4H), 7.52 - 7.56 (m, 4H), 7.60 (s, 1H) ppm; ^{13}C NMR (101 MHz, $CDCl_3$) δ = 70.22 (d, *J*=5.8 Hz), 117.79 (d, *J*=4.8 Hz), 122.93, 127.37, 127.96, 128.26, 128.63, 128.76, 128.95, 135.55 (d, *J*=6.7 Hz), 140.17, 143.48, 151.39 (d, *J*=7.0 Hz) ppm; ^{31}P NMR (162 MHz, $CDCl_3$) δ = -6.2 (s, 1P) ppm; UV/Vis: λ (nm) = 248, 213; IR (KBr): $\tilde{\nu}$ = 3433, 3066, 3036, 1591, 1576, 1554, 1278, 1012, 1001, 965, 785, 762, 697, 638, 600, 489, 461, 447, 424, 416, 406; HRMS (ESI) found: [M+Na$^+$] m/z = 529.1535, calcd. for $C_{32}H_{27}NaO_4P^+$: 529.1539.

[1,1':3',1''-terphenyl]-5'-yl dihydrogen phosphate (**11**)

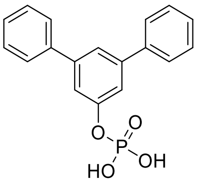

11 was obtained from **11b** (50 mg, 0.10 mmol) according to Method 4. After lyophilization, the product was obtained as a fluffy white solid (32 mg, 99 %). Decomposition point: 169 °C; ^{1}H NMR (400 MHz, DMSO-*d*$_6$) δ = 7.38 - 7.45 (m, 4H), 7.46 – 7.53 (m, 4H), 7.66 (s, 1H), 7.71 – 7.77 (m, 4H) ppm; ^{13}C NMR (101 MHz, DMSO-*d*$_6$) δ = 117.38 (d, *J*=4.8 Hz), 120.46, 126.93, 127.85, 128.97, 139.57, 142.17, 152.89 (d, *J*=6.1 Hz) ppm; ^{31}P NMR (162 MHz, DMSO-*d*$_6$) δ = -5.7 (s, 1P) ppm; UV/Vis: λ (nm) = 248, 206; IR (KBr): $\tilde{\nu}$ = 3441, 3107, 3087, 3062, 3048, 3037, 2956, 2924, 2852, 2660, 2317, 1961, 1892, 1801, 1763, 1748, 1733, 1593, 1576, 1501, 1459, 1436, 1408, 1332, 1196, 1174, 1136, 1078, 1065, 1037, 1011, 1001, 980, 921, 915, 890, 874, 827, 818, 758, 698, 661, 628, 612, 569, 553, 532, 508, 500, 485, 438, 425; HRMS (ESI) found: [M+H$^+$] m/z = 327.0794, calcd. for $C_{18}H_{16}O_4P^+$: 327.0781.

4,4''-dimethoxy-[1,1':3',1''-terphenyl]-5'-ol (**12a**)[21]

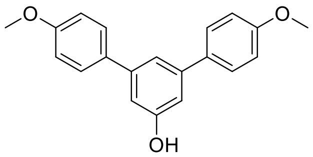

12a was obtained from 3,5-dibromophenol (100 mg, 0.40 mmol) and 4-methoxyphenylboronic acid (181 mg, 1.20 mmol) according to Method 1. The crude product was purified by column chromatography (hexane/ethyl acetate 9:1→ 4:1) to afford **12a** as a colorless oil (110 mg, 90 %). R_f = 0.23 (hexane/ethyl acetate 4:1); ^{1}H NMR (400 MHz, $CDCl_3$) δ = 3.86 (s, 6H), 6.94 - 7.01 (m, 6H), 7.29 - 7.31 (m, 1H), 7.52 - 7.58 (m, 4H) ppm; ^{13}C NMR (101 MHz, $CDCl_3$) δ = 55.51, 112.21, 114.34, 118.26, 128.34, 133.51, 143.06, 156.29, 159.47 ppm; UV/Vis: λ (nm) = 260, 210; IR (KBr): $\tilde{\nu}$ = 3467, 3059, 3035, 3001, 2960, 2934, 2914, 2838, 2532, 2046, 1880, 1608, 1576, 1519, 1497, 1484, 1466, 1440, 1421, 1397, 1371, 1340, 1308, 1290, 1254, 1240, 1117, 998, 917, 869, 858, 837, 825, 811, 793, 759, 745, 726, 696, 670, 640, 609, 594, 579, 554, 541, 521, 508; HRMS (ESI) found: [M+H$^+$], m/z = 307.1329, calcd. for $C_{20}H_{19}O_3^+$: 307.1329.

Dibenzyl (4,4''-dimethoxy-[1,1':3',1''-terphenyl]-5'-yl) phosphate (**12b**)

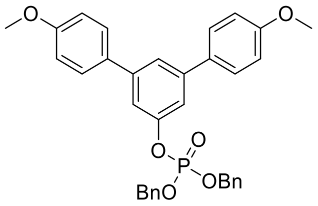

12b was obtained from **12a** (100 mg, 0.33 mmol) according to Method 3. The crude product was purified by column chromatography (hexane/ethyl acetate 17:3 → 4:1), yielding **12b** as colorless oil (129 mg, 70 %). R_f = 0.13 (hexane/ethyl acetate 4:1); ^{1}H NMR (400 MHz, $CDCl_3$) δ = 3.89 (s, 6H), 5.18 – 5.28 (m, 4H), 7.01 (d, *J*=8.7 Hz, 4H), 7.30 (s, 2H), 7.35 – 7.42 (m, 10H), 7.52 (d, *J*=8.7 Hz, 4H), 7.57 (s, 1H) ppm; ^{13}C NMR (101 MHz, $CDCl_3$) δ = 55.42, 70.11 (d, *J*=5.8 Hz), 114.29, 116.65 (d, *J*=4.8 Hz), 121.94, 128.17, 128.33, 128.68, 128.73, 132.70, 135.55 (d, *J*=6.8 Hz), 142.95, 151.35 (d, *J*=7.0 Hz), 159.59 ppm; ^{31}P NMR (162 MHz, $CDCl_3$) δ = -6.1 (p, *J*=8.6 Hz, 1P) ppm; UV/Vis: λ (nm) = 268, 205; IR (KBr): $\tilde{\nu}$ = 3569, 3520, 3535, 3348, 2955, 2932, 2836, 1606, 1589, 1574, 1516, 1456, 1440, 1394, 1384, 1285, 1252, 1215, 1177, 1034, 1011, 1000, 965, 879, 856, 828, 739, 697, 595, 568, 492, 463, 456, 447, 424, 417, 405; HRMS (ESI) found: [M+Na$^+$] m/z = 589.1746, calcd. for $C_{34}H_{31}NaO_6P^+$: 589.1750.

[4,4''-dimethoxy-[1,1':3',1''-terphenyl]-5'-yl dihydrogen phosphate (**12**)

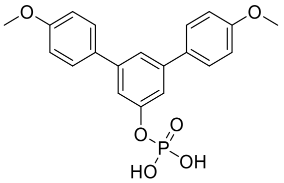

12 was obtained from **12b** (85 mg, 0.15 mmol) according to Method 4. The product was obtained as a fluffy off white solid (57 mg, 98 %). Melting point = 118 °C; ^{1}H NMR (300 MHz,

DMSO-d_6) δ = 3.80 (s, 6H), 7.03 (d, J=8.7 Hz, 4H), 7.32 (s, 2H), 7.55 (s, 1H), 7.66 (d, J=8.7 Hz, 4H) ppm; ^{13}C NMR (101 MHz, DMSO-d_6) δ = 55.20, 114.36, 116.19, 119.49, 128.00, 131.99, 141.72, 152.78 (d, J=6.4 Hz), 159.16 ppm; ^{31}P NMR (162 MHz, DMSO-d_6) δ = -5.7 (s, 1P) ppm; UV/Vis: λ (nm) = 265, 208; IR (KBr): $\tilde{\nu}$ = 3707, 3453, 3035, 3000, 2958, 2934, 2913, 2837, 2540, 2360, 2336, 2044, 1770, 1748, 1732, 1715, 1608, 1591, 1575, 1517, 1456, 1440, 1412, 1395, 1336, 1288, 1254, 1238, 1179, 1115, 1086, 1065, 1031, 1013, 1004, 974, 876, 827, 783, 759, 727, 698, 644, 630, 609, 593, 567, 554, 525, 500, 434; HRMS (ESI) found: [M+H$^+$] m/z = 387.1002, calcd. for $C_{20}H_{20}O_6P^+$: 387.0992.

3,3''-dimethoxy-[1,1':3',1''-terphenyl]-5'-ol (**13a**)[22]

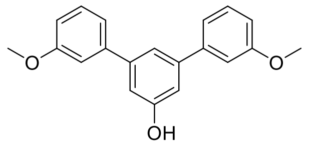

13a was obtained from 3,5-dibromophenol (200 mg, 0.79 mmol) and 3-methoxyphenylboronic acid (302 mg, 1.98 mmol) according to Method 1. The crude product was purified by column chromatography (DCM/MeOH 200:1) to afford **13a** as pale brown liquid (200 mg, 82%). R_f = 0.23 (DCM/MeOH, 100:1); ^{1}H NMR (400 MHz, $CDCl_3$) δ = 3.91 (s, 6H), 6.43 (s, 1H, br), 6.97 – 7.02 (m, 2H), 7.13 – 7.16 (m, 1H), 7.22 – 7.28 (m, 3H), 7.37 – 7.44 (m, 2H), 7.45 – 7.47 (m, 1H) ppm; ^{13}C NMR (101 MHz, $CDCl_3$) δ = 55.47, 113.04, 113.21, 113.47, 118.91, 119.94, 129.94, 142.37, 143.25, 156.33, 159.84 ppm; UV/Vis: λ (nm) = 290, 241, 219; IR (KBr): $\tilde{\nu}$ = 3398, 3001, 2954, 2937, 2835, 1598, 1579, 1492, 1475, 1467, 1429, 1399, 1313, 1301, 1285, 1267, 1235, 1202, 1187, 1173, 1092, 1046, 993, 955, 918, 908, 850, 810, 781, 751, 696, 659, 620, 564, 449, 404 cm^{-1}; HRMS (ESI) found: [M+Na$^+$] m/z = 329.1147, calcd. for $C_{20}H_{19}NaO_3^+$: 329.1148.

Dibenzyl (3,3''-dimethoxy-[1,1':3',1''-terphenyl]-5'-yl) phosphate (**13b**)

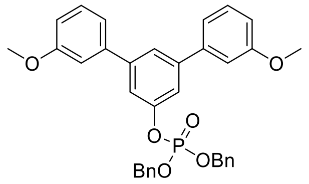

13b was obtained from **13a** (110 mg, 0.36 mmol) according to Method 3. The crude product was purified by column chromatography (hexane/ethyl acetate 9:1 → 4:1), providing **13b** as colorless oil (180 mg, 88 %). R_f = 0.18 (hexane/ethyl acetate 4:1); ^{1}H NMR (400 MHz, $CDCl_3$) δ = 3.90 (s, 6H), 5.22 – 5.31 (m, 4H), 6.97 - 7.03 (m, 2H), 7.17 -7.24 (m, 4H), 7.34 - 7.48 (m, 14H), 7.69 (s, 1H) ppm; ^{13}C NMR (101 MHz, $CDCl_3$) δ = 55.46, 70.20 (d, J=5.8 Hz), 113.08, 113.37, 117.95 (d, J=4.7 Hz), 119.86, 122.98, 128.19, 128.72, 128.79, 129.94, 135.52 (d, J=6.7 Hz), 141.63, 143.34, 151.33 (d, J=7.1 Hz), 160.09 ppm; ^{31}P NMR (162 MHz, $CDCl_3$) δ =

-6.2 (s, 1P) ppm; UV/Vis: λ (nm) = 251, 215, 203; IR (KBr): $\tilde{\nu}$ = 3567, 3545, 3502, 3482, 3064, 3033, 3003, 2957, 2940, 2898, 2835, 1599, 1578, 1543, 1496, 1456, 1441, 1425, 1396, 1338, 1286, 1229, 1203, 1173, 1082, 1039, 1007, 997, 982, 920, 876, 811, 781, 749, 740, 696, 620, 600, 540, 524, 492 cm^{-1}; HRMS (ESI) found: $[2M+H^+]$ m/z = 1133.3784, calcd. for $C_{68}H_{63}O_{12}P_2^+$ = 1133.3789.

3,3''-dimethoxy-[1,1':3',1''-terphenyl]-5'-yl dihydrogen phosphate (**13**)

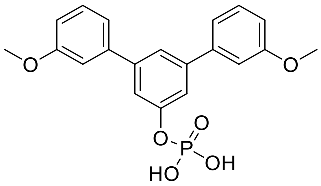

13 was obtained from **13b** (160 mg, 0.28 mmol) according to Method 4. After lyophilization, the product was obtained as a fluffy white solid (105 mg, 96 %). Melting point = 74 °C; ^{1}H NMR (400 MHz, DMSO-d_6) δ = 3.82 (s, 6H), 6.94 (d, J=7.6 Hz, 2H), 7.20 – 7.29 (m, 4H), 7.33 -7.39 (m, 2H), 7.42 (s, 2H), 7.55 (s, 1H) ppm; ^{13}C NMR (101 MHz, DMSO-d_6) δ = 55.62, 112.84, 113.79, 118.19, 119.75, 120.23, 130.39, 142.06 (d, J=33.0 Hz), 154.24 (d, J=4.2 Hz), 160.17 ppm; ^{31}P NMR (162 MHz, DMSO-d_6) δ = -5.1 (s, 1P) ppm; UV/Vis: λ (nm) = 257, 216; IR (KBr): = 3465, 3457, 2938 2837, 2359, 2335, 1600, 1579, 1495, 1466, 1426, 1397, 1340, 1287, 1266, 1228, 1203, 1173, 1092, 1066, 1045, 1006, 997, 984, 852, 778, 750, 695, 547, 526, 517, 503, 477, 470, 454 cm^{-1}; HRMS (ESI) found: $[M-H^+]$ m/z = 385.0845, calcd. for $C_{20}H_{18}O_6P^-$: 385.0846.

2,2''-dimethoxy-[1,1':3',1''-terphenyl]-5'-ol (**14a**)

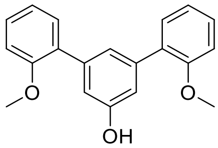

14a was obtained from 3,5-dibromophenol (150 mg, 0.60 mmol) and 2-methoxyphenylboronic acid (272 mg, 1.79 mmol) according to Method 1. The crude product was purified by column chromatography in hexane/ethyl acetate (19:1→ 6:1) to afford **14a** as yellow liquid (145 mg, 80 %). R_f = 0.75 (DCM/acetone 9:1); ^{1}H NMR (400 MHz, $CDCl_3$) δ = 3.82 (s, 6H), 6.97 – 7.08 (m, 6H), 7.30 (s, 1H), 7.32 – 7.41 (m, 4H) ppm; ^{13}C NMR (75 MHz, $CDCl_3$) δ = 55.67, 111.40, 115.46, 120.91, 123.57, 128.75, 130.48, 131.00, 139.63, 154.76, 156.54 ppm; UV/Vis: λ (nm) = 289, 243,221; IR (KBr): $\tilde{\nu}$ = 3398, 3061, 3003, 2955, 2937, 2834, 1593, 1496, 1463, 1435, 1414, 1338, 1290, 1274, 1246, 1180, 1122, 1081, 1061, 1047, 1026, 999, 920, 867, 840, 788, 754, 704, 650, 617, 594, 569, 490, 469, 456, 403 cm^{-1}; HRMS (ESI) found: $[M+Na^+]$ m/z = 329.1148, calcd. for $C_{20}H_{19}NaO_3^+$: 329.1148.

Dibenzyl (2,2''-dimethoxy-[1,1':3',1''-terphenyl]-5'-yl) phosphate (**14b**)

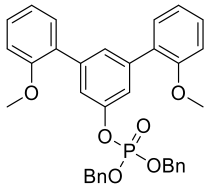

14b was obtained from **14a** (140 mg, 0.46 mmol) according to Method 3. The crude product was purified by column chromatography (hexane/ethyl acetate 9:1 → 4:1), providing **14b** as colorless oil (233 mg, 89 %). R_f = 0.175 (hexane/ethyl acetate 4:1); ^{1}H NMR (400 MHz, $CDCl_3$) δ = 3.80 (s, 3H), 3.80 (s, 3H), 5.17 – 5.24 (m, 4H), 6.97 – 7.09 (m, 4H), 7.29 – 7.43 (m, 16H), 7.57 (d, *J*=6.2 Hz, 1H) ppm; ^{13}C NMR (101 MHz, $CDCl_3$) δ = 55.57, 69.95 (d, *J*=5.7 Hz), 111.32, 119.84 (d, *J*=4.8 Hz), 120.87, 127.67, 128.05, 128.60, 129.00, 129.72, 130.97, 135.64 (d, *J*=7.0 Hz), 139.71, 149.89 (d, *J*=7.1 Hz), 156.52 ppm; ^{31}P NMR (162 MHz, $CDCl_3$) δ = -6.2 (s, 1P) ppm; UV/Vis: λ (nm) = 289, 248, 207; IR (KBr): $\tilde{\nu}$ = 3501, 3461, 3446, 3064, 3033, 3005, 2956, 2939, 2899, 2835, 1602, 1587, 1496, 1463, 1467, 1433, 1410, 1381, 1333, 1279, 1247, 1216, 1178, 1170, 1123, 1082, 1025, 965, 919, 883, 859, 840, 787, 754, 697, 653, 617, 600, 499, 472 cm^{-1}; HRMS (ESI) found: [$M+H^+$], m/z = 567.1933, calcd. for $C_{34}H_{32}O_6P^+$: 567.1931.

2,2''-dimethoxy-[1,1':3',1''-terphenyl]-5'-yl dihydrogen phosphate (**14**)

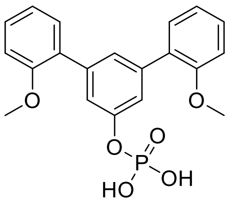

14 was obtained from **14b** (230 mg, 0.41 mmol) according to Method 4. After lyophilization, the product was obtained as a fluffy white solid (150 mg, 96 %). Melting point = 94 °C; ^{1}H NMR (400 MHz, DMSO-d_6) δ = 3.77 (s, 6H), 6.99 - 7.05 (m, 2H), 7.11 (d, *J*=8.2 Hz, 2H), 7.22 (s, 2H), 7.28 – 7.38 (m, 5H) ppm; ^{13}C NMR (101 MHz, DMSO-d_6) δ = 55.48, 111.78, 119.65, 120.78, 125.43, 129.02, 129.25, 130.34, 138.69, 151.37 (d, *J*=6.2 Hz), 156.09 ppm; ^{31}P NMR (162 MHz, DMSO-d_6) δ = -5.6 (s, 1P) ppm; UV/Vis: λ (nm) = 286, 246, 213; IR (KBr): $\tilde{\nu}$ = 3612, 3589, 3567, 3545, 3524, 3502, 3483, 2935, 2374, 2349, 2322, 1744, 1732, 1715, 1587, 1496, 1458, 1433, 1409, 1335, 1277, 1245, 1166, 1121, 1062, 1046, 1025, 1006, 971, 880, 841, 821, 783, 751, 703, 653, 638, 616, 599, 490, 455, 418 cm^{-1}; HRMS (ESI) found: [$M-H^+$] m/z = 385.0843, calcd. for $C_{20}H_{18}O_6P^-$ = 385.0846.

3,3'',4,4''-tetramethoxy-[1,1':3',1''-terphenyl]-5'-ol (**15a**)

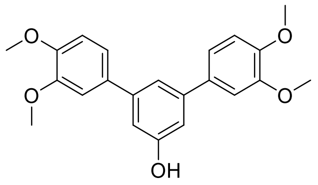

15a was obtained from 3,5-dibromophenol (150 mg, 0.60 mmol) and 3,4-dimethoxyphenylboronic acid (271 mg, 1.49 mmol) according to Method 1. The crude product was purified by column chromatography (DCM/MeOH 100:1) to afford **15a** as pale brown solid (180 mg, 82 %). R_f = 0.18 (DCM/MeOH 100:1); Melting point = 165 °C; ^{1}H NMR (400 MHz, $CDCl_3$) δ = 3.93 (s, 6H), 3.94 (s, 6H), 6.93 – 7.00 (4H), 7.11 – 7.20 (4H), 7.28 - 7.30 (1H) ppm; ^{13}C NMR (75 MHz, $CDCl_3$) δ = 56.14, 110.67, 111.60, 112.58, 118.42, 119.64, 133.99, 143.34, 148.96, 149.25, 156.35 ppm; UV/Vis: λ (nm) = 284, 258, 218, 215; IR (KBr): $\tilde{\nu}$ = 3671, 3440, 3003, 2949, 2936, 2906, 2837, 1604, 1598, 1583, 1520, 1495, 1462, 1452, 1435, 1406, 1330, 1311, 1285, 1254, 1233, 1202, 1167, 1142, 1088, 1068, 1025, 995, 969, 934, 882, 849, 832, 816, 807, 769, 748, 707, 664, 650, 620, 596, 533, 485, 457 cm^{-1}; HRMS (ESI) found: [$M+Na^+$] m/z = 389.1358, calcd. for $C_{22}H_{23}NaO_5^+$: 389.1358.

Dibenzyl (3,3'',4,4''-tetramethoxy-[1,1':3',1''-terphenyl]-5'-yl) phosphate (**15b**)

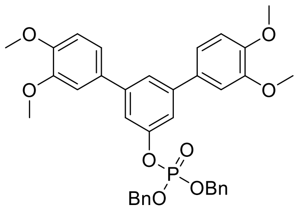

15b was obtained from **15a** (100 mg, 0.27 mmol) according to Method 3. The crude product was purified by column chromatography (hexane/ethyl acetate 4:1 → 13:7), providing **15b** as colorless oil (120 mg, 70 %). R_f = 0.40 (DCM/acetone 19:1); ^{1}H NMR (400 MHz, $CDCl_3$) δ = 3.94 (s, 6H), 3.96 (s, 6H), 5.10 – 5.30 (m, 4H), 6.96 (d, *J*=8.3 Hz, 2H), 7.07 – 7.10 (m, 2H), 7.11 – 7.17 (m, 2H), 7.29 – 7.40 (m, 12H), 7.53 (s, 1H) ppm; ^{13}C NMR (75 MHz, $CDCl_3$) δ = 56.08, 56.09, 70.11 (d, *J*=5.8 Hz), 110.55, 111.51, 116.96, 116.99 (d, *J*=4.9 Hz), 122.30, 128.09, 128.66, 128.72, 133.17, 135.48 (d, *J*=6.9 Hz), 143.29, 149.12, 149.26, 151.30 (d, *J*=7.1 Hz) ppm; ^{31}P NMR (162 MHz, $CDCl_3$) δ = -6.1 (s, 1P) ppm; UV/Vis: λ (nm) = 291, 268, 213; HRMS (ESI) found: [$2M+H^+$] m/z = 1253.4218, calcd. for $C_{72}H_{71}O_{16}P_2^+$: 1253.4212.

3,3'',4,4''-tetramethoxy-[1,1':3',1''-terphenyl]-5'-yl dihydrogen phosphate (**15**)

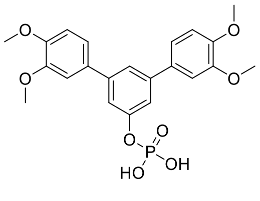

15 was obtained from **15b** (100 mg, 0.16 mmol) according to Method 4. After lyophilization, the product was obtained as a fluffy off white solid (70 mg, 98 %). Melting point = 177 °C; ^{1}H NMR (400 MHz, DMSO-d_6) δ = 3.78 (s, 6H), 3.84 (s, 6H), 6.98 - 7.04 (m, 2H), 7.19 – 7.25 (m, 4H), 7.35 - 7.42 (m, 3H) ppm; ^{13}C NMR (101 MHz, DMSO-d_6) δ = 55.56, 55.63, 110.76, 112.12, 116.88, 119.10, 133.26, 141.37, 148.47, 148.95 ppm; ^{31}P NMR (162 MHz, DMSO-d_6) δ = -6.0 (s, 1P) ppm; UV/Vis: λ (nm) = 293, 264, 203; IR (KBr): $\tilde{\nu}$ = 3684, 3444, 2937, 2838, 2360, 2323, 1633, 1605, 1580, 1519, 1465, 1439, 1390, 1315, 1264, 1254, 1228, 1204, 1184, 1169, 1145, 1088, 1071, 1023, 982, 935, 858, 809, 788, 768, 748, 660, 622, 594, 548, 471, 457 cm^{-1}; HRMS (ESI) found: [M-H^+] m/z = 445.1054, calcd. for $C_{22}H_{22}O_8P^-$ = 445.1058.

2,2'',3,3''-tetramethoxy-[1,1':3',1''-terphenyl]-5'-ol (**16a**)

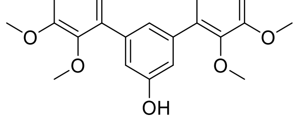

16a was obtained from 3,5-dibromophenol (150 mg, 0.60 mmol) and 2,3-dimethoxyphenylboronic acid (325 mg, 1.79 mmol) according to Method 1. The crude product was purified by column chromatography (DCM/MeOH 200:1 → 100:1) to afford off-white solid (190 mg, 87 %). R_f = 0.35 (DCM/MeOH, 50:1); Melting point = 135 °C; ^{1}H NMR (400 MHz, $CDCl_3$) δ = 3.64 (s, 6H), 3.90 (s, 6H), 6.36 – 6.44 (m, 2H), 6.91 (d, *J*=8.2 Hz, 1H), 6.99 (d, *J*=7.7 Hz, 1H), 7.05 – 7.15 (m, 4H), 7.27 - 7.30 (m, 1H) ppm; ^{13}C NMR (101 MHz, $CDCl_3$) δ = 56.02, 60.74, 111.60, 115.29, 122.56, 122.85, 124.18, 135.77, 139.36, 146.54, 153.04, 155.64 ppm; UV/Vis: λ (nm) = 289, 241,219; IR (KBr): $\tilde{\nu}$ = 3433, 2994, 2969, 2938, 2904, 2837, 1594, 1577, 1496, 1474, 1464, 1437, 1425, 1407, 1338, 1305, 1293, 1264, 1224, 1205, 1186, 1171,1120, 1088, 1064, 1043, 1008, 1000, 957, 924, 892, 860, 825, 813, 793, 783, 772, 754, 738, 725, 704, 636, 602, 538, 506 cm^{-1}; HRMS (ESI) found: [M+Na^+] m/z = 389.1356, calcd. for $C_{22}H_{22}NaO_5^+$: 389.1359.

Dibenzyl (2,2'',3,3''-tetramethoxy-[1,1':3',1''-terphenyl]-5'-yl) phosphate (**16b**)

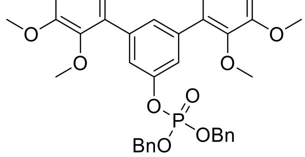

16b was obtained from **16a** (150 mg, 0.41 mmol) according to Method 3. The crude product was purified by column chromatography (hexane/ethyl acetate 9:1 → 3:1), providing **17b** as colorless oil (220 mg, 86%). R_f = 0.15 (hexane/ethyl acetate 4:1); ^{1}H NMR (400 MHz, $CDCl_3$) δ = 3.62 (s, 6H), 3.91 (s, 6H), 5.16 (d, *J*=8.3 Hz, 4H), 6.90 – 6.96 (m, 4H), 7.05 – 7.13 (m, 2H), 7.27 – 7.36 (m, 8H), 7.38 (s, 2H), 7.60 (s, 1H) ppm; ^{13}C NMR (101 MHz, $CDCl_3$) δ = 56.11,

60.82, 70.04 (d, *J*=5.8 Hz), 112.11, 119.74 (d, *J*=4.8 Hz), 122.75, 124.16, 127.31, 128.13, 128.67, 134.89, 135.63 (d, *J*=6.9 Hz), 139.58, 146.76, 150.23 (d, *J*=7.2 Hz), 153.24 ppm; ^{31}P NMR (162 MHz, $CDCl_3$) δ = -6.2 (s, 1P) ppm; UV/Vis: λ (nm) = 280, 246, 209; IR (KBr): $\tilde{\nu}$ = 3065, 3033, 2998, 2957, 2936, 2898, 2836, 1607, 1587, 1576, 1496, 1473, 1456, 1437, 1423, 1403, 1340, 1265, 1229, 1199, 1173, 1120, 1084, 1060, 1035, 1010, 984, 922, 881, 811, 789, 763, 746, 720, 697, 602, 502, 471, 457 cm^{-1}; HRMS (ESI) found: [$M+H^+$] m/z = 627.2145, calcd. for $C_{36}H_{36}O_8P^+$: 627.2142.

<u>2,2'',3,3''-tetramethoxy-[1,1':3',1''-terphenyl]-5'-yl dihydrogen phosphate (**16**)</u>

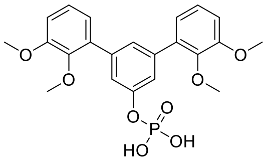

16 was obtained from **16b** (200 mg, 0.32 mmol) according to Method 4. After lyophilization, the product was obtained as a fluffy white solid (140 mg, 98 %). Melting point = 83 °C; ^{1}H NMR (400 MHz, DMSO-d_6) δ = 3.61 (s, 6H), 3.85 (s, 6H), 6.92 - 6.97 (m, 2H), 7.04 – 7.10 (m, 2H), 7.10 – 7.16 (m, 2H), 7.26 (s, 2H), 7.30 (s, 1H) ppm; ^{13}C NMR (101 MHz, DMSO-d_6) δ = 55.78, 60.24, 112.40, 119.40, 122.02, 124.13, 124.36, 134.57, 138.44, 145.98, 152.87 ppm; ^{31}P NMR (162 MHz, DMSO-d_6) δ = -5.3 (s, 1P) ppm; UV/Vis: λ (nm) = 241, 220, 219; IR (KBr): $\tilde{\nu}$ = 3458, 2937, 2837, 2350, 2335, 2322, 1768, 1747, 1732, 1715, 1681, 1633, 1589, 1576, 1481, 1474, 1437, 1424, 1405, 1342, 1304, 1290, 1264, 1227, 1200, 1173, 1120, 1087, 1060, 1006, 984, 887, 874, 850, 811, 790, 766, 748, 719, 703, 651, 600, 542, 504 cm^{-1}; HRMS (ESI) found: [M-H^+] m/z = 445.1053, calcd. for $C_{22}H_{22}O_8P^-$: 445.1058.

<u>3,3'',5,5''-tetramethoxy-[1,1':3',1''-terphenyl]-5'-ol (**17a**)</u>

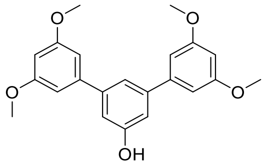

17a was obtained from 3,5-dibromophenol (150 mg, 0.60 mmol) and 3,5-dimethoxyphenylboronic acid (325 mg, 1.79 mmol) according to Method 1. The crude product was purified by column chromatography (DCM/MeOH 200:1) to afford **17a** as pale yellow liquid (165 mg, 76 %). R_f = 0.23 (DCM/MeOH 100:1); ^{1}H NMR (400 MHz, $CDCl_3$) δ = 3.85 (s, 12H), 5.24 (s, 1H), 6.49 (t, *J*=2.3 Hz, 2H), 6.75 (d, *J*=2.3 Hz, 4H), 7.03 (d, *J*=1.5 Hz, 2H), 7.34 (t, *J*=1.5 Hz, 1H) ppm; ^{13}C NMR (75 MHz, $CDCl_3$) δ = 55.53, 99.74, 105.65, 113.57, 118.81, 143.21 (d, *J*=16.1 Hz), 156.31, 161.02 ppm; UV/Vis: λ (nm) = 289, 251, 220; IR (KBr): $\tilde{\nu}$ = 3587, 3566, 3522, 3409, 3000, 2957, 2937, 2839, 1590, 1542, 1506, 1456, 1438, 1423, 1398, 1339, 1319, 1299, 1269, 1251, 1204, 1173, 1154, 1084, 1065, 991, 975, 941, 927, 832, 787,

777, 764, 692, 679, 542 cm^{-1}; HRMS (ESI) found: [M+H$^+$] m/z = 367.1537, calcd. for $C_{22}H_{23}O_5^+$ = 367.1540.

Dibenzyl (3,3'',5,5''-tetramethoxy-[1,1':3',1''-terphenyl]-5'-yl) phosphate (**17b**)

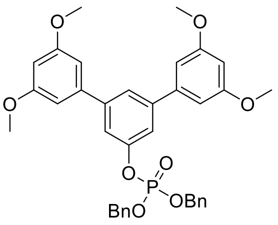

17b was obtained from **17a** (160 mg, 0.44 mmol) according to Method 3. The crude product was purified by column chromatography (hexane/ethyl acetate 9:1 → 4:1), providing **17b** as colorless oil (240 mg, 87%). R_f = 0.18 (hexane/ethyl acetate 4:1); ^{1}H NMR (400 MHz, $CDCl_3$) δ = 3.85 (s, 12H), 5.16 - 5.25 (m, 4H), 6.51 - 6.56 (m, 2H), 6.72 - 6.76 (m, 4H), 7.30 – 7.41 (m, 12H), 7.61 (s, 1H) ppm; ^{13}C NMR (101 MHz, $CDCl_3$) δ = 55.49, 70.15 (d, *J*=5.8 Hz), 99.85, 105.63, 118.03 (d, *J*=4.7 Hz), 122.95, 128.10, 128.66, 128.73, 135.43 (d, *J*=6.7 Hz), 142.25, 143.41, 151.14 (d, *J*=7.0 Hz), 161.17 ppm; ^{31}P NMR (162 MHz, $CDCl_3$) δ = -6.2 (s, 1P) ppm; UV/Vis: λ (nm) = 255, 206; IR (KBr): $\tilde{\nu}$ = 3588, 3567, 3545, 3524, 3502, 3482, 3065, 3033, 3000, 2958, 2938, 2903, 2838, 2359, 1584, 1498, 1456, 1435, 1420, 1395, 1337, 1320, 1286, 1261, 1204, 1155, 1083, 1064, 1009, 997, 986, 927, 886, 834, 738, 695, 678, 603, 501, 491, 456, 418 cm^{-1}; HRMS (ESI) found: [M+H$^+$] m/z = 627.2144, calcd. for $C_{36}H_{36}O_8P^+$: 627.2142.

3,3'',5,5''-tetramethoxy-[1,1':3',1''-terphenyl]-5'-yl dihydrogen phosphate (**17**)

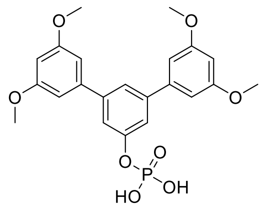

17 was obtained from **17b** (220 mg, 0.35 mmol) according to Method 4. After lyophilization, the product was obtained as a fluffy pale brown solid (150 mg, 97 %). Melting point = 66 °C; ^{1}H NMR (400 MHz, DMSO-d_6) δ = 3.82 (s, 12H), 6.54 (s, 2H), 6.84 (d, *J*=2.1 Hz, 4H), 7.41 (s, 2H), 7.62 (s, 1H) ppm; ^{13}C NMR (101 MHz, DMSO-d_6) δ = 55.33, 99.71, 105.21, 117.83 (d, *J*=4.4 Hz), 120.88, 141.79, 142.16, 152.47 (d, *J*=6.2 Hz), 160.85 ppm; ^{31}P NMR (162 MHz, DMSO-d_6) δ = -6.0 (s, 1P) ppm; UV/Vis: λ (nm) = 295, 246, 203; IR (KBr): $\tilde{\nu}$ = 3448, 3085, 2999, 2957, 2941, 2839, 2346, 2322, 1826, 1588, 1459, 1434, 1420, 1395, 1338, 1319, 1300, 1260, 1248, 1205, 1156, 1065, 1052, 1011, 987, 957, 928, 880, 835, 755, 689, 678, 647, 586, 537, 526, 516, 502, 491, 454, 433 cm^{-1}; HRMS (ESI) found: [2M-H$^+$] m/z = 891.2189, calcd. for $C_{44}H_{45}O_{16}P_2^-$: 891.2188.

3,5-bis(benzo[d][1,3]dioxol-5-yl)phenol (**18a**)

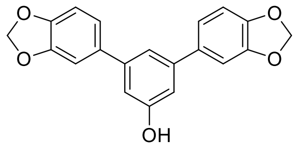

18a was obtained from 3,5-dibromophenol (150 mg, 0.60 mmol) and 3,4-(methylenedioxy)phenylboronic acid (247 mg, 1.49 mmol) according to Method 1. The crude product was purified by column chromatography (DCM/MeOH 200:1 → 100:1) to afford **18a** as pale brown solid (143 mg, 72 %). Decomposition point: 202 °C; R_f = 0.25 (DCM/MeOH 100:1); ^{1}H NMR (400 MHz, $CDCl_3$) δ = 5.96 (s, 4H), 6.84 (d, *J*=8.5 Hz, 2H), 6.91 (s, 2H), 7.03 - 7.09 (m, 4H), 7.14 (s, 1H) ppm; ^{13}C NMR (75 MHz, $CDCl_3$) δ = 101.21, 107.75, 108.57, 112.61, 117.55, 120.72, 135.54, 142.84, 147.20, 148.07, 157.29 ppm; UV/Vis: λ (nm) = 299, 265, 211; IR (KBr): = $\tilde{\nu}$ = 3538, 3445, 3077, 3065, 3000, 2898, 2786, 2361, 1855, 1595, 1571, 1505, 1491, 1474, 1455, 1427, 1404, 1342, 1321, 1279, 1239, 1222, 1183, 1145, 1114, 1065, 1037, 996, 956, 934, 906, 880, 854, 813, 782, 769, 746, 720, 702, 667, 635, 595, 581, 561, 531, 521, 452, 434, 419 cm^{-1}; HRMS (ESI) found: [M-H^+] m/z = 333.0768, calcd. for $C_{20}H_{13}O_5^-$ = 333.0769.

Dibenzyl (3,5-bis(benzo[d][1,3]dioxol-5-yl)phenyl) phosphate (**18b**)

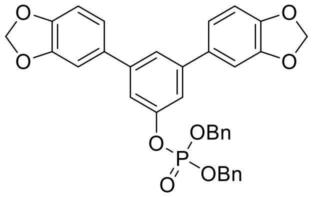

18b was obtained from **18a** (140 mg, 0.42 mmol) according to Method 3. The crude product was purified by column chromatography (hexane/ethyl acetate 9:1 → 4:1), providing **18b** as colorless oil (220 mg, 88 %). R_f = 0.25 (hexane/ethyl acetate, 4:1); ^{1}H NMR (400 MHz, $CDCl_3$) δ = 5.12 – 5.26 (m, 4H), 6.00 (s, 4H), 6.87 (d, *J*=7.7 Hz, 2H), 6.99 (s, 1H), 7.01 (s, 3H), 7.21 (s, 2H), 7.30 – 7.41 (m, 10H), 7.44 (s, 1H) ppm; ^{13}C NMR (101 MHz, $CDCl_3$) δ = 70.14 (d, *J*=5.8 Hz), 101.33, 107.73, 108.63, 117.09 (d, *J*=4.7 Hz), 120.90, 122.23, 128.18, 128.70, 128.78, 134.38, 135.51 (d, *J*=6.6 Hz), 143.04, 147.58, 148.25, 151.26 (d, *J*=7.0 Hz) ppm; ^{31}P NMR (162 MHz, $CDCl_3$) δ = -6.2 (s, 1P) ppm; UV/Vis: λ (nm) = 295, 269, 202; IR (KBr): $\tilde{\nu}$ = 3649, 3629, 3567, 3545, 3524, 3481, 3065, 3033, 3009, 2958, 2894, 2779, 2320, 1846, 1749, 1733, 1717, 1604, 1585, 1507, 1499, 1458, 1427, 1413, 1382, 1321, 1281, 1250, 1217, 1180, 1141, 1111, 1081, 1047, 936, 925, 897, 857, 809, 781, 740, 697, 645, 608, 559, 536, 493, 474, 458, 419 cm^{-1}; HRMS (ESI) found: [M+Na^+] m/z = 617.1335, calcd. for $C_{34}H_{27}NaO_8P^+$ = 617.1336.

3,5-bis(benzo[d][1,3]dioxol-5-yl)phenyl dihydrogen phosphate (**18**)

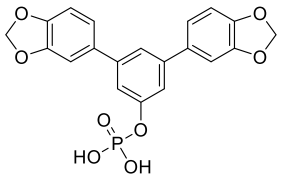

18 was obtained from **18b** (200 mg, 0.33 mmol) according to Method 4. After lyophilization, the product was obtained as fluffy off-white solid (135 mg, 97 %). Decomposition point: 190 °C; ^{1}H NMR (400 MHz, DMSO-d_6) δ = 6.07 (s, 4H), 6.99 (d, *J*=8.0 Hz, 2H), 7.19 (d, *J*=8.0 Hz, 2H), 7.32 (d, *J*=8.3 Hz, 4H), 7.50 (s, 1H) ppm; ^{13}C NMR (101 MHz, DMSO-d_6) δ = 101.48, 107.37, 108.56, 116.67 (m), 119.73, 120.53, 133.96, 141.61, 147.04, 147.93, 153.03 ppm; ^{31}P NMR (162 MHz, DMSO-d_6) δ = -5.5 (s, 1P) ppm; UV/Vis: λ (nm) = 269, 201; IR (KBr): $\tilde{\nu}$ = 3446, 2961, 2898, 2785, 2359, 2336, 2322, 1749, 1733, 1715, 1622, 1604, 1587, 1505, 1494, 1462, 1428, 1413, 1337, 1321, 1248, 1218, 1179, 1140, 1118, 1040, 1006, 984, 939, 926, 878, 859, 817, 780, 720, 699, 669, 648, 613, 579, 558, 537, 499, 419 cm^{-1}; HRMS (ESI) found: [M-H^+] m/z = 413.0434, calcd. for $C_{20}H_{14}O_8P^-$: 413.0432.

3,5-bis(2,3-dihydrobenzo[b][1,4]dioxin-6-yl)phenol (**19a**)

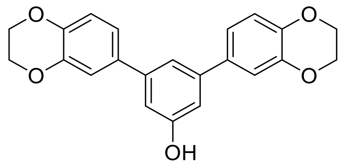

19a was obtained from 3,5-dibromophenol (150 mg, 0.60 mmol) and 1,4-benzodioxane-6-boronic acid (268 mg, 1.49 mmol) according to Method 1. The crude product was purified by column chromatography (DCM/MeOH 200:1) to afford **19a** as off-white solid (160 mg, 74 %). Melting point = 234 °C; R_f = 0.38 (DCM/MeOH 100:1); ^{1}H NMR (400 MHz, $CDCl_3$) δ = 4.27 (s, 8H), 6.87 – 6.93 (4H), 7.05 – 7.12 (4H), 7.17 – 7.20 (1H) ppm; ^{13}C NMR (101 MHz, $CDCl_3$) δ = 64.54, 112.39, 115.95, 117.39, 117.53, 120.27, 134.80, 142.48, 143.30, 143.66, 157.20 ppm; UV/Vis: λ (nm) = 295, 259, 218; IR (KBr): $\tilde{\nu}$ = 3531, 3442, 3044, 2984, 2931, 2875, 1610, 1596, 1582, 1514, 1483, 1456, 1421, 1400, 1348, 1303, 1284, 1249, 1224, 1193, 1166, 1128, 1104, 1068, 1046, 996, 966, 947, 939, 927, 917, 887, 859, 853, 842, 817, 774, 744, 701, 675, 657, 641, 597, 580, 536, 511, 475, 459, 419 cm^{-1}; HRMS (ESI) found: [M+H^+] m/z = 363.1230, calcd. for $C_{22}H_{19}O_5^+$: 363.1227.

Dibenzyl (3,5-bis(2,3-dihydrobenzo[b][1,4]dioxin-6-yl)phenyl) phosphate (**19b**)

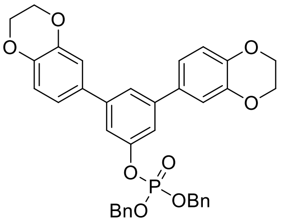

19b was obtained from **19a** (160 mg, 0.44 mmol) according to Method 3. The crude product was purified by column chromatography (hexane/ethyl acetate 9:1 → 7:3) yielding **19b** as colorless oil (240 mg, 87 %). R_f = 0.15 (hexane/ethyl acetate, 7:3); ^{1}H NMR (400 MHz, $CDCl_3$) δ = 4.30 (s, 8H), 5.20 - 5.23 (m, 4H), 6.95 (d, *J*=8.4 Hz, 2H), 7.05 (dd, *J*=8.4 Hz, 2.0 Hz, 2H), 7.14 (t, *J*=1.8 Hz, 2H), 7.35 – 7.40 (m, 12H), 7.52 (s, 1H) ppm; ^{13}C NMR (101 MHz, $CDCl_3$) δ = 64.42 (d, *J*=7.9 Hz), 70.06 (d, *J*=5.8 Hz), 115.95, 116.77 (d, *J*=4.7 Hz), 117.57, 120.21, 121.92, 128.10, 128.63, 128.69, 133.54, 135.46 (d, *J*=6.6 Hz), 142.64, 143.61, 143.75, 151.23 (d, *J*=7.1 Hz) ppm; ^{31}P NMR (162 MHz, $CDCl_3$) δ = -6.2 (s, 1P) ppm; UV/Vis: λ (nm) = 295, 265, 205; IR (KBr): $\tilde{\nu}$ = 3501, 3482, 3089, 3064, 3034, 2979, 2933, 2877, 1733, 1607, 1579, 1510, 1455, 1419, 1391, 1337, 1302, 1284, 1260, 1247, 1218, 1199, 1161, 1129, 1104, 1068, 1042, 1007, 984, 932, 918, 898, 886, 863, 814, 787, 773, 744, 697, 675, 648, 611, 598, 580, 536, 493, 475, 462, 418 cm^{-1}; HRMS (ESI) found: [$M+H^+$] m/z = 623.1828, calcd. for $C_{36}H_{32}O_8P^+$: 623.1829.

3,5-bis(2,3-dihydrobenzo[b][1,4]dioxin-6-yl)phenyl dihydrogen phosphate (**19**)

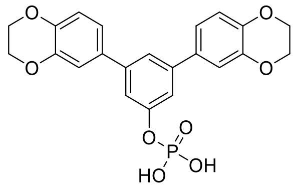

19 was obtained from **19b** (220 mg, 0.35 mmol) according to Method 4. After lyophilization, the product was obtained as a fluffy off white solid (152 mg, 97 %). Melting point = 118 °C; ^{1}H NMR (400 MHz, DMSO-d_6) δ = 4.28 (s, 8H), 6.93 (dd, *J*=8.3 Hz, 2.3 Hz, 2H), 7.18 (dt, *J*=8.4 Hz, 2.4 Hz, 2H), 7.22 (s, 1H), 7.29 (s, 2H), 7.49 (s, 1H) ppm; ^{13}C NMR (101 MHz, DMSO-d_6) δ = 64.13 (d, *J*=6.5), 115.36, 116.42 (d, *J*=4.5 Hz), 117.42, 119.48, 119.77, 132.96, 141.43, 143.36, 143.64, 152.85 (d, *J*=5.2 Hz) ppm; ^{31}P NMR (162 MHz, DMSO-d_6) δ = -5.7 (s, 1P) ppm; UV/Vis: λ (nm) = 295, 265, 225; IR (KBr): $\tilde{\nu}$ = 3435, 2979, 2933, 2877, 1607, 1580, 1512, 1455, 1419, 1391, 1304, 1285, 1247, 1221, 1198, 1162, 1129, 1068, 1046, 1008, 988, 958, 931, 918, 887, 862, 814, 772, 745, 699, 610, 580, 550, 538, 504, 492 cm^{-1}; HRMS (ESI) found: [$M-H^+$] m/z = 441.0742, calcd. for $C_{22}H_{18}O_8P^-$: 441.0745.

3,3'',4,4'',5,5''-hexamethoxy-[1,1':3',1''-terphenyl]-5'-ol (**20a**)

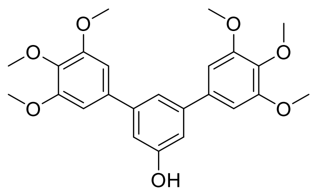

20a was obtained from 3,5-dibromophenol (100 mg, 0.40 mmol) and 3,4,5-trimethoxyphenylboronic acid (253 mg, 1.20 mmol) according to Method 1. The crude product was purified by column chromatography (hexane/ethyl acetate 4:1 → 7:3) to afford **20a** as colorless liquid (108 mg, 64 %). R_f = 0.28 (hexane/ethyl acetate, 3:2); ^{1}H NMR (400 MHz, $CDCl_3$) δ = 3.90 (s, 6H), 3.91 – 3.93 (s, 12H), 6.79 (s, 4H), 6.99 (d, *J*=1.4 Hz, 2H), 7.26 (s, 1H) ppm; ^{13}C NMR (101 MHz, $CDCl_3$) δ = 56.43, 61.14, 104.73, 113.32, 118.88, 136.93, 138.05, 143.80, 153.63, 156.26 ppm; UV/Vis: λ (nm) = 264, 218; IR (KBr): $\tilde{\nu}$ = 3685, 3666, 3653, 3645, 3625, 3380, 3000, 2962, 2939, 2838, 1644, 1583, 1513, 1489, 1463, 1453, 1437, 1426, 1414, 1394, 1339, 1318, 1280, 1239, 1186, 1167, 1127, 1092, 1007, 986, 964, 873, 826, 784, 713, 670, 658; HRMS (ESI) found: [M+H$^+$] m/z = 427.1751, calcd. for $C_{24}H_{27}O_7^+$: 427.1751.

Dibenzyl (3,3'',4,4'',5,5''-hexamethoxy-[1,1':3',1''-terphenyl]-5'-yl) phosphate (**20b**)

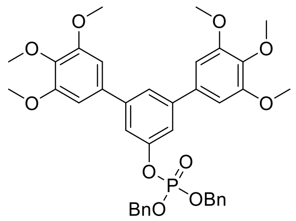

20b was obtained from **20a** (100 mg, 0.23 mmol) according to Method 3. The crude product was purified by column chromatography (hexane/ethyl acetate 9:1 → 13:7), providing **20b** as colorless oil (94 mg, 58 %). R_f = 0.23 (hexane/ethyl acetate, 3:2); ^{1}H NMR (400 MHz, $CDCl_3$) δ = 3.89 (s, 12H), 3.90 (s, 6H), 5.17 (dd, *J*=8.3 Hz, 2.4, 4H), 6.72 (s, 4H), 7.28 - 7.37 (m, 12H), 7.48 (s, 1H) ppm; ^{13}C NMR (101 MHz, $CDCl_3$) δ = 56.47, 61.13, 70.24 (d, *J*=5.7), 104.83, 117.82 (d, *J*=4.9 Hz), 122.89, 128.13, 128.75, 128.84, 135.50 (d, *J*=7.1 Hz), 136.17, 138.32, 143.80, 151.22 (d, *J*=7.1 Hz), 153.70 ppm; ^{31}P NMR (162 MHz, $CDCl_3$) δ = -6.2 (s, 1P) ppm; UV/Vis: λ (nm) = 364, 204; IR (KBr): $\tilde{\nu}$ = 3900, 3868, 3852, 3838, 3818, 3799, 3748, 3734, 3673, 3647, 3627, 3616, 3586, 3565, 3197, 3096, 3032, 2926, 2838, 1716, 1698, 1684, 1670, 1649, 1635, 1610, 1576, 1557, 1541, 1519, 1509, 1490, 1472, 1457, 1438, 1252, 1179, 1030, 827, 699; HRMS (ESI) found: [M+Na$^+$] m/z = 709.2184, calcd. for $C_{38}H_{39}NaO_{10}P^+$ = 709.2173.

3,3'',4,4'',5,5''-hexamethoxy-[1,1':3',1''-terphenyl]-5'-yl dihydrogen phosphate (**20**)

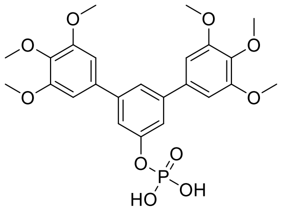

20 was obtained from **20b** (90 mg, 0.13 mmol) according to Method 4. After lyophilization, the product was obtained as a fluffy off white solid (65 mg, 98 %). Melting point = 115 °C; ^{1}H NMR (400 MHz, DMSO-d_6) δ = 3.70 (s, 6H), 3.86 (s, 12H), 6.93 (s, 4H), 7.39 (s, 2H), 7.64 (s, 1H) ppm; ^{13}C NMR (101 MHz, DMSO-d_6) δ = 56.03, 60.05, 104.63, 117.48, 121.01, 135.55, 137.44, 142.39, 152.41 (d, *J*=5.7 Hz), 153.17 ppm; ^{31}P NMR (162 MHz, DMSO-d_6) δ = -5.8 (s, 1P) ppm; UV/Vis: λ (nm) = 269, 222, 201; IR (KBr): $\tilde{\nu}$ = 3444, 2939, 2361, 2338, 1732, 1634, 1583, 1510, 1466, 1432, 1411, 1392, 1330, 1270, 1243, 1182, 1164, 1126, 1006, 991, 853, 830, 783, 703, 669, 624, 615, 570, 552, 529, 522, 513, 499, 487, 475, 468, 452, 438; HRMS (ESI) found: [M+H$^+$] m/z = 507.1395, calcd. for $C_{24}H_{28}O_{10}P^+$ = 507.1415.

2,2'',3,3'',4,4''-hexamethoxy-[1,1':3',1''-terphenyl]-5'-ol **(21a**)

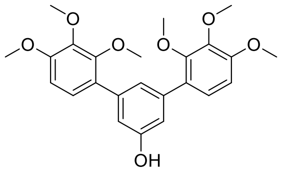

21a was obtained from 3,5-dibromophenol (150 mg, 0.60 mmol) and 2,3,4-trimethoxyphenylboronic acid (379 mg, 1.79 mmol) according to Method 1. The crude product was purified by column chromatography (hexane/ethyl acetate 19:1 → 17:3) to afford **21a** as pure white solid (210 mg, 83 %). R_f = 0.30 (hexane/ethyl acetate 17:3); Melting point = 168 °C; ^{1}H NMR (400 MHz, $CDCl_3$) δ = 3.71 (s, 6H), 3.89 (s, 6H), 3.93 (s, 6H), 6.73 (d, *J*=8.7 Hz, 2H), 6.97 (d, *J*=1.1 Hz, 2H), 7.06 (d, *J*=8.6 Hz, 2H) 7.22 (s, 1H), ppm; ^{13}C NMR (101 MHz, $CDCl_3$) δ = 56.20, 61.16, 61.21, 107.62, 114.74, 122.83, 125.00, 128.51, 139.53, 142.63, 151.54, 153.29, 155.30 ppm; UV/Vis: λ (nm) = 292, 255, 208; IR (KBr): $\tilde{\nu}$ = 3408, 3025, 2996, 2989, 2972, 2937, 2898, 2840, 2825, 1846, 1608, 1594, 1501, 1466, 1456, 1432, 1400 ,1294, 1274, 1227, 1204, 1188, 1153, 1146, 1109, 1092, 1021, 996, 969, 945, 924, 909, 895, 876, 867, 824, 811, 797, 758, 709, 691, 670 618, 579, 518, 407 cm^{-1}; HRMS (ESI) found: [M+Na$^+$] m/z = 449.1575, calcd. for $C_{24}H_{26}NaO_7^+$: 449.1571.

Dibenzyl (2,2'',3,3'',4,4''-hexamethoxy-[1,1':3',1''-terphenyl]-5'-yl) phosphate (**21b**)

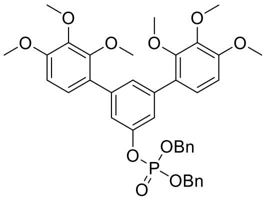

21b was obtained from **21a** (140 mg, 0.33 mmol) according to Method 3. The crude product was purified by column chromatography (hexane/ethyl acetate 9:1 → 3:1), providing **21b** as colorless oil (198 mg, 88 %). R_f = 0.18 (hexane/ethyl acetate, 7:3); ^{1}H NMR (400 MHz, $CDCl_3$) δ = 3.67 (s, 6H), 3.90 (s, 6H), 3.92 (s, 6H), 5.16 (d, *J*=8.1 Hz, 4H), 6.72 (d, *J*=8.7 Hz, 2H), 7.01 (d, *J*=8.6 Hz, 2H), 7.27 – 7.38 (m, 12H), 7.49 (s, 1H) ppm; ^{13}C NMR (101 MHz, $CDCl_3$) δ = 56.21, 61.13, 61.17, 70.04 (d, *J*=5.7 Hz), 107.60, 119.18 (d, *J*=4.8 Hz), 125.00, 126.98, 127.73, 128.13, 128.69, 135.68 (d, *J*=7.0 Hz), 139.68, 142.67, 150.31 (d, *J*=7.1 Hz), 151.58, 153.54 ppm; ^{31}P NMR (162 MHz, $CDCl_3$) δ = -6.2 (s, 1P) ppm; UV/Vis: λ (nm) = 261, 206; IR (KBr): $\tilde{\nu}$ = 2934, 2838, 1596, 1570, 1497, 1463, 1458, 1431, 1409, 1393, 1292, 1233, 1218, 1187, 1166, 1115, 1092, 1017, 981, 921, 882, 789, 759, 743, 697, 599, 455 cm^{-1}; HRMS (ESI) found: [M+H$^+$] m/z = 687.2352, calcd. for $C_{38}H_{40}O_{10}P^+$: 687.2354.

2,2'',3,3'',4,4''-hexamethoxy-[1,1':3',1''-terphenyl]-5'-yl dihydrogen phosphate (**21**)

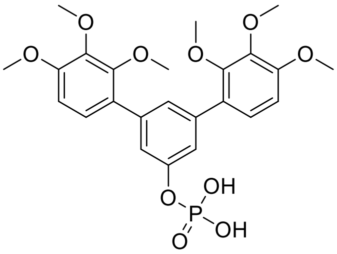

21 was obtained from **21b** (170 mg, 0.25 mmol) according to Method 4. After lyophilization, the product was obtained as a fluffy white solid (122 mg, 97 %). Melting point = 62 °C; ^{1}H NMR (400 MHz, DMSO-*d*$_6$) δ = 3.66 (s, 6H), 3.79 (s, 6H), 3.83 (s, 6H), 6.89 (d, *J*=8.7 Hz, 2H), 7.08 (d, *J*=8.6 Hz, 2H), 7.21 (s, 2H), 7.30 (s, 1H) ppm; ^{13}C NMR (75 MHz, DMSO-*d*$_6$) δ = 55.89, 60.45, 60.82, 108.42, 118.90 (d, *J*=5.0 Hz), 124.56, 126.97, 138.66, 142.10, 150.81, 151.37, 153.16 ppm; ^{31}P NMR (162 MHz, DMSO-*d*$_6$) δ = -5.8 (s, 1P) ppm; UV/Vis: λ (nm) = 257, 218; IR (KBr): $\tilde{\nu}$ = 3610, 3588, 3567, 3545, 3524, 3502, 3445, 3421, 3398, 2955, 2922, 2851, 2376, 2349, 2321, 2311, 1733, 1716, 1605, 1508, 1497, 1465, 1457, 1434, 1417, 1396, 1385, 1291, 1113, 1094, 1026, 786, 762, 669, 641, 597, 490, 453, 418 cm^{-1}; HRMS (ESI) found: [M-H$^+$] m/z = 505.1266, calcd. for $C_{24}H_{26}O_{10}P^-$ = 505.1269.

3,3'',4,4'',5,5''-hexafluoro-[1,1':3',1''-terphenyl]-5'-ol (**22a**)

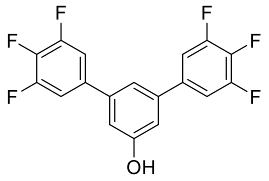

22a was obtained from 3,5-dibromophenol (150 mg, 0.60 mmol) and 3,4,5-trifluorophenylboronic acid (262 mg, 1.49 mmol) according to Method 1. The crude product was purified by column chromatography in DCM to afford **22a** as off-white solid (200 mg, 95 %). R_f = 0.50 (DCM/MeOH 100:1); Melting point = 191 °C, color changed at 155 °C; ^{1}H NMR (400 MHz, $CDCl_3$) δ = 6.99 (d, *J*=1.6 Hz, 2H), 7.06 – 7.08 (m, 1H), 7.14 - 7.23 (m, 4H), 8.17 (s, br, 1H) ppm; ^{13}C NMR (101 MHz, $CDCl_3$) δ = 111.5 - 111.5 (m), 114.20, 117.17, 136.92 (td, *J*=7.8 Hz, 4.6 Hz), 139.57 (dt, *J*=252.3 Hz, 15.3 Hz), 140.5 – 140.7 (m), 151.52 (ddd, *J*=249.8, 10.0, 4.3), 158.09 ppm; ^{19}F NMR (377 MHz, $CDCl_3$) δ = -162.00 (tt, *J*=20.6 Hz, 6.4 Hz, 2F), -133.95 (dd, *J*=20.5 Hz, 8.5 Hz, 4F) ppm; UV/Vis: λ (nm) = 307, 243, 212; IR (KBr): $\tilde{\nu}$ = 3650, 3602, 3423, 3083, 3053, 2372, 2323, 1700, 1619, 1601, 1528, 1490, 1465, 1433, 1402, 1354, 1318, 1280, 1250, 1237, 1211, 1193, 1168, 1078, 1066, 1046, 998, 990, 973, 895, 881, 870, 860, 850, 839, 792, 728, 701, 679, 666, 656, 642, 572, 539, 516, 483 cm^{-1}; HRMS (ESI) found: [M-H^+] m/z = 353.0406, calcd. for $C_{18}H_7F_6O^-$ = 353.0407.

Dibenzyl (3,3'',4,4'',5,5''-hexafluoro-[1,1':3',1''-terphenyl]-5'-yl) phosphate (**22b**)

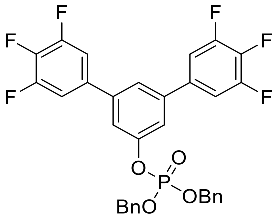

22b was obtained from **22a** (170 mg, 0.48 mmol) according to Method 3. The crude product was purified by column chromatography (hexane/ethyl acetate 9:1 → 4:1), providing **22b** as colorless oil (210 mg, 71 %). R_f = 0.30 (hexane/ethyl acetate 4:1); ^{1}H NMR (400 MHz, $CDCl_3$) δ = 5.14 - 5.29 (m, 4H), 7.05 – 7.15 (m, 4H), 7.18 (s, 2H), 7.31 – 7.43 (m, 11H) ppm; ^{13}C NMR (75 MHz, $CDCl_3$) δ = 70.39 (d, *J*=5.9 Hz), 111.20 - 111.49 (m), 118.66 (d, *J*=4.8 Hz), 122.05, 128.27, 128.76, 128.99, 135.30 (d, *J*=6.4 Hz), 135.61 (td, *J*=7.8 Hz, 4.8 Hz), 139.77 (dt, *J*=253.2 Hz, 15.3 Hz), 140.69, 151.49 (ddd, *J*=250.5 Hz, 10.0 Hz, 4.2 Hz), 151.60 (d, *J*=6.9 Hz) ppm; ^{19}F NMR (377 MHz, $CDCl_3$) δ = -161.07 – -160.93 (m, 2F), -133.34 (dd, *J*=21.3 Hz, 8.7 Hz, 4F) ppm; ^{31}P NMR (162 MHz, $CDCl_3$) δ = -6.2 (s, 1P) ppm; UV/Vis: λ (nm) = 248, 206, 204; IR (KBr): $\tilde{\nu}$ = 3566, 3545, 3458, 3090, 3067, 3037, 2958, 2899, 2322, 1965, 1889, 1816, 1777, 1617, 1592, 1528, 1498, 1459, 1427, 1401, 1386, 1350, 1273, 1251, 1216, 1163, 1083, 1046, 1005, 991, 902, 881, 850, 825, 794, 742, 698, 678, 665, 657, 637, 607, 593, 568, 549,

517, 496, 479, 433 cm^{-1}; HRMS (ESI) found: [M+Na$^+$] m/z = 637.0970, calcd. for $C_{32}H_{21}F_6NaO_4P^+$ = 637.0974.

3,3'',4,4'',5,5''-hexafluoro-[1,1':3',1''-terphenyl]-5'-yl dihydrogen phosphate (**22**)

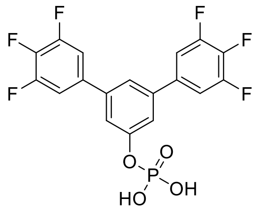

22 was obtained from **22b** (190 mg, 0.31 mmol) according to Method 4. After lyophilization, the product was obtained as a fluffy white solid (130 mg, 97%). Melting point = 194 °C; ^{1}H NMR (400 MHz, DMSO-*d*$_6$) δ = 7.47 (s, 2H), 7.55 (s, 1H), 7.71 (m, 4H) ppm; ^{13}C NMR (101 MHz, DMSO-*d*$_6$) δ = 111.42 (d, *J*=20.9 Hz), 118.28, 118.79, 136.31, 138.23, 138.31 (dt, *J*=249.5 Hz, 16 Hz, 2F), 149.27, 151.72, 154.83 ppm; ^{19}F NMR (377 MHz, DMSO-*d*$_6$) δ = -163.53 - 163.41 (m, 2F), -135.17 (dd, *J*=23.0, 10.4, 4F) ppm; ^{31}P NMR (162 MHz, DMSO-*d*$_6$) δ = -7.1 (s, 1P); UV/Vis: λ (nm) = 247, 202 ppm; IR (KBr): $\tilde{\nu}$ = 3441, 2358, 2322, 1732, 1715, 1619, 1594, 1535, 1468, 1427, 1403, 1389, 1348, 1273, 1247, 1215, 1169, 1066, 1046, 1020, 996, 972, 942, 889, 873, 847, 792, 727, 705, 676, 666, 601, 563, 539, 526, 508, 444 cm^{-1}; HRMS (ESI) found: [M-H$^+$] m/z = 433.0067, calcd. for $C_{18}H_8F_6O_4P^-$ = 433.0070.

3',4',5'-trimethoxy-[1,1'-biphenyl]-3-ol (**23a**)[23]

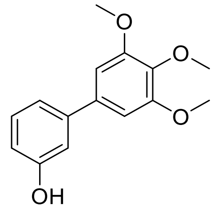

3-bromophenol (130 mg, 0.75 mmol), 3,4,5-trimethoxyphenylboronic acid (239 mg, 1.13 mmol), Na_2CO_3 (120 mg, 1.13 mmol), $Pd(PPh_3)_4$ (26 mg, 0.03 mmol) were placed in a round-bottomed flask. Under an argon atmosphere, a degassed mixture of H_2O/MeOH (3:2 mL) was added. The mixture was stirred at 80 °C for overnight. After cooling down to room temperature, 10 mL of water was added and the reaction mixture was repeatedly extracted with ethyl acetate. The combined organic phases were dried over Na_2SO_4.Volatiles were removed under reduced pressure and purified by column chromatography (hexane/ethyl acetate 9:1 → 4:1) to afford **23a** as colorless oil (180 mg, 92 %). R_f = 0.15 (hexane/ethyl acetate 4:1); ^{1}H NMR (400 MHz, $CDCl_3$) δ = 3.89 (s, 3H), 3.93 (s, 6H), 6.76 (s, 2H), 6.82 (dd, *J*=8.0 Hz, 2.4 Hz, 1H), 7.02 – 7.04 (m, 1H), 7.12 (d, *J*=7.7 Hz, 1H), 7.29 (t, *J*=7.9 Hz, 1H) ppm; ^{13}C NMR (75 MHz, $CDCl_3$) δ = 56.31, 61.12, 104.52, 114.23, 114.38, 119.64, 130.06, 137.00, 137.73, 143.17, 153.49, 156.10 ppm; UV/Vis: λ (nm) = 263, 224; IR (KBr): $\tilde{\nu}$ = 3648, 3419, 3000, 2961, 2938, 2837,

1579, 1515, 1487, 1463, 1447, 1410, 1350, 1268, 1239, 1186, 1164, 1126, 1087, 1075, 1033, 1000, 953, 923, 875, 835, 820, 786, 766, 739, 701, 662, 650, 595, 537, 462 cm^{-1}; HRMS (ESI) found: [M-H^+] m/z = 259.0981, calcd. for $C_{15}H_{15}O_4^-$ = 259.0976.

Dibenzyl (3',4',5'-trimethoxy-[1,1'-biphenyl]-3-yl) phosphate (**23b**)

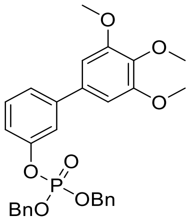

23b was obtained from **23a** (180 mg, 0.69 mmol) according to Method 3. The crude product was purified by column chromatography (hexane/ethyl acetate 9:1 → 7:3), yielding **23b** as colorless oil (320 mg, 89 %). R_f = 0.13 (hexane/ethyl acetate 7:3); ^{1}H NMR (400 MHz, $CDCl_3$) δ = 3.89 (s, 6H), 3.89 (s, 3H), 5.15 (d, *J*=8.3 Hz, 4H), 6.70 (s, 2H), 7.12 – 7.18 (m, 1H), 7.32 (s, 11H), 7.35 (d, *J*=5.0 Hz, 2H) ppm; ^{13}C NMR (101 MHz, $CDCl_3$) δ = 56.38, 61.11, 70.16 (d, *J*=5.8 Hz), 104.65, 118.87 (d, *J*=4.6 Hz), 118.95 (d, *J*=5.2 Hz), 123.93, 128.14, 128.73, 128.80, 130.07, 135.55 (d, *J*=6.9 Hz), 136.09, 138.15, 143.35, 151.03 (d, *J*=6.9 Hz), 153.60 ppm; ^{31}P NMR (162 MHz, $CDCl_3$) δ = -6.2 (s, 1P) ppm; UV/Vis: λ (nm) = 265, 208; IR (KBr): $\tilde{\nu}$ = 3587, 3566, 3545, 3523, 3483, 3088, 3064, 3033, 2997, 2957, 2937, 2899, 2836, 1588, 1576, 1513, 1497, 1483, 1457, 1432, 1403, 1384, 1346, 1281, 1254, 1217, 1183, 1163, 1127, 1083, 1033, 1008, 980, 896, 834, 793, 741, 697, 677, 661, 647, 607, 505, 462 cm^{-1}; HRMS (ESI) found: [M+Na^+] m/z = 543.1537, calcd. for $C_{29}H_{29}NaO_7P^+$ = 543.1543.

3',4',5'-trimethoxy-[1,1'-biphenyl]-3-yl dihydrogen phosphate (**23**)

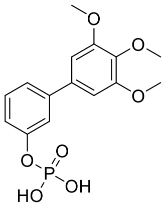

23 was obtained from **23b** (300 mg, 0.58 mmol) according to Method 4. After lyophilization, the product was obtained as fluffy off white solid (188 mg, 96 %). Melting point = 80 °C; ^{1}H NMR (400 MHz, DMSO-d_6) δ = 3.69 (s, 3H), 3.85 (s, 6H), 6.86 (s, 2H), 7.17 (d, *J*=7.6, 1H), 7.31 – 7.45 (m, 3H) ppm; ^{13}C NMR (101 MHz, DMSO-d_6) δ = 55.95, 60.05, 104.22, 118.56, 118.91, 121.84, 129.54, 135.48, 137.28, 141.72, 153.16 ppm; ^{31}P NMR (162 MHz, DMSO-d_6) δ = -5.9 (s, 1P) ppm; UV/Vis: λ (nm) = 264, 213; IR (KBr): $\tilde{\nu}$ = 3464, 2998, 2940, 2838, 2743, 2359, 2339, 2268, 2160, 1748, 1732, 1715, 1697, 1646, 1635, 1593, 1577, 1514, 1484, 1464, 1434, 1403, 1346, 1254, 1184, 1162, 1127, 1087, 1006, 984, 954, 874, 836, 795, 756, 733,

700, 663, 648, 600, 528, 501, 461, 419 cm^{-1}; HRMS (ESI) found: [M-H$^+$] m/z =339.0641, calcd. for $C_{15}H_{16}O_7P^-$: 339.0639.

Diethyl (3,5-dibromobenzyl) phosphonate (**24a**)

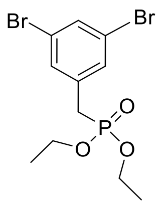

3,5-Dibrombenzylbromide (850 mg, 2.6 mmol) and triethylphosphite (1.34 mL, 7.8 mmol) were heated at 125 °C for 1 h under inert conditions. After completion of the reaction, the mixture was extracted with ethyl acetate, the combined organic layers were washed with brine and dried over MgSO4. Flash column chromatography (acetone in DCM 0 → 8%) was used for purification to obtain a yellow oil (980 mg, 86%). R_f = 0.25 (hexane/ethyl acetate 6:1); ^{1}H-NMR (400 MHz, $CDCl_3$) δ = 7.58 – 7.54 (m, 1H), 7.41 – 7.36 (m, 2H), 4.12 – 3.99 (m, 4H), 3.06 (d, *J* = 21.8 Hz, 2H), 1.28 (t, *J* = 7.0 Hz, 6H) ppm; ^{13}C-NMR (101 MHz, $CDCl_3$) δ = 135.89 (d, *J* = 9.2 Hz), 132.79 (d, *J* = 3.0 Hz), 131.68 (d, *J* = 6.1 Hz), 122.99 (d, *J* = 3.4 Hz), 62.56 (d, *J* = 6.1 Hz), 33.32 (d, *J* = 138.7 Hz), 16.51 (d, *J* = 6.1 Hz) ppm; ^{31}P-NMR (162 MHz, $CDCl_3$) δ = 24.4 ppm; HRMS (ESI): *m/z* calcd for $C_{11}H_{15}Br_2O_3P+Na^+$: 406.9018 [M+Na]$^+$; found: 406.9027.

Diethyl (3,3'',4,4'',5,5''-hexamethoxy-[1,1':3',1''-terphenyl]-5'-yl)methyl) phosphonate (**24b**)

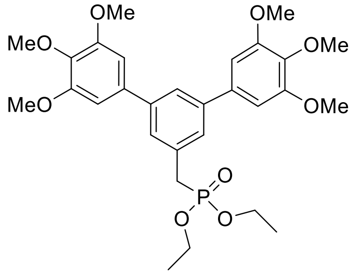

24a (0.8 g, 2.1 mmol), 3,4,5-trimethoxyphenylboronic acid (1.8 g, 8.3 mmol), K_2CO_3 (1.3 g, 9.3 mmol) and $PdCl_2(PPh_3)_2$ (7 mg, 10 µmol) were dissolved in a degassed mixture of dioxane/water (16:4 mL) and refluxed for 20 h. The mixture was extracted with ethyl acetate, washed with water and brine, and dried over Na_2SO_4. The product was purified by flash column chromatography (acetone in DCM 0 → 30 %) to afford an off-white solid (740 mg, 67 %). R_f = 0.25 (acetone/DCM 1:19); Melting point = 140 °C. ^{1}H-NMR (400 MHz, $CDCl_3$) δ = 7.57 (s, 1H), 7.45 (s, 2H), 6.80 (s, 4H), 4.08 (p, *J* = 7.1 Hz, 4H), 3.93 (s, 12H), 3.90 (s, 6H), 3.28 (d, *J* = 21.7 Hz, 2H), 1.28 (t, *J* = 7.1 Hz, 6H) ppm; ^{13}C-NMR (101 MHz, $CDCl_3$) δ =; 153.67, 142.40 (d, *J* = 3.0 Hz), 138.07, 136.96, 132.77 (d, *J* = 8.9 Hz), 127.73 (d, *J* = 6.5 Hz), 124.85 (d, *J* = 3.7 Hz), 104.80, 62.34 (d, *J* = 6.7 Hz), 61.12 (d, *J* = 1.7 Hz), 56.43 (d, *J* = 1.7 Hz), 33.99 (d, *J* = 138.5

Hz), 16.61 (d, J = 6.1 Hz) ppm; ^{31}P-NMR (162 MHz, $CDCl_3$) δ = 26.2 ppm; HRMS (ESI): *m/z* calcd for $C_{29}H_{37}O_9P+H^+$: 561.2248 [M+H]$^+$; found: 561.2250.

Diethyl-(3,3'',4,4'',5,5''-hexamethoxy-[1,1':3',1''-terphenyl]-5'-yl)methyl) phosphonate (**24**)

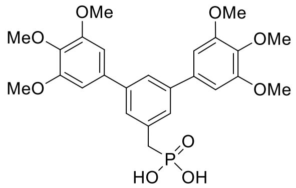

24b (41 mg, 770 µmol) was dissolved in 1.56 mL of dry DCM and cooled to 0 °C. At the same temperature, TMS-Br (91 µL, 693 µmol) was added dropwise. After stirring for 16 h at room temperature, the solvent was removed under reduced pressure. The residue was coevaporated with methanol (3x) and DCM (3x). The crude product was purified by flash column chromatography (methanol/water 2:3). After adding a few drops of water, the pure product was dried by lyophilization overnight and a white solid (39 mg, 100%) was obtained. R_f = 0.40 (methanol/water 3:2); Melting point = 171 °C. ^{1}H-NMR (300 MHz, CD_3OD) δ = 7.60 (s, 2H), 7.51 (s, 1H), 6.98 (s, 4H), 3.93 (s, 12H), 3.81 (s, 6H), 3.08 (d, J = 20.3 Hz, 2H) ppm; ^{13}C-NMR (75 MHz, CD_3OD) δ = 154.67, 142.73, 139.20, 138.63, 128.82 (d, J = 5.2 Hz), 123.95, 105.93, 61.22, 56.80 ppm; ^{31}P-NMR (162 MHz, CD_3OD) δ = 17.93 ppm; UV/Vis: λ (nm) = 203, 218, 265; IR (KBr): $\tilde{\nu}$ = 3433, 3000, 2937, 2833, 1751, 1651, 1583, 1509, 1465, 1411, 1392, 1331, 1241, 1166, 1126, 1003, 918, 829, 782, 765, 671, 551, 528; HRMS (ESI): *m/z* calcd for $C_{25}H_{29}O_9P-H^+$: 503.1476 [*M*-H$^+$]; found: 503.1488.

5‘-Amino-3,3“,4,4“,5,5“-hexamethoxy-1,1‘:3‘,1“-terphenyl (**25a**)

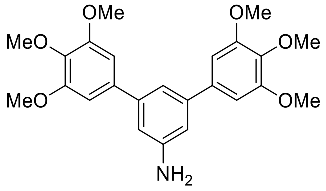

3,5-Dibromoaniline (0.72 g, 2.9 mmol), trimethoxyphenylboronic acid (2.44 g, 11.5 mmol), K_2CO_3 (1.99 g, 14.4 mmol) and $PdCl_2(Ph_3)_2$ (10.1 mg, 14 µmol) were dissolved in a degassed mixture of dioxane/water (15:4 mL) and refluxed overnight. The mixture was extracted with ethyl acetate, washed with brine and dried over Na_2SO_4. The product was purified by flash column chromatography (hexane/ethyl acetate 1:1, 1 % dimethylethylamine) to obtain a brown solid (1.10 g, 90 %). R_f = 0.15 (hexane/ethyl acetate 1:1, 1 % dimethylethylamine); Melting point = 152 °C. ^{1}H-NMR (400 MHz, $CDCl_3$) δ = 7.08 (t, J = 1.6 Hz, 1H), 6.84 (d, J = 1.6 Hz, 2H), 6.79 (s, 4H), 3.92 (s, 12H), 3.89 (s, 6H) ppm; ^{13}C-NMR (101 MHz, $CDCl_3$) δ = 153.39, 146.99, 143.24, 137.72, 137.37, 116.79, 112.94, 104.55, 60.95, 56.24 ppm; UV/Vis: λ (nm) = 319, 261; IR (KBr): $\tilde{\nu}$ = 3527, 3459, 3364, 3228, 3005, 2938 2832, 1582, 1509, 1464, 1395,

1332, 1238, 1126, 1001, 826, 671; HRMS (ESI): *m/z* calcd for $C_{24}H_{27}NO_6+H^+$: 426.1911 $[M+H]^+$; found: 426.1904.

5’-Iodo-3,3“,4,4“,5,5“-hexamethoxy-1,1‘:3‘,1“-terphenyl (**25b**)

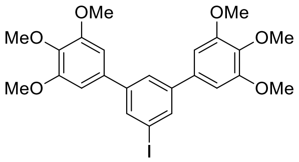

KNO_2 (224 mg, 2.6 mmol) was dissolved in 3.3 mL of DMSO at 35 °C. **25a** (280 mg, 0.7 mmol) was added. A solution of 0.35 mL 57 % HI solution in 3.3 mL of DMSO was added dropwise and stirred for 25 min at room temperature. After cooling to 0 °C, the mixture was neutralized with a 10 % Na_2CO_3 solution. The mixture was extracted with ethyl acetate, washed with brine and dried over MgSO4. The product was purified by flash column chromatography (hexane/ethyl acetate 3:1) to yield a brown solid (272 mg, 77 %). R_f = 0.40 (hexane/ethyl acetate 1:1); Melting point = 137 °C. ^{1}H-NMR (400 MHz, $CDCl_3$) δ = 7.84 (d, *J* = 1.6 Hz, 2H), 7.62 (t, *J* = 1.6 Hz, 1H), 6.75 (s, 4H), 3.93 (s, 12H), 3.90 (s, 6H) ppm; ^{13}C-NMR (101 MHz, $CDCl_3$) δ = 153.74, 144.11, 138.38, 135.69, 135.04, 125.68, 104.76, 95.20, 61.13, 56.50 ppm; UV/Vis: λ (nm) = 273, 241; IR (KBr): $\tilde{\nu}$ = 3445, 2990, 2935, 2832, 1584, 1508, 1405, 1329, 1242, 1127, 1007, 830, 692; HRMS (ESI): *m/z* calcd for $C_{24}H_{25}IO_6+Na^+$: 559.0594 $[M+Na]^+$; found: 559.0594.

Diethyl (Difluoro(3,3'',4,4'',5,5''-hexamethoxy-[1,1':3',1''-terphenyl]-5'-yl)methyl) phosphonate (**25c**)

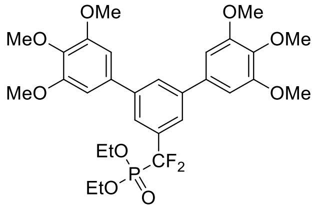

In a fritted funnel, zinc powder was washed 3x with 40 mL of a 1N HCl solution, 5x with 100 mL of H_2O, 3x with 100 mL of MeOH and 3x with 100 mL of Et_2O. The powder was dried overnight under high vacuum. The activated zinc powder (257 mg, 3.9 mmol) was suspended in 9.8 mL of dry THF and heated to 50 °C for 15 min. After cooling to room temperature, the mixture was sonicated for 15 min. Diethyl (bromodifluoromethyl) phosphonate (698 µL, 3.9 mmol) was added and subsequently, the mixture was heated to 50 °C for 1 h. The resulting solution of ((diethoxyphosphoryl)difluoromethyl)-zinc(II)-bromide (0.4 M) was cooled to room temperature and was directly used for the next reaction step.

CuBr (448 mg, 1.0 mmol) was added to the previously described 0.4 M phosphonate solution (5.2 mL, 2.1 mmol) under an argon atmosphere. After stirring for 30 min, **25b** (360 mg, 0.7 mmol) in 5 mL of dry THF was added dropwise and stirred overnight at 45 °C. Ethyl acetate and a saturated NH_4Cl solution were added. The mixture was extracted with ethyl acetate, washed with brine and dried over $MgSO_4$. The product was purified by flash column chromatography (hexane/ethyl acetate 1:1) to obtain an off-white solid (123 mg, 31 %). R_f = 0.25 (hexane/ethyl acetate 1:1); Melting point = 130 °C. ^{1}H-NMR (400 MHz, $CDCl_3$) δ = 7.78 (s, 1H), 7.74 (s, 2H), 6.80 (s, 4H), 4.38 – 4.19 (m, 4H), 3.94 (s, 12H), 3.91 (s, 6H), 1.36 (s, 6H) ppm; ^{13}C-NMR (101 MHz, $CDCl_3$) δ = 153.78, 142.55, 138.37, 136.31, 128.54, 123.99, 104.91, 65.02 (d, *J* = 7.0 Hz), 61.15, 56.50, 16.57 (d, *J* = 5.7 Hz) ppm; ^{19}F-NMR (376 MHz, $CDCl_3$) δ = -107.93 (d, *J* = 115.6 Hz) ppm; ^{31}P-NMR (162 MHz, $CDCl_3$) δ = 6.2 (t, *J* = 115.6 Hz) ppm; HRMS (ESI): *m/z* calcd for $C_{29}H_{35}F_2O_9P+Na^+$: 619.1884 $[M+Na]^+$; found: 619.1878.

<u>(Difluoro(3,3'',4,4'',5,5''-hexamethoxy-[1,1':3',1''-terphenyl]-5'-yl)methyl) phosphonate (**25**)</u>

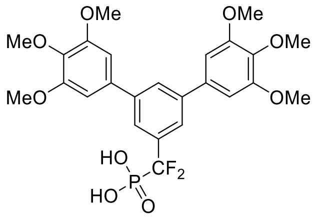

25c (29 mg, 49 µmol) was dissolved in 1 mL of dry DCM. After cooling to 0°C, TMS-Br (58 µL, 438 µmol) was added dropwise and the mixture was stirred for 14 h at room temperature. The solvent was removed under reduced pressure and the residue was coevaporated with methanol (3x) and DCM (3x). The crude product was purified by flash column chromatography (methanol/water 2:3). After adding a few drops of water, the pure product was dried by lyophilization overnight and a white solid (26 mg, 100%) was obtained. R_f (RP) = 0.30 (methanol/water 2:3); Melting point = 154 °C. ^{1}H-NMR (400 MHz, CD_3OD) δ = 7.89 (s, 2H), 7.77 (s, 1H), 7.01 (s, 4H), 3.94 (s, 12H), 3.82 (s, 6H) ppm; ^{13}C-NMR (75 MHz, CD_3OD) δ = 154.76, 142.37, 138.84, 138.83, 126.85, 125.52, 114.54, 106.05, 61.21, 56.84 ppm; ^{19}F-NMR (377 MHz, CD_3OD) δ = -107.73 (d, *J* = 91.9 Hz) ppm; ^{31}P-NMR (162 MHz, CD_3OD) δ = 5.2 (t, *J* = 92 Hz) ppm; HRMS (ESI): *m/z* calcd for $C_{25}H_{27}F_2O_9P-H^+$: 539.1288 $[M-H]^+$; found: 539.1298.

<u>Diethyl ((3,5-dibromophenyl)hydroxymethyl) phosphonate (**26a**)</u>

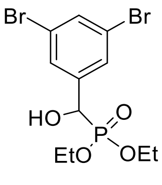

3,5-dibromoanilin (1.00 g, 3.8 mmol) was dissolved in 5 mL of acetonitrile under inert conditions. Diethyl phosphite (0.54 mL, 4.2 mmol) and triethylamine (1.17 mL, 8.4 mmol) were added dropwise and the solution was stirred overnight at room temperature. The solution was extracted with ethyl acetate, washed with brine and dried over $MgSO_4$. The product was purified by flash column chromatography (hexane/ethyl acetate 1:1) to yield an off-white solid (1.16 g, 76 %). R_f = 0.25 (hexane/ethyl acetate 2:1); Melting point = 84 °C. ^{1}H-NMR (400 MHz, $CDCl_3$) δ = 7.64 – 7.58 (m, 3H), 4.97 (d, J = 11.5, 1H), 4.22 – 4.00 (m, 4H), 1.34 – 1.23 (m, 6H) ppm; ^{13}C-NMR (101 MHz, $CDCl_3$) δ = 141.03, 133.56, 128.89, 122.82, 69.70 (d, J = 159.1 Hz), 63.80 (d, J = 53.1 Hz), 16.53 (d, J = 5.6 Hz) ppm; ^{31}P-NMR (162 MHz, $CDCl_3$) δ = 19.77 ppm; HRMS (ESI): *m/z* calcd for $C_{11}H_{15}Br_2O_4P+Na^+$: 422.8967 [*M*+Na]$^+$; found: 422.8968.

<u>Diethyl ((3,5-dibromophenyl)fluoromethyl) phosphonate (**26b**)</u>

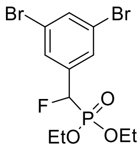

DAST (0.43 mL, 3.3 mmol) was dissolved in 5.5 mL of dry DCM and cooled to -78 °C. At this temperature, **26a** (1.10 g, 2.7 mmol) in 11 mL of dry DCM was added dropwise over 2 h. The solution was slowly warmed to room temperature and then stirred for another 16 h. The solution was subsequently poured into a mixture of 1 mL pyridine in 24 mL ethanol, stirred for 1 h at room temperature and then poured into a mixture of ice and water. The mixture was extracted with ethyl acetate, washed with a 1N HCl solution and brine and dried over $MgSO_4$. The product was purified by flash column chromatography (hexane/diethyl ether 1:2) to obtain a pale yellow solid (0.75 g, 56 %). R_f = 0.40 (hexane/diethyl ether 1:4); Melting point = 61 °C. ^{1}H-NMR (400 MHz, $CDCl_3$) δ = 7.74 – 7.60 (m, 1H), 7.58 – 7.48 (m, 2H), 5.61 (dd, J = 44.7, 8.6 Hz, 1H), 4.26 – 3.99 (m, 4H), 1.45 – 1.16 (m, 6H) ppm; ^{13}C-NMR (101 MHz, $CDCl_3$) δ = 137.04 (d, J = 19.5 Hz), 134.76, 128.34 (dd, J = 7.6, 5.2 Hz), 123.15 (d, J = 2.8 Hz), 87.99 (dd, J = 187.4, 169.1 Hz), 64.05 (dd, J = 41.7, 7.0 Hz), 16.51 (d, J = 5.7 Hz) ppm; ^{19}F-NMR (377 MHz, $CDCl_3$) δ = -203.30 (dd, J = 81.0, 44.7 Hz) ppm; ^{31}P-NMR (162 MHz, $CDCl_3$) δ = 13.4 (d, J = 81.0 Hz) ppm; HRMS (ESI): *m/z* calcd for $C_{11}H_{14}Br_2FO_3P+Na^+$: 424.8923 [*M*+Na]$^+$; found: 424.8928.

Diethyl (monofluoro(3,3'',4,4'',5,5''-hexamethoxy-[1,1':3',1''-terphenyl]-5'-yl)methyl) phosphonate (**26c**)

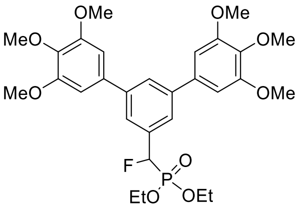

26b (578 mg, 1.4 mmol), 3,4,5-trimethoxyphenylboronic acid (1.213 g, 5.7 mmol), K_2CO_3 (989 mg, 7.2 mmol) and $PdCl_2(PPh_3)_2$ (5 mg, 7 µmol) were dissolved in a degassed mixture of dioxane/water (22:5 mL) and refluxed for 16 h. The solution was extracted with ethyl acetate, washed with a saturated $NaHCO_3$ solution, washed with brine and dried over Na_2SO_4. The product was purified by flash column chromatography (5 % acetone in DCM) to yield a pale yellow solid (437 mg, 54 %). R_f = 0.25 (hexane/ethyl acetate 1:1); Melting point = 88 °C. ^{1}H-NMR (300 MHz, $CDCl_3$) δ = 7.71 – 7.66 (m, 1H), 7.66 – 7.60 (m, 2H), 6.81 (s, 4H), 5.82 (dd, *J* = 44.8, 7.9 Hz, 1H), 4.29 – 4.06 (m, 4H), 3.93 (s, 12H), 3.90 (s, 6H), 1.32 (q, *J* = 7.3 Hz, 6H) ppm; ^{13}C-NMR (101 MHz, $CDCl_3$) δ = 153.72, 142.46, 138.22, 136.59, 134.04 (d, *J* = 18.6 Hz), 126.95, 124.53, 104.83, 89.40 (dd, *J* = 184.5, 169.2 Hz), 63.66 (dd, *J* = 46.9, 6.8 Hz), 61.14, 56.47, 16.47 (dd, *J* = 5.7, 3.4 Hz) ppm; ^{19}F-NMR (377 MHz, $CDCl_3$) δ = -200.66 (dd, *J* = 83.9, 44.8 Hz) ppm; ^{31}P-NMR (162 MHz, $CDCl_3$) δ = 14.78 (d, *J* = 83.9 Hz) ppm; HRMS (ESI): *m/z* calcd for $C_{29}H_{36}FO_9P+Na^+$: 601.1973 [*M*+Na]$^+$; found: 601.1982.

(Monofluoro(3,3'',4,4'',5,5''-hexamethoxy-[1,1':3',1''-terphenyl]-5'-yl)methyl) phosphonate (**26**)

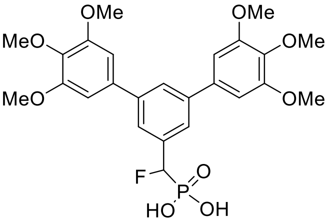

26c (80 mg, 138 µmol) was dissolved in 2.7 mL of dry DCM and cooled to 0 °C. At the same temperature, TMS-Br (165 µL, 929 µmol) was added dropwise. After stirring for 14 h at room temperature, the solvent was evaporated under reduced pressure. The residue was coevaporated with methanol (3x) and DCM (3x). The crude product was purified by flash column chromatography (methanol/water 2:3). After adding a few drops of water, the product was dried by lyophilization overnight and a white solid (72 mg, 100%) was obtained. R_f (RP) = 0.30 (methanol/water 2:3); Melting point = 123 °C. ^{1}H-NMR (400 MHz, CD_3OD) δ = 7.74 (s, 1H), 7.69 (s, 2H), 7.03 – 6.91 (m, 4H), 5.92 – 5.69 (dd, *J* = 45.2, 6.6 Hz, 1H), 3.92 (s, 12H), 3.82 (s, 6H) ppm; ^{13}C-NMR (101 MHz, CD_3OD) δ = 154.83, 143.17, 139.02, 138.34, 137.39 (d, *J* = 17.2 Hz), 126.92, 125.70, 105.93, 61.20, 56.79 ppm; ^{19}F-NMR (377 MHz, CD_3OD) δ =

-200.14 (dd, J = 82.7, 45.2 Hz) ppm; ^{31}P-NMR (162 MHz, CD_3OD) δ = 12.45 ppm (d, J = 82.7 Hz); HRMS (ESI): m/z calcd for $C_{25}H_{28}FO_9P$-H^+: 521.1382 [M-H]$^+$; found: 521.1391.

(((Fluoro(3,3'',4,4'',5,5''-hexamethoxy-[1,1':3',1''-terphenyl]-5'-yl)methyl)phosphoryl)bis(oxy))bis(methylene) bis(2,2-dimethylpropanoate) (**27**)

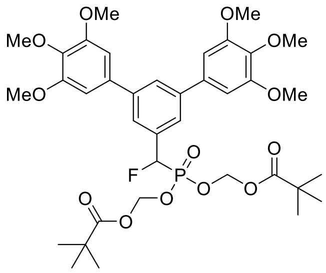

To a solution of **26** (45 mg, 86 µmol) in 2.7 mL of ACN, iodomethyl pivalate (104 µL, 690 µmol) and DIPEA (117 µL, 690 µmol) were added under inert conditions and stirred for 20 h at room temperature. After evaporation of the solvent, flash column chromatography was used for the purification of the product (ethyl acetate in hexane 20 → 40%) to yield a colorless oil (33 mg, 51%). R_f = 0.30 (hexane/ethyl acetate 1:1); ^{1}H-NMR (400 MHz, CD_2Cl_2) δ = 7.77 (s, 1H), 7.59 (s, 2H), 6.84 (s, 4H), 5.87 (dd, J = 44.3, 7.3 Hz, 1H), 5.70 – 5.61 (m, 4H), 3.92 (s, 12H), 3.82 (s, 6H), 1.17 (s, 9H), 1.15 (s, 9H) ppm; ^{13}C-NMR (101 MHz, $CDCl_3$) δ = 176.89 (d, J = 11.1 Hz), 153.76, 142.74 (d, J = 2.3 Hz), 138.30, 136.47, 133.04 (dd, J = 18.7, 1.6 Hz), 127.43, 124.78 (dd, J = 6.2 Hz), 104.96, 89.03 (dd, J = 186.6, 171.5 Hz), 82.14 (dd, J = 11.0, 6.6 Hz), 61.13 (d, J = 1.7 Hz), 56.51 (d, J = 1.5 Hz), 38.84 (d, J = 2.3 Hz), 26.88 (d, J = 3.1 Hz) ppm; ^{19}F NMR (377 MHz, CD_2Cl_2) δ = -200.57 (dd, J = 90.2, 44.3 Hz) ppm; ^{31}P-NMR (162 MHz, CD_2Cl_2) δ = 14.0 (d, J = 90.2 Hz) ppm; UV/Vis: λ (nm) = 220, 269; IR (KBr): $\tilde{\nu}$ = 3448, 2978, 2936, 2830, 1755, 1583, 1509, 1465, 1412, 1394, 1334, 1279, 1241, 1128, 1058, 1006, 957, 891, 832, 768, 710, 670, 605, 585, 565, 529; HRMS (ESI): m/z calcd for $C_{37}H_{48}FO_{13}P$+Na^+: 773.2710 [M+Na]$^+$; found: 773.2733.

NMR spectra

^{1}H NMR of compound **20**

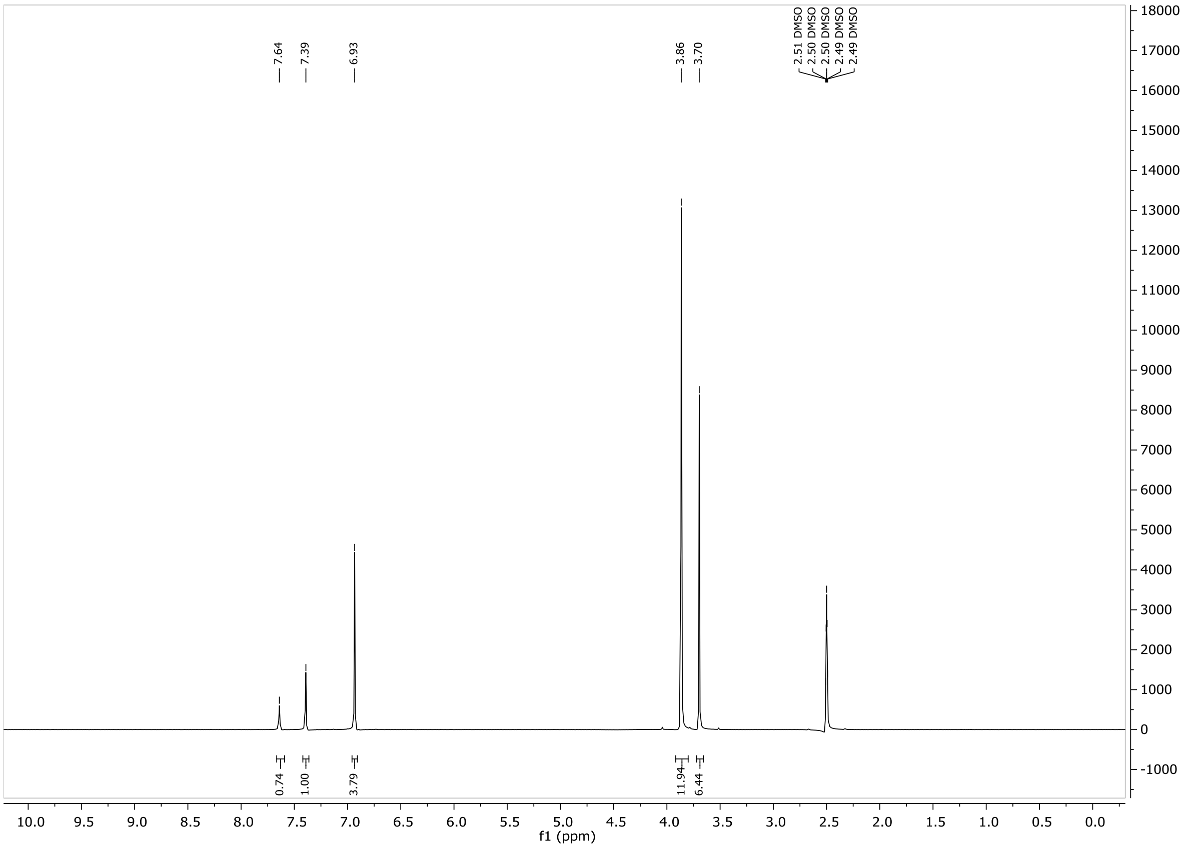

^{13}C NMR of compound **20**

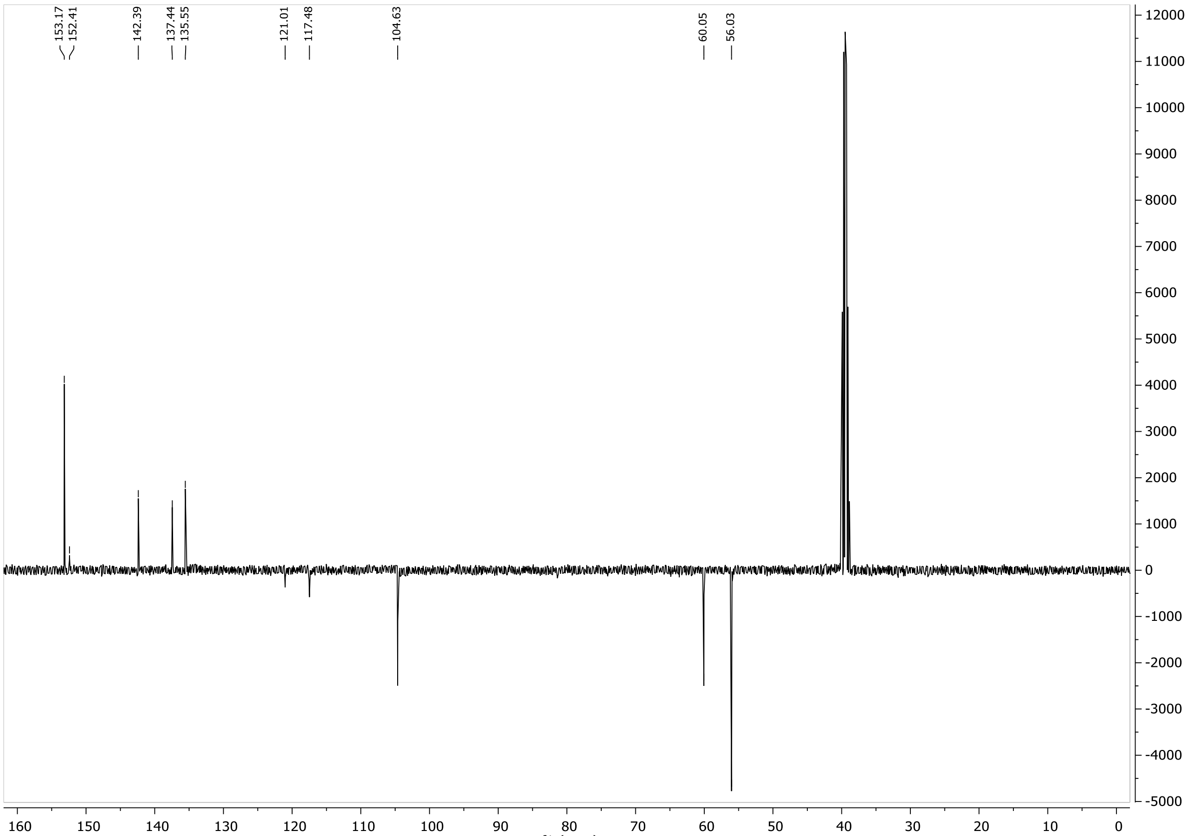

^{31}P NMR of compound **20**

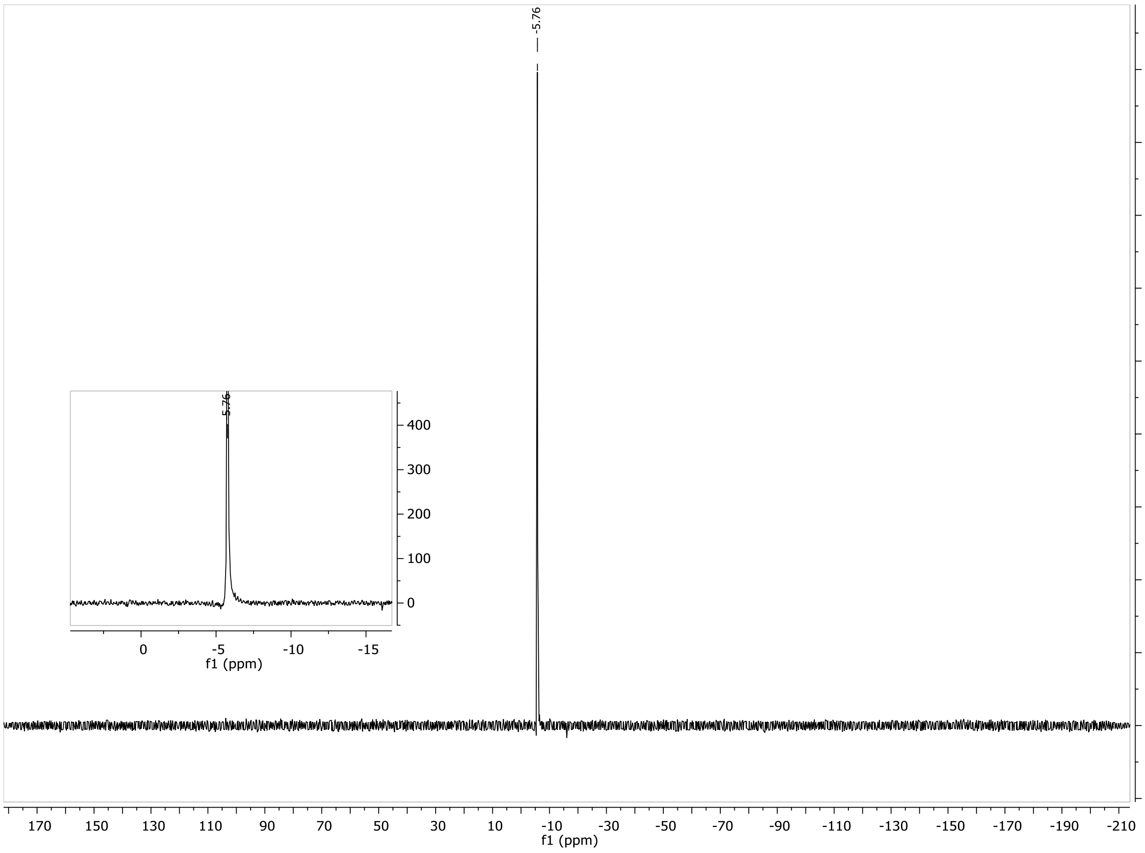

^{1}H NMR of compound **26**

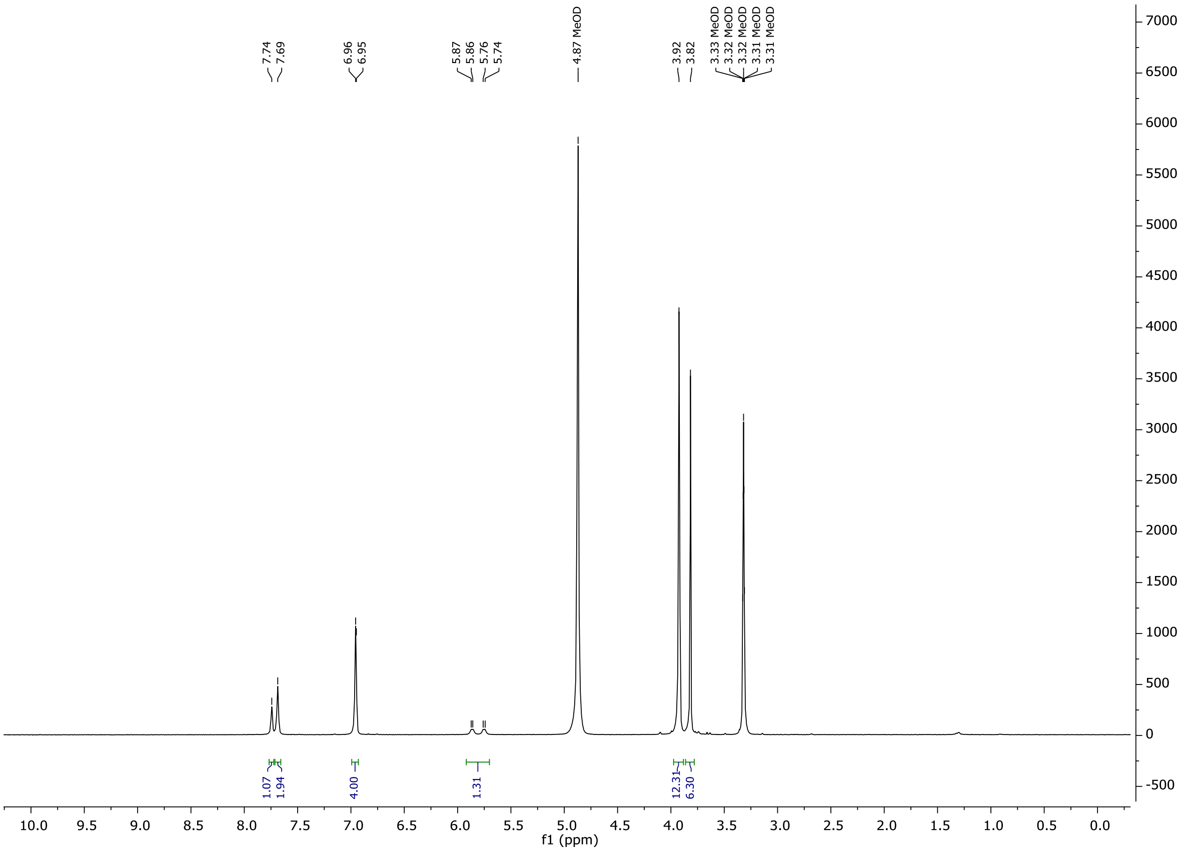

^{13}C NMR of compound **26**

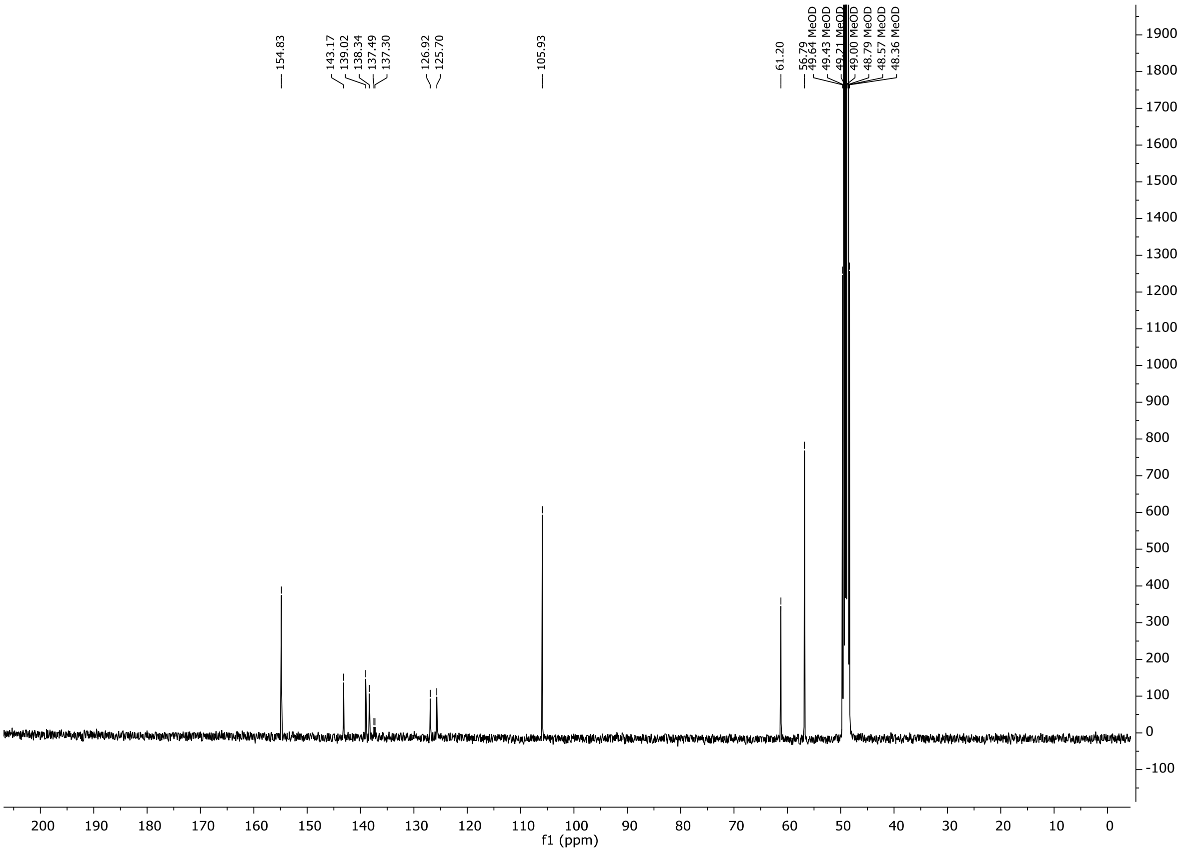

^{31}P NMR of compound **26**

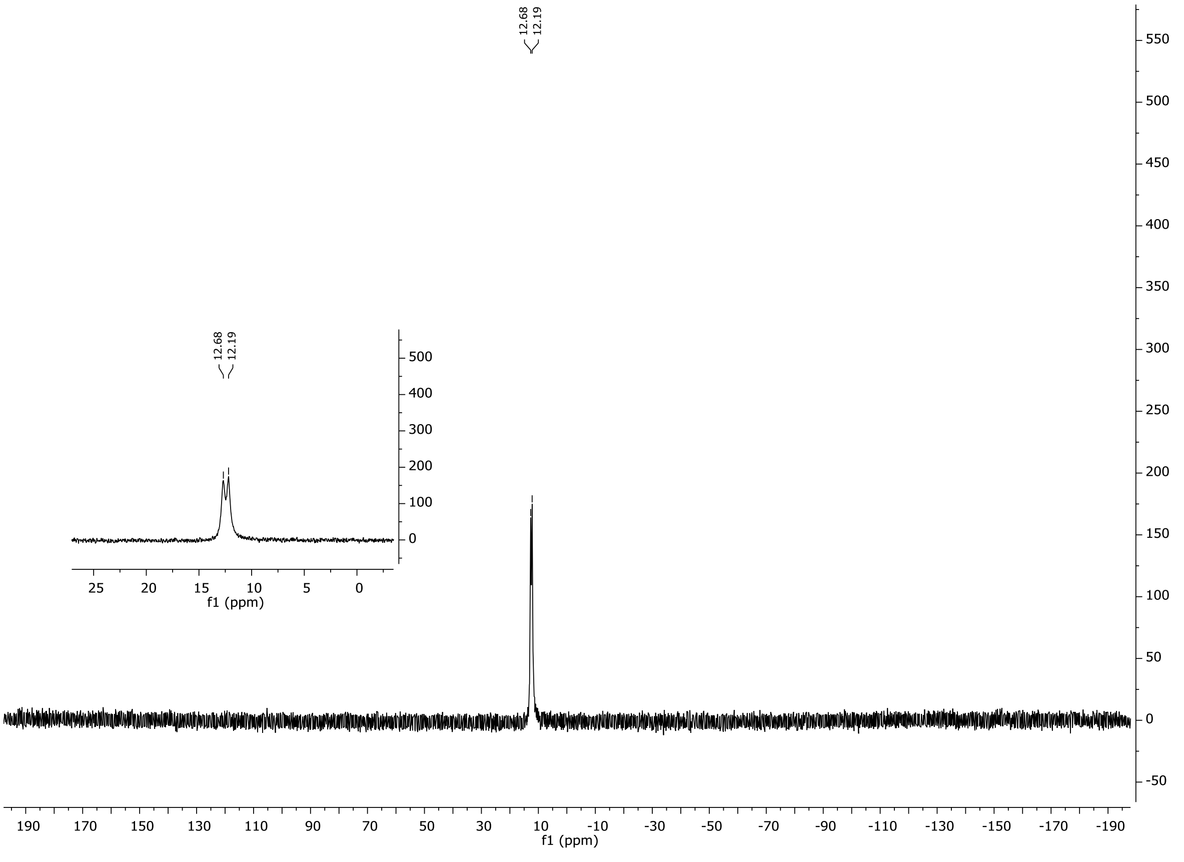

^{1}H NMR of compound **27**

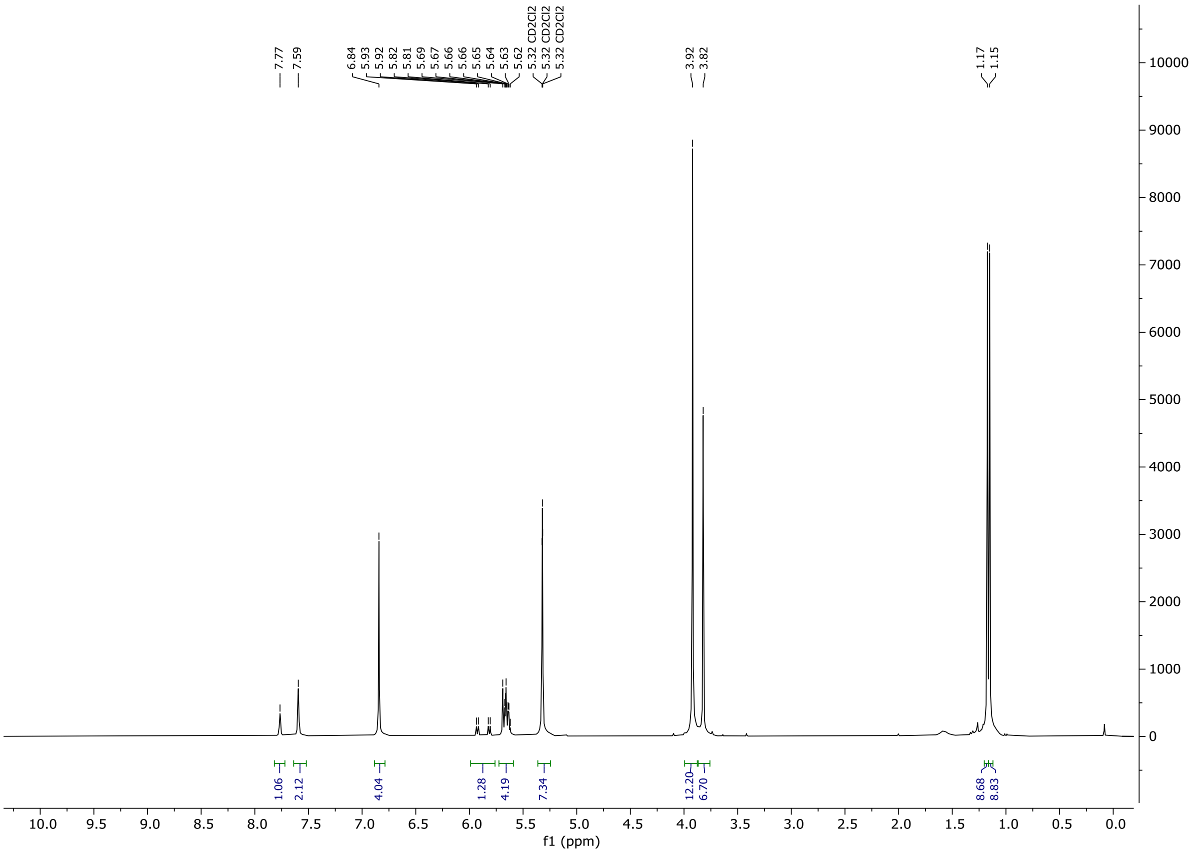

^{13}C NMR of compound **27**

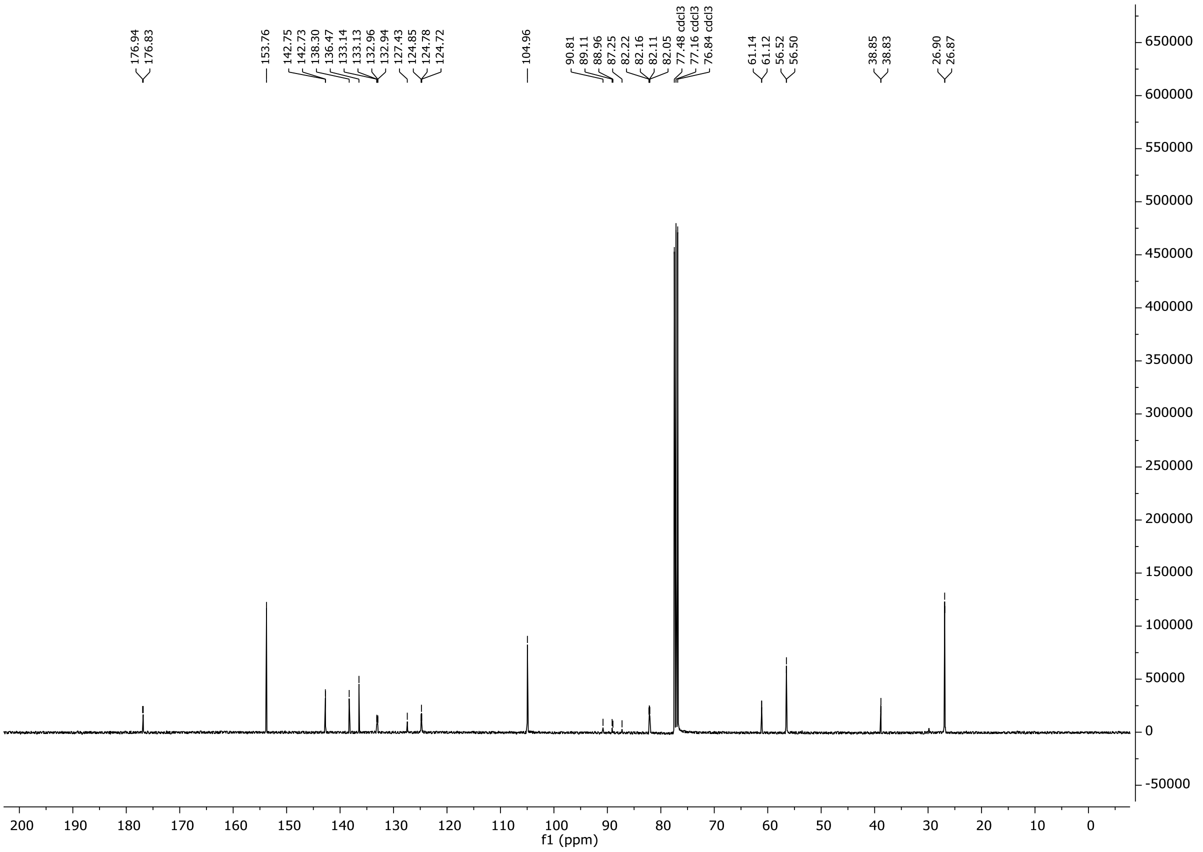

^{31}P NMR of compound **27**

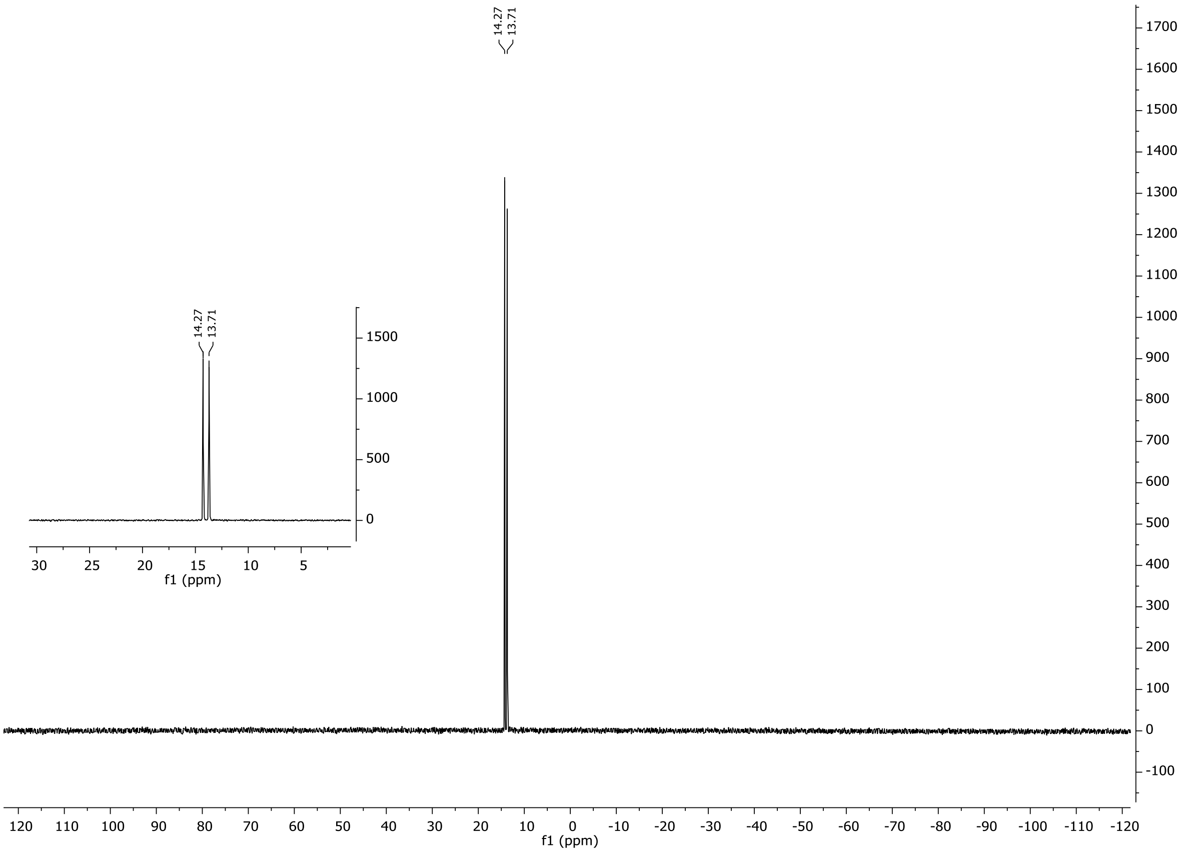

Supporting references

[1] M. A. Koch, A. Schuffenhauer, M. Scheck, S. Wetzel, M. Casaulta, A. Odermatt, P. Ertl, H. Waldmann, *Proc. Natl. Acad. Sci. U. S. A.* **2005**, *102*, 17272-17277.

[2] S. Becker, B. Groner, C. W. Müller, *Nature* **1998**, *394*, 145-151.

[3] The PyMOL Molecular Graphics System, Version 0.99 Schrödinger, LLC.

[4] J. Gräb, A. Berg, L. Blechschmidt, B. Klüver, S. Rubner, D. Y. Fu, J. Meiler, M. Gräber, T. Berg, *ACS Chem. Biol.* **2019**, *14*, 796-805.

[5] D. Neculai, A. M. Neculai, S. Verrier, K. Straub, K. Klumpp, E. Pfitzner, S. Becker, *J. Biol. Chem.* **2005**, *280*, 40782-40787.

[6] J. J. Irwin, T. Sterling, M. M. Mysinger, E. S. Bolstad, R. G. Coleman, *J. Chem. Inf. Model.* **2012**, *52*, 1757-1768.

[7] D. Weininger, *J. Chem. Inf. Comput. Sci.* **1988**, *28*, 31-36.

[8] N. M. O'Boyle, M. Banck, C. A. James, C. Morley, T. Vandermeersch, G. R. Hutchison, *J. Cheminform.* **2011**, *3*, 33.

[9] S. Forli, R. Huey, M. E. Pique, M. F. Sanner, D. S. Goodsell, A. J. Olson, *Nat. Protoc.* **2016**, *11*, 905-919.

[10] M. F. Sanner, *J. Mol. Graph. Model.* **1999**, *17*, 57-61.

[11] O. Trott, A. J. Olson, *J. Comput. Chem.* **2010**, *31*, 455-461.

[12] a) M. Gräber, W. Janczyk, B. Sperl, N. Elumalai, C. Kozany, F. Hausch, T. A. Holak, T. Berg, *ACS Chem. Biol.* **2011**, *6*, 1008-1014; b) B. Sperl, M. H. Seifert, T. Berg, *Bioorg. Med. Chem. Lett.* **2009**, *19*, 3305-3309.

[13] E. D. de Araujo, M. Geletu, P. T. Gunning, *Protein Expr. Purif.* **2017**, *129*, 1-8.

[14] N. Elumalai, A. Berg, K. Natarajan, A. Scharow, T. Berg, *Angew. Chem. Int. Ed.* **2015**, *54*, 4758-4763.
[15] Z. Nikolovska-Coleska, R. Wang, X. Fang, H. Pan, Y. Tomita, P. Li, P. P. Roller, K. Krajewski, N. G. Saito, J. A. Stuckey, S. Wang, *Anal. Biochem.* **2004**, *332*, 261-273.
[16] a) N. Elumalai, A. Berg, S. Rubner, T. Berg, *ACS Chem. Biol.* **2015**, *10*, 2884-2890; b) N. Elumalai, A. Berg, S. Rubner, L. Blechschmidt, C. Song, K. Natarajan, J. Matysik, T. Berg, *Sci. Rep.* **2017**, *7*, 819.
[17] C. A. Schneider, W. S. Rasband, K. W. Eliceiri, *Nat. Methods* **2012**, *9*, 671-675.
[18] G. Bist, N. T. Pun, T. B. Magar, A. Shrestha, H. J. Oh, A. Khakurel, P. H. Park, E. S. Lee, *Bioorg. Med. Chem. Lett.* **2017**, *27*, 1205-1209.
[19] A. R. Katritzky, S. A. Belyakov, S. A. Henderson, P. J. Steel, *J. Org. Chem.* **1997**, *62*, 8215-8217.
[20] D. R. Sexsmith, J. H. Rassweiler, *J. Org. Chem.* **1960**, *25*, 1229-1230.
[21] L. H. Heitman, R. Narlawar, H. de Vries, M. N. Willemsen, D. Wolfram, J. Brussee, A. P. Ijzerman, *J. Med. Chem.* **2009**, *52*, 2036-2042.
[22] D. Trawny, V. Kunz, H.-U. Reissig, *Eur. J. Org. Chem.* **2014**, *2014*, 6295-6302.
[23] K. Bao, Y. Dai, Z. B. Zhu, F. J. Tu, W. G. Zhang, X. S. Yao, *Bioorg. Med. Chem.* **2010**, *18*, 6708-6714.
